# Iron-Catalysed C(*sp*^2^)-H Borylation Enabled by Carboxylate Activation

**DOI:** 10.3390/molecules25040905

**Published:** 2020-02-18

**Authors:** Luke Britton, Jamie H. Docherty, Andrew P. Dominey, Stephen P. Thomas

**Affiliations:** 1 EaStCHEM School of Chemistry, University of Edinburgh, Joseph Black Building, David Brewster Road, Edinburgh EH9 3FJ, UK; s1942174@ed.ac.uk; 2 GSK Medicines Research Centre, Gunnels Wood Road, Stevenage, Hertfordshire SG1 2NY, UK; andrew.q.dominey@gsk.com

**Keywords:** catalysis, borylation, Iron, C-H functionalisation, pinacolborane, photochemistry

## Abstract

Arene C(*sp*^2^)-H bond borylation reactions provide rapid and efficient routes to synthetically versatile boronic esters. While iridium catalysts are well established for this reaction, the discovery and development of methods using Earth-abundant alternatives is limited to just a few examples. Applying an in situ catalyst activation method using air-stable and easily handed reagents, the iron-catalysed C(*sp*^2^)-H borylation reactions of furans and thiophenes under blue light irradiation have been developed. Key reaction intermediates have been prepared and characterised, and suggest two mechanistic pathways are in action involving both C-H metallation and the formation of an iron boryl species.

## 1. Introduction

The development of sustainable methods for the selective C(*sp*^2^)-H functionalisation of arenes is an area of intense research but is still dominated by the use of 2^nd^- and 3^rd^-row transition metals [1,2,3,4,5,6,7]. Earth-abundant metals offer low toxicity and inexpensive alternatives, with iron being a leading example [8,9,10,11,12]. Direct C(*sp*^2^)-H borylation offers a simple and efficient route to aryl-boronic esters, which are key platforms for organic synthesis [13,14,15]. Iridium-based complexes have become a “go-to” for C(*sp*^2^)-H borylation reactions [16,17,18,19,20,21,22,23,24], while the discovery and development of Earth-abundant alternatives remains comparatively rare [25,26,27,28,29,30,31,32,33,34,35,36,37].

**Scheme 1:**
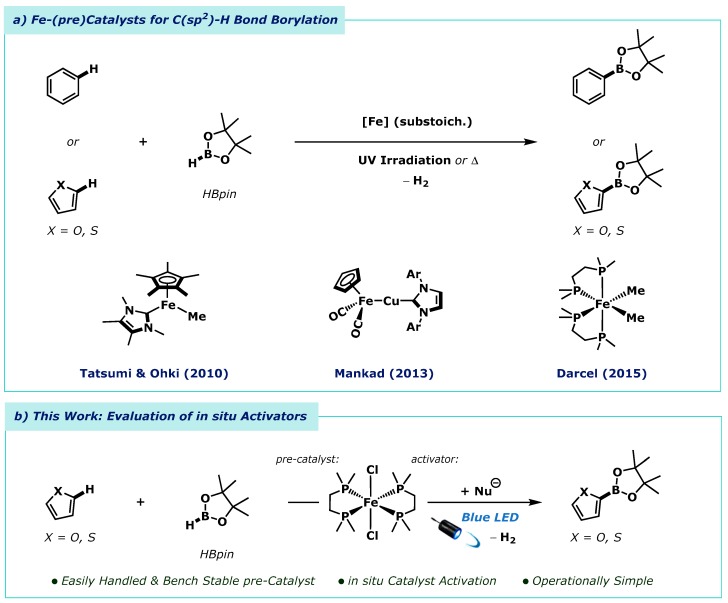
Iron-catalysed C-H borylation of arenes. (**a**) Prior approaches to iron-catalysed C(*sp*^2^)-H-bond borylation with pinacolborane (HBpin) using organoiron and iron/copper bimetallic catalysts. (**b**) This work: C(*sp*^2^)-H bond borylation using dmpe_2_FeCl_2_ as a pre-catalyst, activated by exogenous nucleophiles, under blue light irradiation.

Tatsumi and Ohki showed that arenes would undergo thermally promoted C(*sp*^2^)-H borylation using an *N*-heterocyclic carbene cyclopentadienyl iron(II) alkyl complex [NHC(Cp*)FeMe] as a catalyst in the presence of *tert*-butylethylene (Scheme 1a) [35]. Mankad applied heterobimetallic Fe-Cu and Fe-Zn complexes under continuous ultraviolet light irradiation to arene C(*sp*^2^)-H borylation [36]. Similarly, Darcel and co-workers reported the use of a bis(diphosphino) iron(II) dialkyl and dihydride complexes for arene C(*sp*^2^)-H borylation, again under continuous ultraviolet light irradiation [37]. While these landmark reports are highly significant developments, all require the prior synthesis of sensitive inorganic complexes which are synthetically challenging and difficult to handle for the non-specialist practitioner, thus limiting use by the broader synthetic community.

To reduce the synthetic challenges, and need for organometallic reagents, we questioned whether the C(*sp*^2^)-H borylation chemistry reported previously could be simplified by in situ catalyst activation using only bench stable reagents. In the example reported by Darcel and co-workers the bis[1,2-bis(dimethylphosphino)ethane-*P*,*P*′]dimethyliron(II) pre-catalyst (dmpe_2_FeMe_2_) was generated by the addition of methyllithium to the corresponding iron(II) dichloride complex (dmpe_2_FeCl_2_) [37]. Similarly, the catalytically active bis[1,2-bis(dimethylphosphino)ethane-*P*,*P*′]iron(II) dihydride (dmpe_2_FeH_2_) could be accessed using either LiHBEt_3_ or LiAlH_4_ [37,38]. Given our previous work on the in situ generation of hydride donors formed by the combination of alkoxide salts and pinacolborane (HBpin) [39], we postulated that the active C(*sp*^2^)-H borylation pre-catalyst, dmpe_2_FeH_2_, may be accessible by the same method. Reaction of substoichiometric alkoxide salt with HBpin, the boron source used for this borylation, would generate a hydride reductant in situ to activate the dmpe_2_FeCl_2_ pre-catalyst to dmpe_2_FeH_2_, the active borylation catalyst, and thus initiate catalysis. Importantly, the dmpe_2_FeCl_2_ complex displays much greater air- and moisture stability compared to the dihydride and dialkyl analogues. Herein, we report the in situ activation of dmpe_2_FeCl_2_ and application to the C(*sp*^2^)-H borylation reaction of heteroarenes (Scheme 1b).

## 2. Results

**Scheme 2:**
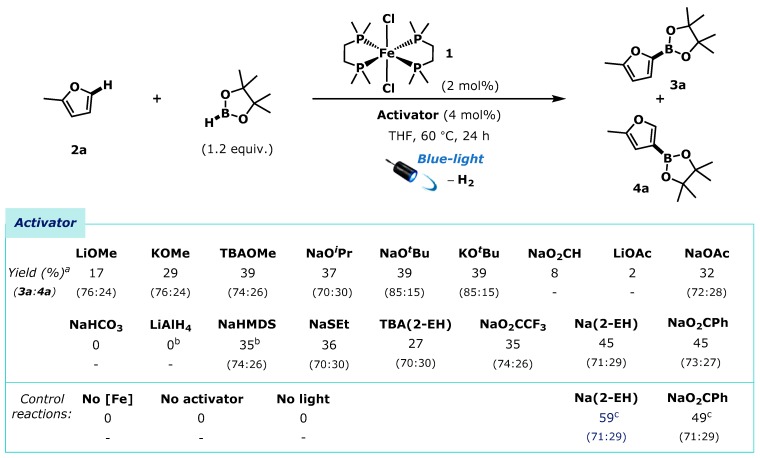
Activator screening for the borylation of 2-methyl furan by dmpe_2_FeCl_2_ **1**. ^a^ Yields determined by ^1^H-NMR spectroscopy of the crude reaction mixtures using 1,3,5-trimethoxybenzene as an internal standard. Product ratios were determined by ^1^H-NMR spectroscopy of the crude reaction mixtures. ^b^ Reaction time = 15 h. ^c^ Reaction time = 48 h. 2-EH = 2-ethylhexanoate. TBA = tetra-*n*-butylammonium.

Guided by the work of Darcel and co-workers, we selected 2-methylfuran **2a** as an ideal test substrate for our investigations. Darcel and co-workers showed that dmpe_2_FeMe_2_ could be used as a pre-catalyst for the borylation of furan **2a** (3 equiv.) using HBpin (1 equiv.) under continuous ultraviolet light irradiation to give a regioisomeric mixture of 5- and 4-borylated furans, **3a** and **4a** respectively (67%, **3a**:**4a** = 82:18) [37]. Using our alkoxide activation strategy we found the use of ultraviolet light for this reaction was not necessary, instead operating with lower energy blue light (Kessil A160 WE, 40 W Blue LED). Additionally, we used an inverted stoichiometry of arene (1 equiv.) and HBpin (1.2 equiv) and a reduced catalyst loading. Using these reaction parameters, we assessed the ability of a selection of potential activators to initiate catalysis alongside the dmpe_2_FeCl_2_ **1** pre-catalyst. (Scheme 2).

Any of LiOMe, KOMe, TBAOMe (TBA = tetra-*n*-butylammonium), NaO*^i^*Pr, NaO*^t^*Bu or KO*^t^*Bu triggered pre-catalyst activation and the formation of both furyl boronic ester regioisomers, **3a** and **4a**, albeit in modest yields (17% to 39%) and with varying regioselectivity, after 24 h. The use of carboxylate salts also initiated catalysis; NaO_2_CH, LiOAc, NaOAc, Na(2-EH) (2-EH = 2-ethylhexanoate), TBA(2-EH), NaO_2_CPh, NaO_2_CCF_3_ all successfully initiated catalysis with varying efficiency (2% to 45%). Na(2-EH) and NaO_2_CPh outperformed all alkoxide salts, and the yields obtained using these activators could be increased with prolonged reaction times to give a mixture of furyl boronic esters **3a** and **4a** in good yield and regioselectivity (Na(2-EH), 59%, **3a**:**4a** = 71:29). Control reactions with no catalyst, no added activator, and with no light irradiation showed no reactivity, highlighting the necessity of each reaction component.

### 2.1. Substrate Scope

**Scheme 3:**
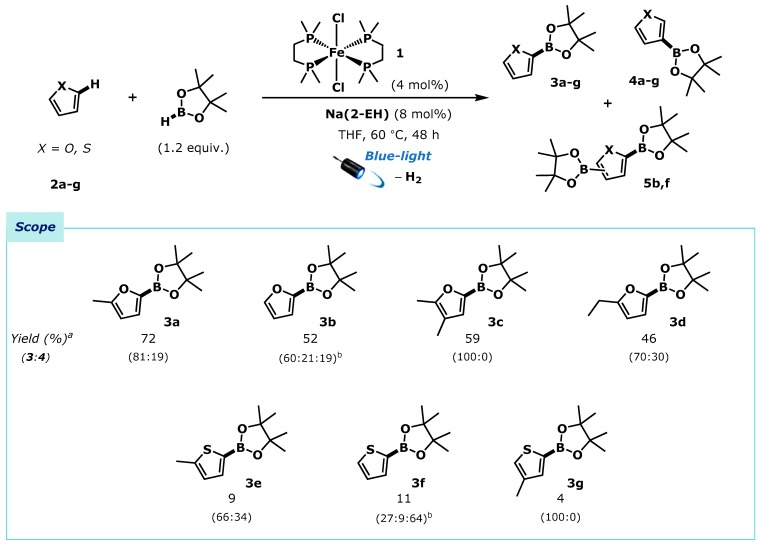
Na(2-EH) activated borylation of furan and thiophene derivatives using dmpe_2_FeCl_2_ **1**. ^a^ Yields determined by ^1^H-NMR spectroscopy of the crude reaction mixtures using 1,3,5-trimethoxybenzene as an internal standard. Product ratios were determined by ^1^H-NMR spectroscopy of the crude reaction mixtures. ^b^ Values represent the ratio of 2-boryl:3-boryl:bis-boryl products.

With optimised reaction conditions established using dmpe_2_FeCl_2_ (4 mol%), Na(2-EH) (8 mol%), arene (1.0 equiv.) and HBpin (1.2 equiv.) in THF under blue light irradiation, we assessed the reactivity of the system by application to a subset of furan and thiophene derivatives (Scheme 3). 2-Methylfuran **2a** underwent efficient borylation to generate a mixture of 5- and 4-borylated regioisomers **3a** and **4a** in good yield and regioselectivity (72%, 81:19). The parent, unsubstituted furan **2b**, also underwent successful borylation but gave a regioisomeric mixture of the 2- and 3-substituted boronic ester regioisomers **3b** and **4b**, and additionally the bis-boryl furans **5b**. Borylation of 2,3-dimethylfuran **2c** gave the corresponding 5-boryl regioisomer **3c** exclusively in good yield. 2-Ethylfuran **2d** reacted similarly to the 2-methylfuran analogue **2a** giving a mixture of 4- and 5-substituted boronic esters **3d** and **4d**. Unfortunately, application to thiophenes demonstrated limited reactivity under the established reaction conditions, giving only low yields of boryl-arenes **3e-g** and **4e-g**, again as a mixture of regioisomers, and bis-borylated product when the parent thiophene was used [40].

### 2.2. Mechanistic Investigations

**Scheme 4:**
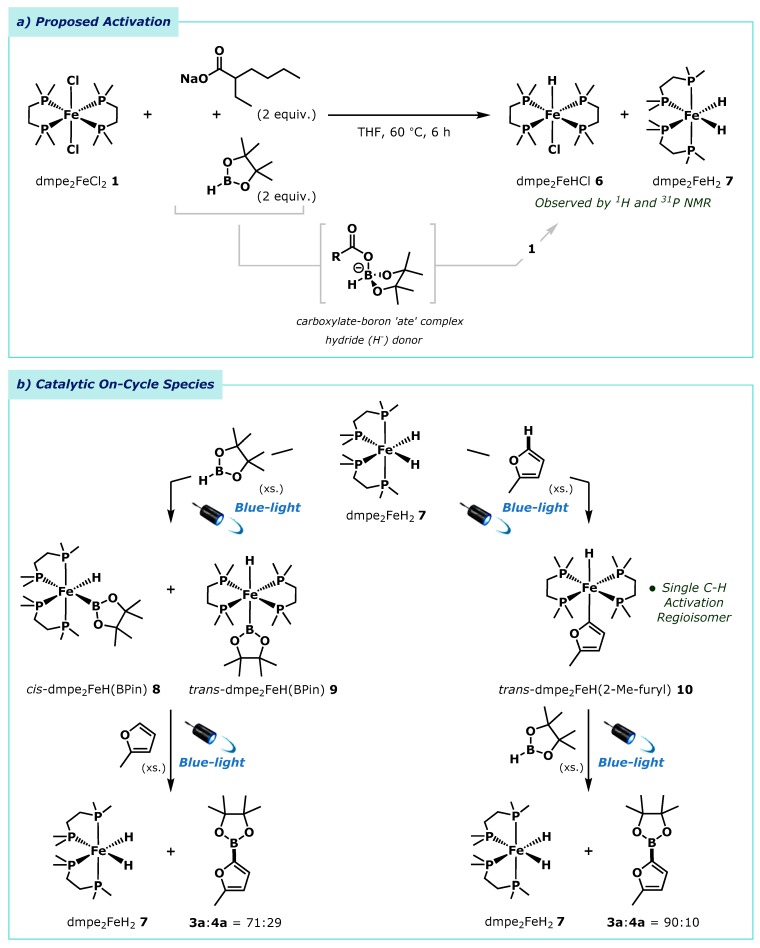
(**a**) Pre-catalyst activation and hydride formation. (**b**) Mechanistic investigations of dmpe_2_FeH_2_ **7** produced by hydride transfer from HBpin and Na(2-EH).

On the basis of successful catalysis we presumed that our in situ activation system provided access to the active iron(II) dihydride complex dmpe_2_FeH_2_ **7**, which had been shown to be catalytically active by Darcel and co-workers [37]. To support this, we combined each component in the absence of light or arene, i.e., the reaction of dmpe_2_FeCl_2_ **1**, Na(2-EH) and HBpin (Scheme 4a). This showed the formation of both the monohydride product dmpe_2_FeHCl **6** and the expected dihydride, dmpe_2_FeH_2_ **7**, as observed by ^31^P-NMR spectroscopy (see Supplementary Materials, S16). Reaction of the activator, Na(2-EH), and HBpin in the absence of pre-catalyst showed ligand redistribution to a mixture of boron-containing species, including boron “ate” complexes, BH_3_ and [BH_4_]^-^, as observed by ^11^B-NMR spectroscopy (see Supplementary Materials, S3). This reactivity is in accordance with that when using other nucleophiles such as alkoxide salts [39,41]. Taken together, these observations are indicative of an in situ activation process, whereby the added carboxylate reagent Na(2-EH) triggers hydride transfer from boron to iron to form the dihydride dmpe_2_FeH_2_ **7**. Once formed, the iron dihydride dmpe_2_FeH_2_ **7** can efficiently catalyse the C(*sp*^2^)-H borylation reaction.

As the dihydride complex dmpe_2_FeH_2_ **7** was readily formed using our in situ hydride transfer method, and was observable by ^1^H and ^31^P-NMR spectroscopy, we next investigated the fundamental steps of this borylation reaction with the aim of identifying key reaction intermediates. Reaction of the in situ generated dmpe_2_FeH_2_ **7** with excess HBpin under blue light irradiation led to the formation of both *cis*-dmpe_2_FeH(Bpin) **8** and *trans*-dmpe_2_FeH(Bpin) **9** boryl iron complexes, as observed by ^1^H, ^11^B, and ^31^P NMR spectroscopy (see Supplementary Materials, S17-19). These complexes were previously reported by Darcel and co-workers, where they were formed from the reaction of the related dialkyl complex, dmpe_2_FeMe_2_, with HBpin [37]. Addition of 2-methylfuran **2a** to the mixture of *cis*-dmpe_2_FeH(Bpin) **8** and *trans*-dmpe_2_FeH(Bpin) **9** under blue light irradiation gave the formation of the regioisomeric furyl boronic esters **3a** and **4a** (**3a**:**4a** = 71:29), notably in a different ratio to that observed during catalysis (vide supra, **3a**:**4a** = 81:19).

Blue light irradiation of the dihydride complex dmpe_2_FeH_2_ **7** in the presence of excess 2-methylfuran **2a** led to exclusive C(*sp*^2^)-H bond metallation at the 5-position to give *trans*-dmpe_2_FeH(2-Me-furyl) **10**, as observed by ^1^H and ^31^P NMR spectroscopy (see Supplementary Materials, S22-24). Addition of HBpin to *trans*-dmpe_2_FeH(2-Me-furyl) **10** and irradiation with blue light induced formation of the furyl boronic esters **3a** and **4a** (**3a**:**4a** = 90:10). Again, in a different ratio to that observed under catalysis. As the ratio of regioisomers observed under catalytic conditions (**3a**:**4a** = 81:19) appears to be a combination of the ratios observed in the stoichiometric studies (**3a**:**4a** = 71:29, and 90:10 respectively), it is suggestive that both the C-H metallation and iron boryl pathways are operative. Specifically, the reaction can precede by C(*sp*^2^)-H bond metallation to give dmpe_2_FeH(2-Me-Furyl) **10**, followed by C(*sp*^2^)-B bond formation, or by direct reaction of arene with the iron boryl species *cis*-dmpe_2_FeH(Bpin) **8** and *trans*-dmpe_2_FeH(Bpin) **9**. The relative ratios of the furyl boronic ester regioisomers indicate both pathways are equally accessible for the activated catalyst. (53% by C-H metallation, 47% by the iron boryl species).

## 3. Conclusions

In summary, we have investigated the applicability of several alkoxide, carboxylate and other, common bench stable reagents towards the in situ activation of an iron(II) pre-catalyst for C(*sp*^2^)-H bond borylation. We found a sodium carboxylate salt Na(2-EH) in combination with HBpin to be a potent pre-catalysts activator generating the iron dihydride dmpe_2_FeH_2_ **7** in situ. The validity of this method was demonstrated by the generation of catalytically relevant species that were used as mechanistic probes. These suggest two C-H borylation pathways are operating to give the aryl boronic ester products; C-H metallation followed by borylation, and formation of an iron boryl species followed by arylation.

## 4. Materials and Methods

### 4.1. General Information

All compounds reported in the manuscript are commercially available or have been previously described in the literature unless indicated otherwise. All experiments involving iron were performed using standard Schlenk techniques under argon or nitrogen atmosphere. All yields refer to yields determined by ^1^H-NMR spectroscopy of crude reaction mixtures using an internal standard. All product ratios refer to product ratios determined by ^1^H-NMR spectroscopy of the crude reaction mixtures. ^1^H-NMR and ^13^C-NMR data are given for all compounds when possible in the experimental section for characterisation purposes. Spectroscopic data matched those reported previously.

### 4.2. Activator Synthesis

Tetra-*n*-butylammonium 2-ethylhexanoate TBA(2-EH)

A suspension of KH (80 mg, 2 mmol) in anhydrous THF (20 mL) was prepared under an N_2_ atmosphere, 2-ethylhexanoic acid (0.32 mL, 2 mmol) was added dropwise whilst stirring. *n.b.* gas evolution (H_2_). The solution was stirred for 3 h at room temperature, and the THF removed in vacuo to give an amorphous colourless solid. The solid was re-dissolved in MeOH (20 mL) and tetra-*n*-butylammonium chloride (556 mg, 2 mmol) was added, the solution was stirred for 16 h, filtered through a glass frit and dried in vacuo without further purification to give tetra-*n*-butylammonium 2-ethylhexanoate (0.72 g, 1.86 mmol, 93%) as an amorphous white solid.

^1^H-NMR (500 MHz, CDCl_3_) δ 3.51–3.35 (m, 8H), 2.09 (tt, *J* = 8.4, 5.4 Hz, 1H), 1.66 (m, 8H), 1.62–1.54 (m, 2H), 1.48–1.39 (m, 8H), 1.39–1.23 (m, 6H), 0.98 (t, *J* = 7.3 Hz, H), 0.91 (t, *J* = 7.4 Hz, 3H), 0.88–0.82 (m, 3H). ^13^C-NMR (126 MHz, CDCl_3_) δ 181.2, 59.0, 51.1, 33.1, 30.5, 26.4, 24.3, 23.2, 19.8, 14.2, 13.7, 12.7.

### 4.3. Pre-catalyst Synthesis

dmpe_2_FeCl_2_ **1** [42]

Anhydrous iron dichloride (0.21 g, 1.67 mmol) was charged to a Schlenk flask and dissolved in anhydrous THF (10 mL), dmpe [(bis(dimethylphosphino)ethane]; 0.50 g, 3.33 mmol) were added to the flask under an Ar atmosphere and the solution left to stir for 48 h at room temperature. The solvent was removed in vacuo, and in an argon-filled glove box, the residue was re-dissolved in dichloromethane (5 mL) and filtered through glass wool. The filtrate was reduced in vacuo to produce a green amorphous solid (0.549 g, 1.29 mmol, 77%).

^1^H-NMR (400 Hz, *d*^8^-THF) δ 2.18 (s, 8H), 1.42 (s, 24H). ^31^P-NMR (202 MHz, CDCl_3_) δ 59.0.

### 4.4. General Borylation Procedure

In an argon-filled glovebox, dmpe_2_FeCl_2_ **1** (8.6 mg, 0.02 mmol), sodium 2-ethylhexanoate (6.6 mg, 0.04 mmol), HBpin (87 µL, 0.6 mmol), substrate (0.5 mmol), and THF (1 mL) were added to a 1.7 mL sample vial and shaken to ensure full dissolution. The vial was placed under blue light radiation for 48 h and then allowed to cool to room temperature. Yields determined by ^1^H-NMR spectroscopy of the crude reaction mixtures using 1,3,5-trimethoxybenzene as an internal standard [0.5 mL; standard solution = 1,3,5-trimethoxybenzene (0.336 g, 2.0 mmol) in diethyl ether (10 mL)]. Product ratios were determined by ^1^H-NMR spectroscopy of the crude reaction mixtures.

### 4.5. Characterisation of Borylated Products

#### 4.5.1. 2-Methylfuran Derivatives

4,4,5,5-Tetramethyl-2-(5-methylfuran-2-yl)-1,3,2-dioxaborolane **3a** [37], 4,4,5,5-tetramethyl-2-(5-methylfuran-3-yl)-1,3,2-dioxaborolane **4a** [37]

Following the general procedure; 2-methylfuran **2a** (41 mg, 44 µL, 0.5 mmol). Yield = 72%. **3a**:**4a** = 81:19. ^1^H-NMR (500 MHz, CDCl_3_) **3a**: δ 6.99 (d, *J* = 3.2 Hz, 1H), 6.06–6.01 (m, 1H), 2.36 (s, 3H), 1.34 (s, 12H). **4a**: δ 7.62 (d, *J* = 0.9 Hz, 1H), 6.15 (t, *J* = 1.0 Hz, 1H), 2.29 (d, *J* = 1.1 Hz, 3H), 1.31 (s, 12H). ^13^C-NMR (126 MHz, CDCl_3_) **3a**: δ 157.8, 124.8, 106.9, 84.0, 24.7, 13.9. **4a**: δ 152.7, 149.7, 108.8, 83.3, 24.9, 13.1. ^11^B-NMR (160 MHz, CDCl_3_) **3a**: δ 27.1. **4a**: δ 29.8.

#### 4.5.2. Furan Derivatives

4,4,5,5-Tetramethyl-2-(furanyl-2-yl)-1,3,2-dioxaborolane **3b** [43], 4,4,5,5-tetramethyl-2-(furanyl-3-yl)-1,3,2-dioxaborolane **4b** [44], 2,5-bis(4,4,5,5-tetramethyl-1,3,2-dioxaborolan-2-yl)furan **5ba** [45], 2,4-bis(4,4,5,5-tetramethyl-1,3,2-dioxaborolan-2-yl)furan **5bb** [45]

Following the general procedure; furan **2b** (34 mg, 36 µL, 0.5 mmol). Yield = 52%. **3b**:**4b**:**5ba**:**5bb** 60:21:4:5. ^1^H-NMR (600 MHz, CDCl_3_) **3b**: δ 7.65 (d, *J* = 1.7 Hz, 1H), 7.07 (d, *J* = 3.4 Hz, 1H), 6.44 (dd, *J* = 3.4, 1.6 Hz, 1H), 1.35 (s, 12H). **4b**: δ 7.78 (s, 1H), 7.46 (m, *J* = 1.6 Hz, 1H), 6.59 (d, *J* = 1.7 Hz, 1H), 1.32 (s, 1H). **5ba**: δ 7.06 (s, 2H), 1.33 (s, 24H). **5bb**: δ 7.78 (s, 1H), 7.28 (s, 1H), 1.30 (s, 24H). ^13^C-NMR (126 MHz, CDCl_3_) **3b**: δ 147.3, 123.2, 110.3, 84.2, 24.8. **4b**: δ 151.2, 142.9, 113.1, 83.5, 24.9. **5ba**: δ 123.2, 83.5, 24.8. **5bb**: δ 151.2, 83.2, 75.1, 24.6. ^11^B-NMR (160 MHz, CDCl_3_) **3b**: δ 27.2. **4b**: δ 29.8.

#### 4.5.3. 2.3-Dimethylfuran Derivatives

4,4,5,5-Tetramethyl-2-(4,5-dimethylfuran-2-yl)-1,3,2-dioxaborolane **3c** [46]

Following the general procedure; 2,3-dimethylfuran **2c** (48 mg, 53 µL, 0.5 mmol). Yield = 46%. **3c**:**4c** = 100:0. ^1^H-NMR (500 MHz, CDCl_3_) δ 6.87 (s, 1H), 2.26 (s, 3H), 1.94 (s, 3H), 1.33 (s, 12H). ^13^C-NMR (126 MHz, CDCl_3_) δ 153.4, 127.1, 115.2, 83.9, 24.7, 11.8, 9.7. ^11^B-NMR (160 MHz, CDCl_3_) δ 27.2.

#### 4.5.4. 2-Ethylfuran Derivatives

4,4,5,5-Tetramethyl-2-(5-ethylfuran-2-yl)-1,3,2-dioxaborolane **3d** [47], 4,4,5,5-tetramethyl-2-(5-methylfuran-3-yl)-1,3,2-dioxaborolane **4d** [47]

Following the general procedure; 2-ethylfuran **2d** (48 mg, 53 µL, 0.5 mmol). Yield = 59%. **3d**:**3d** = 70:30. ^1^H-NMR (500 MHz, CDCl_3_) **3d**: δ 7.01 (d, *J* = 3.3 Hz, 1H), 6.05 (d, *J* = 3.1, 1H), 2.72 (q, *J* = 7.6 Hz, 2H), 1.34 (s, 12H), 1.25 (m, 3H). **4d**: δ 7.64 (d, *J* = 0.8 Hz, 1H), 6.18 (d, *J* = 1.1 Hz, 1H), 2.6 (q, *J* = 7.5, 2H), 1.31 (s, 12H), 1.25 (m, 3H). ^13^C-NMR (126 MHz, CDCl_3_) **3d**: δ 163.6, 124.7, 105.2, 84.0, 24.7, 21.6, 12.2. **4d**: δ 163.6, 149.6, 107.2, 83.3, 24.9, 21.1, 12.1. ^11^B-NMR (160 MHz, CDCl_3_) **3d**: δ 27.2. **4d**: δ 29.9.

#### 4.5.5. 2-Methylthiophene Derivatives

4,4,5,5-Tetramethyl-2-(5-methylthiophen-2-yl)-1,3,2-dioxaborolane **3e** [47], 4,4,5,5-tetramethyl-2-(5-methylthiophen-3-yl)-1,3,2-dioxaborolane **4e** [47]

Following the general procedure; 2-methylthiophene **2e** (49 mg, 48 µL, 0.5 mmol). Yield = 9%. **3e**:**4e** = 66:34. ^1^H-NMR (500 MHz, CDCl_3_) **3e**: δ 7.45 (d, *J* = 3.4 Hz, 1H), 6.84 (d, *J* = 3.4, 1H), 2.53 (s, 3H), 1.33 (s, 12H). **4e**: δ 7.67 (d, *J* = 1.2 Hz, 1H), 7.04 (s, 1H), 2.49 (d, *J* = 1.1 Hz, 3H), 1.32 (s, 12H). ^13^C-NMR (126 MHz, CDCl_3_) **3e**: δ 147.5, 137.6, 127.0, 83.9, 24.9, 15.4. **4e**: not observed. ^11^B-NMR (160 MHz, CDCl_3_) δ 28.7.

#### 4.5.6. Thiophene Derivatives

4,4,5,5-Tetramethyl-2-(thiophen-2-yl)-1,3,2-dioxaborolane **3f** [48], 4,4,5,5-tetramethyl-2-(thiophen-3-yl)-1,3,2-dioxaborolane **4f** [49], 2,5-bis(4,4,5,5-tetramethyl-1,3,2-dioxaborolan-2-yl)thiophene **5f** [48]

Following the general procedure; thiophene **2f** (42 mg, 40 µL, 0.5 mmol). Yield = 11%. **3f**:**4f**:**5f** = 27:9:64. ^1^H-NMR (500 MHz, CDCl_3_) **3f**: δ 7.64 (m, 1H), 7.63 (d, *J* = 4.8 Hz, 1H), 7.19 (d, *J* = 4.8 Hz, 1H), 1.35 (s, 12H). **4f**: δ 7.92 (d, *J* = 2.6 Hz, 1H), 7.41 (d, *J* = 4.9 Hz, 1H), 7.34 (dd, *J* = 4.8, 2.7 Hz, 1H), 1.35 (s, 12H). **5f**: δ 7.66 (s, 2H), 1.34 (s, 24H). ^13^C-NMR (126 MHz, CDCl_3_) **3f**: δ 137.2, 132.4, 128.2, 83.2, 24.9. **4f**: δ 136.5, 129.0, 125.3, 83.2, 24.8. **5f**: δ 137.7, 84.1, 24.8. ^11^B-NMR (160 MHz, CDCl_3_) δ 29.2.

#### 4.5.7. 3-Methylthiophene Derivatives

4,4,5,5-Tetramethyl-2-(4-methylthiophen-2-yl)-1,3,2-dioxaborolane **3g [17]**

Following the general procedure; 3-methylthiophene **2g** (49 mg, 48 µL, 0.5 mmol). Yield = 4%. **3g**:**4g** = 100:0. ^1^H-NMR (500 MHz, CDCl_3_) δ 7.44 (d, *J* = 1.2 Hz, 1H), 7.19 (t, *J* = 1.1 Hz, 1H), 2.29 (d, *J* = 0.9 Hz, 3H), 1.34 (s, 12H). ^13^C-NMR (126 MHz, CDCl_3_) δ 139.5, 139.0, 128.2, 84.0, 24.9, 15.1. ^11^B-NMR (160 MHz, CDCl_3_) δ 29.1.

### 4.6. Mechanistic Investigations

dmpe_2_FeH_2_ **7** [37]

dmpe_2_FeCl_2_ **1** (10 mg, 0.023 mmol), sodium 2-ethylhexanoate (7.6 mg, 0.046 mmol), and HBpin (7 µL, 0.046 mmol) were added to a Young’s NMR tube under an Ar atmosphere and heated at 60 °C for 3 days. ^1^H-NMR (600 MHz, THF) δ −14.38 (m). ^31^P-NMR (500 MHz, THF) δ 76.9 (t, *J* = 28 Hz), 67.7 (t, *J* = 28 Hz).

*cis*-dmpe_2_FeH(Bpin) **8** and *trans*-dmpe_2_FeH(Bpin) **9** [37]

dmpe_2_FeCl_2_ **1** (4.3 mg, 0.001 mmol), sodium 2-ethylhexanoate (3.3 mg, 0.002 mmol), and HBpin (87 µL, 0.6 mmol) were added to a Young’s NMR tube under an Ar atmosphere and irradiated with blue light for 16 h. ^1^H-NMR (500 MHz, THF) δ −13.1 (p, *J* = 43.2 Hz), −14.0 (m). ^31^P-NMR (500 MHz, THF) δ 77.6(m), 77.2 (m), 59.7 (m), 58.9 (m).

dmpe_2_FeH(2-Me-furyl) **10**

dmpe_2_FeCl_2_ **1** (10 mg, 0.023 mmol), sodium 2-ethylhexanoate (30.4 mg, 0.184 mmol), and HBpin (7 µL, 0.046 mmol) were added to a Young’s NMR tube under an Ar atmosphere and warmed at 60 °C for 24 h. 2-methylfuran (8 µL, 0.092 mmol) was added under an Ar atmosphere and the sample irradiated with blue light for 3 h. This complex was observed *in situ*. ^1^H-NMR (500 MHz, THF) δ -18.93 (q, *J* = 45.8 Hz). ^31^P-NMR (500 MHz, THF) δ 77.1 (d, *J* = 38.1 Hz). MS: (HRMS – EI^+^) Found 438.12041 (C_17_H_38_O_1_^56^Fe_1_P_4_), requires 438.12171.

## Supplementary Materials

The following are available online.
