# Supplementary material for: Iron-Catalysed C(sp2)-H Borylation Enabled by Carboxylate Activation
Source: Molecules. 2020 Feb 18;25(4):905. doi: 10.3390/molecules25040905 (PMC7070404; doi:10.3390/molecules25040905)
Supplement: Supplementary file 1 [file molecules-25-00905-s001.pdf]

Supporting information

Iron-Catalysed C(sp^2)-H Borylation: *in situ* Generation of an Active Catalyst

Luke Britton[1], Jamie H. Docherty[1*], Andrew P. Dominey and Stephen P. Thomas[1*]

[1] EaStCHEM School of Chemistry, University of Edinburgh, Joseph Black Building, David Brewster Road, Edinburgh EH9 3FJ, UK.
[2] GSK Medicines Research Centre, Gunnels Wood Road, Stevenage, Hertfordshire SG1 2NY, UK.

Contents

1. General Experimental 3
2. Table S1 – Iron Complexes for the Catalytic Borylation of 2-methylfuran 4
3. Table S2 – Activator Screening 5
4. Table S3 – Variation of Catalyst to Activator Ratio 6
5. Table S4 – Variation of Catalyst Loading and Reaction Time 7
6. Table S5 – Solvent Screening 8
7. Table S6 – HBpin Quantity Screening 9
8. Table S7 – Substrate Scope 10
9. NMR Spectra 11

General experimental

Reaction Setup: All reactions were performed in oven (185 °C) and/or flamed-dried glassware under an atmosphere of anhydrous nitrogen or argon, unless otherwise indicated. All airand moisture sensitive reactions were carried out using standard vacuum line and Schlenk techniques, or in a glovebox with a purified argon atmosphere. All glassware was cleaned using base (KOH, i PrOH) and acid (HClaq) baths. All reported reaction temperatures correspond to external bath temperatures. Room temperature (r.t) was approximately 22 °C.

NMR Spectroscopy: ^{1}H, ^{13}C, ^{11}B, and ^{31}P spectra were recorded on Bruker Avance III 400 and 500 MHz; Bruker PRO 500 MHz; Bruker Avance I 600 MHz spectrometers. Chemical shifts are reported in parts per million (ppm). ^{1}H and ^{13}C NMR spectra were referenced to the residual deuterated solvent peak ($CDCl_3$: 7.26 ppm, 77.00 ppm; d^8-THF: 1.73 ppm, 25.37). Multiplicities are indicated by app. (apparent), br. (broad), s (singlet), d (doublet), t (triplet), q (quartet), quin. (quintet), sext. (sextet), sept. (septet), non. (nonet). Coupling constants, J, are reported in Hertz and rounded to the nearest 0.1 Hz. Integration is provided

Solvents: All solvents for air- and moisture sensitive techniques were obtained from an anhydrous solvent system (Innovative Technology). Reaction solvents tetrahydrofuran (THF) (Fisher, HPLC grade), ether (Et_2O) (Fisher, BHT stabilized ACS grade), and dichloromethane (CH_2Cl_2) (Fisher, unstabilised HPLC grade) were dried by percolation through two columns packed with neutral alumina under a positive pressure of argon. Reaction solvent toluene (ACS grade) was dried by percolation through a column packed with neutral alumina and a column packed with Q5 reactant (supported copper catalyst for scavenging oxygen) under a positive pressure of argon. Reaction solvent ethanol (absolute, VWR) was used as received. Solvents for filtration, transfers, chromatography, and recrystallization were dichloromethane (CH_2Cl_2) (ACS grade, amylene stabilised), diethylether (Et_2O) (Fisher, BHT stabilised ACS grade), ethyl acetate (EtOAc) (Fisher, ACS grade), hexane (Optima), methanol (MeOH) (ACS grade), pentane (ACS grade), and petroleum ether (40–60°C, ACS grade).

Chemicals: All reagents were purchased from Sigma Aldrich, Alfa Aesar, Acros organics, Tokyo Chemical Industries UK, Fluorochem and Apollo Scientific or synthesised within the laboratory. Iron (II) chloride was purchased from Strem Chemicals Inc. (UK); anhydrous iron chloride, 98% (product number 93-2631. Lot 19226800, 44.00000% Fe, expect 44.059%). Sodium *tert*-butoxide (97%) was purchased from Sigma Aldrich (UK). Sulfate buffer refers to aqueous sulfate buffer solution which was prepared by dissolving Na_2SO_4 (1.5 mol) in H_2SO_4 (0.5 mol) and adding water to give a total volume of 2 L.

Table S1. Iron Complexes for the Catalytic Borylation of 2-Methylfuran.[a]

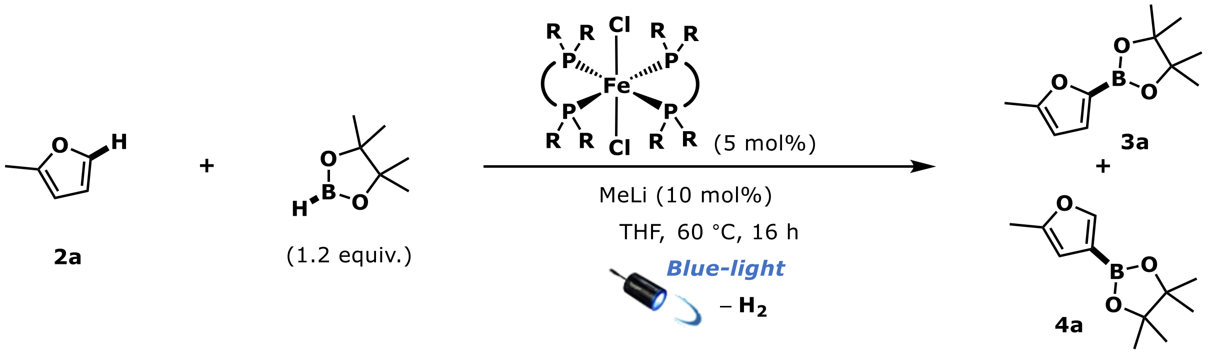

Entry	Pre-catalyst[b]	% 3a	% 4a	3a:4a	% Total
		Yield			
1	$dmpe_2FeCl_2$	12	4	75:25	16
2	$depe_2FeCl_2$	1	0	100:0	1
3	$dmpm_2FeCl_2$	1	0	100:0	1

[a]Experimental conditions: 2-Methylfuran (0.5 mmol), HBpin (0.6 mmol), $dmpe_2FeCl_2$ (5 mol%), MeLi (10 mol%), THF (0.5 mL), 16 hours, blue LED. Yields determined by ^1H NMR spectroscopy of the crude reaction mixtures using 1,3,5-trimethoxybenzene as an internal standard. Product ratios were determined by ^1H NMR spectroscopy of the crude reaction mixtures. [b]dmpe = bis(dimethylphosphino)ethane; depe = bis(diethylphosphino)ethane; dmpm = bis(dimethylphosphino)methane.

Table S2. Activator Screening[a]

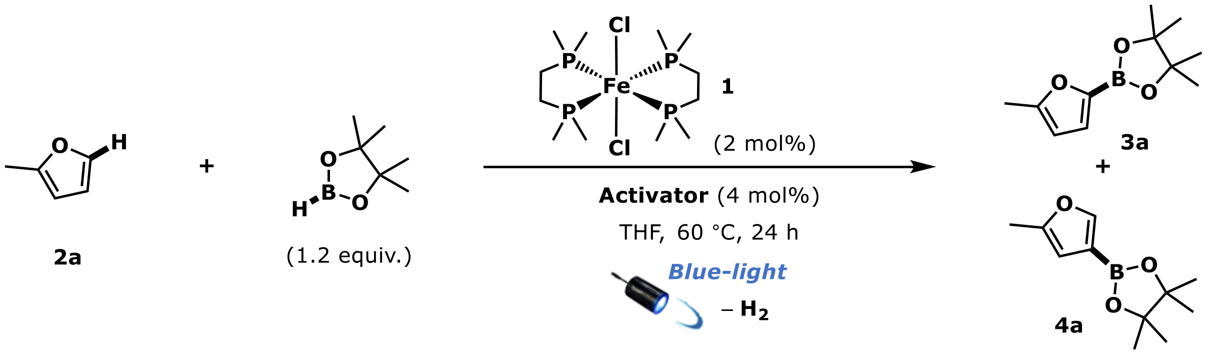

Entry	Activator	Yield[a] % 3a	% 4a	3a:4a	% Total
1	Lithium methoxide	13	4	76:24	17
2	Potassium methoxide	22	7	76:24	29
3	Tetrabutylammonium methoxide	29	10	74:26	39
4	Sodium *iso*-propoxide	24	10	70:30	34
5	Sodium *tert*-butoxide	33	6	85:15	39
6[b]	Sodium *tert*-butoxide	0	0	0	0
7	Potassium *tert* -butoxide	3	6	85:15	39
8	Sodium ethanethiolate	28	8	70:39	36
9[c]	Lithium aluminium hydride	0	0	0	0
10	Sodium bicarbonate	0	0	0	0
11[c]	Sodium bis(trimethylsilyl)amide	28	7	74:26	35
12	Sodium formate	6	2	75:25	8
13	Lithium acetate	2	0	100:0	2
14	Sodium acetate	23	9	72:28	32
15	Sodium trifluroacetate	26	9	74:26	35
16	Sodium benzoate	33	12	73:27	45
17[d]	Sodium benzoate	35	14	73:27	49
18	Sodium 2-ethylhexanoate	32	13	71:29	45
19[e]	Sodium 2-ethylhexanoate	27	10	71:29	37
20	Tetrabutylammonium 2-ethylhexanoate	19	8	70:30	27
21[d]	Sodium 2-ethylhexanoate	45	14	71:29	59
22[f]	Sodium 2-ethylhexanoate	0	0	0	0

[a]Standard reaction conditions: 2-Methylfuran (0.5 mmol), HBpin (0.6 mmol), $dmpe_2FeCl_2$ (2 mol%), **Activator** (4 mol%), THF (0.5 mL), 24 hours, blue LED. Yields determined by ^1H NMR spectroscopy of the crude reaction mixtures using 1,3,5-trimethoxybenzene as an internal standard. Product ratios were determined by ^1H NMR spectroscopy of the crude reaction mixtures. [b]$dmpe_2FeCl_2$ (5 mol%), 16 hours, 70 °C, no light. [c]15 hours. [d]48 hours. [e]Stoichiometric 15-crown-5 added. [f]$dmpe_2FeCl_2$ (0 mol%).

Table S3. Variation of Catalyst to Activator Ratio[a]

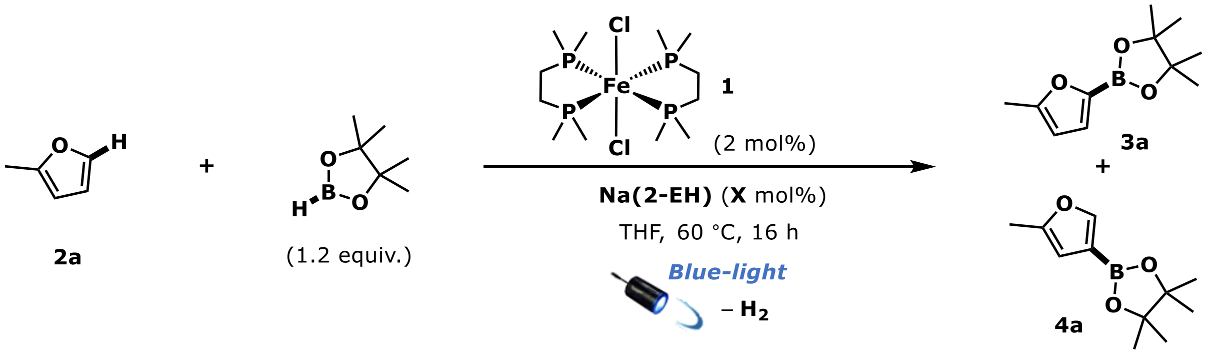

Entry	Catalyst (mol%)	Activator (mol%)	Yield			
			% 3a	% 4a	3a:4a	% Total
1	2	0	0	0	0	0
2	2	2	19	8	70:30	27
3	2	4	35	13	73:27	48
4	2	8	22	9	71:29	31
5	2	16	18	7	72:28	25

[a]Standard reaction conditions: 2-Methylfuran (0.5 mmol), HBpin (0.6 mmol), $dmpe_2FeCl_2$ (**2** mol%), Na(2-EH) (**X** mol%), THF (0.5 mL), 16 hours, blue LED. Yields from crude reaction mixtures determined by ^1H NMR spectroscopy using 1,3,5-trimethoxybenzene as an internal standard. Product ratios were determined by ^1H NMR spectroscopy.

Table S4. Variation of Catalyst Loading and Reaction Time[a]

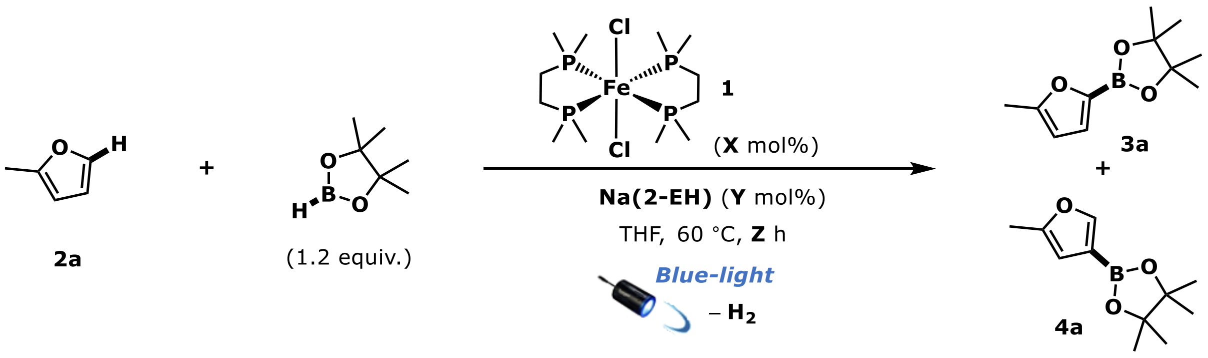

Entry	Catalyst (mol%)	Activator (mol%)	Time (h)	Yield % 3a	% 4a	3a:4a	% Total
1	4	8	24	45	8	85:15	53
2	4	8	48	58	14	81:19	72
3	4	8	67	50	20	71:29	70
4	5	10	24	51	10	84:16	61
5	5	10	48	52	8	87:13	60

[a]Standard reaction conditions: 2-Methylfuran (0.5 mmol), HBpin (0.6 mmol), $dmpe_2FeCl_2$ (**X** mol%), Na(2-EH) (**Y** mol%), THF (0.5 mL), **Z** hours, blue LED. Yields determined by ^1H NMR spectroscopy of the crude reaction mixtures using 1,3,5-trimethoxybenzene as an internal standard. Product ratios were determined by ^1H NMR spectroscopy of the crude reaction mixtures.

Table S5. Solvent Screening[a]

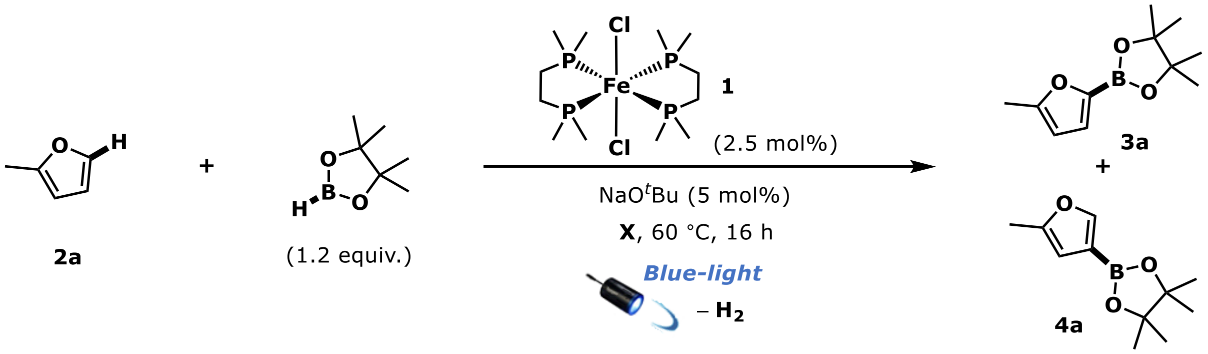

Entry	Solvent	Yield % 3a	% 4a	3a:4a	% Total
1	Tetrahydrofuran	28	25	84:16	33
2	Diethyl ether	29	7	81:19	36
3	Acetonitrile	trace	0	0	/
4	Methanol	0	0	0	0
5	Dichloromethane	0	0	0	0
6	*n*-Hexane	26	5	84:16	31

[a]Standard reaction conditions: 2-Methylfuran (0.5 mmol), HBpin (0.6 mmol), dmpe$_2$FeCl$_2$ (2.5 mol%), NaO^{t}Bu (5 mol%), **X** (0.5 mL), 16 hours, blue LED. Yields determined by ^{1}H NMR spectroscopy of the crude reaction mixtures using 1,3,5-trimethoxybenzene as an internal standard. Product ratios were determined by ^{1}H NMR spectroscopy of the crude reaction mixtures.

Table S6. HBpin Quantity Screening[a]

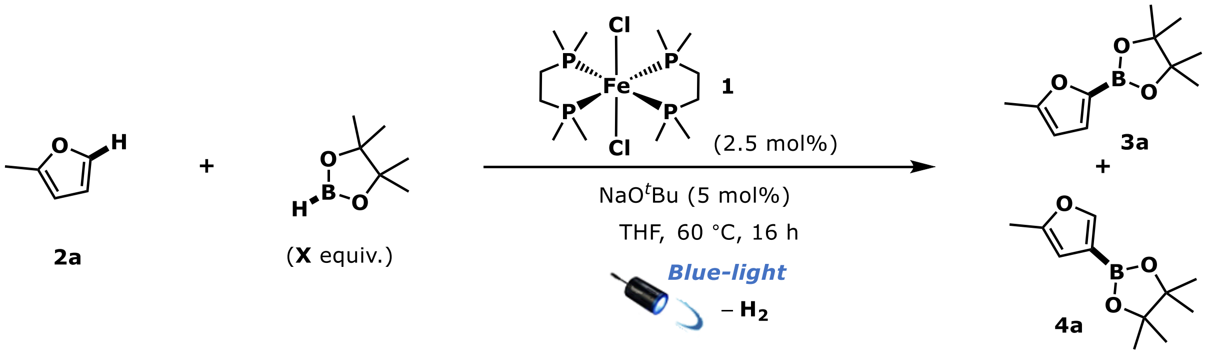

Entry	HBPin equivalents	Yield % 3a	% 4a	3a:4a	% Total
1	1.1	22	4	81:19	26
2	1.2	28	5	84:16	33
3	1.4	14	1	93:7	15
4	1.8	11	1	92:8	12
5	2.2	14	1	93:7	15
6	2.5	8	2	80:20	10

[a]Standard reaction conditions: 2-Methylfuran (0.5 mmol), HBpin (0.6 mmol), $dmpe_2FeCl_2$ (2.5 mol%), NaO^{t}Bu (5 mol%), THF (0.5 mL), 16 hours, blue LED. Yields determined by ^{1}H NMR spectroscopy of the crude reaction mixtures using 1,3,5-trimethoxybenzene as an internal standard. Product ratios were determined by ^{1}H NMR spectroscopy of the crude reaction mixtures.

Table S7. Substrate Scope[a]

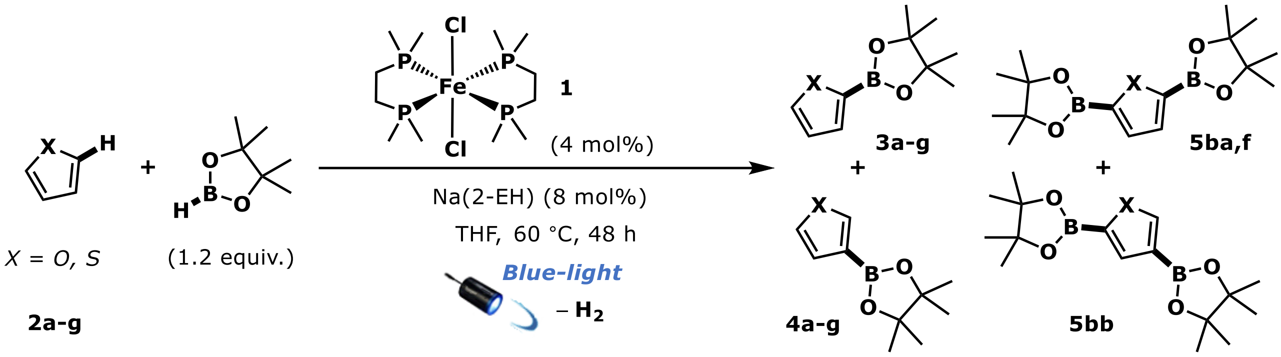

Entry	Substrate	Yield % 3a-g	% 4a-g	% 5ba,f	% 5bb	% Total
1	2-Methylfuran **a**	58	14	0	0	72
2	Furan **b**	31	11	5	5	52
3	2,3-dimethylfuran **c**	59	0	0	0	59
4	2-Ethylfuran **d**	32	14	0	0	46
5	2-Methylthiophene **e**	6	3	0	0	9
6	Thiophene **f**	3	1	7	0	11
7	3-Methylthiophene **g**	4	0	0	0	4

[a]Standard reaction conditions: Substrate (0.5 mmol), HBpin (0.6 mmol), dmpe$_2$FeCl$_2$ (4 mol%), Na(2-EH) (8 mol%), THF (0.5 mL), 48 hours, blue LED. Yields determined by ^{1}H NMR spectroscopy of the crude reaction mixtures using 1,3,5-trimethoxybenzene as an internal standard. Product ratios were determined by ^{1}H NMR spectroscopy of the crude reaction mixtures.

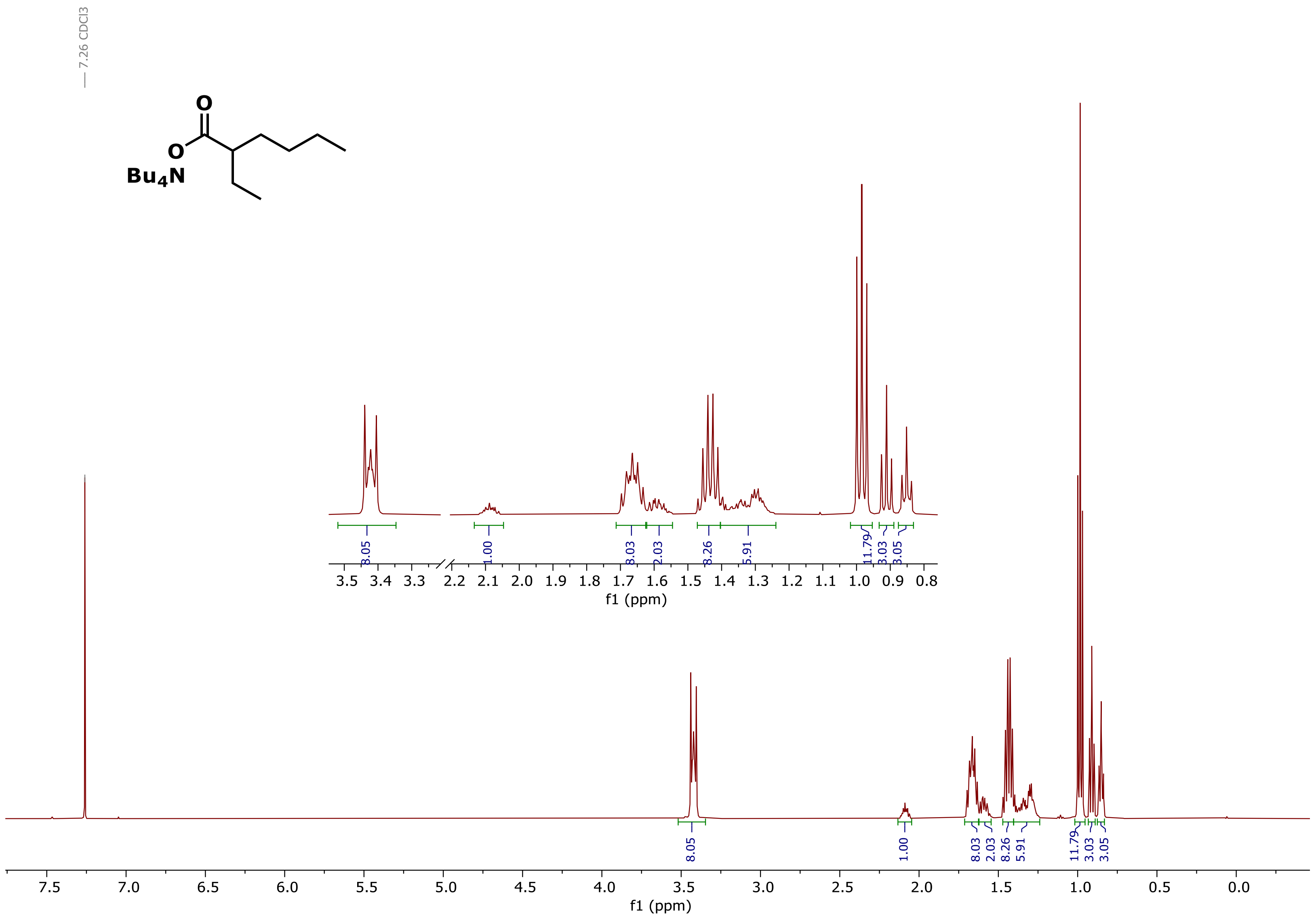

Figure S1. ^1H NMR (500 MHz, $CDCl_3$) tetrabutylammonium 2-ethylhexanoate

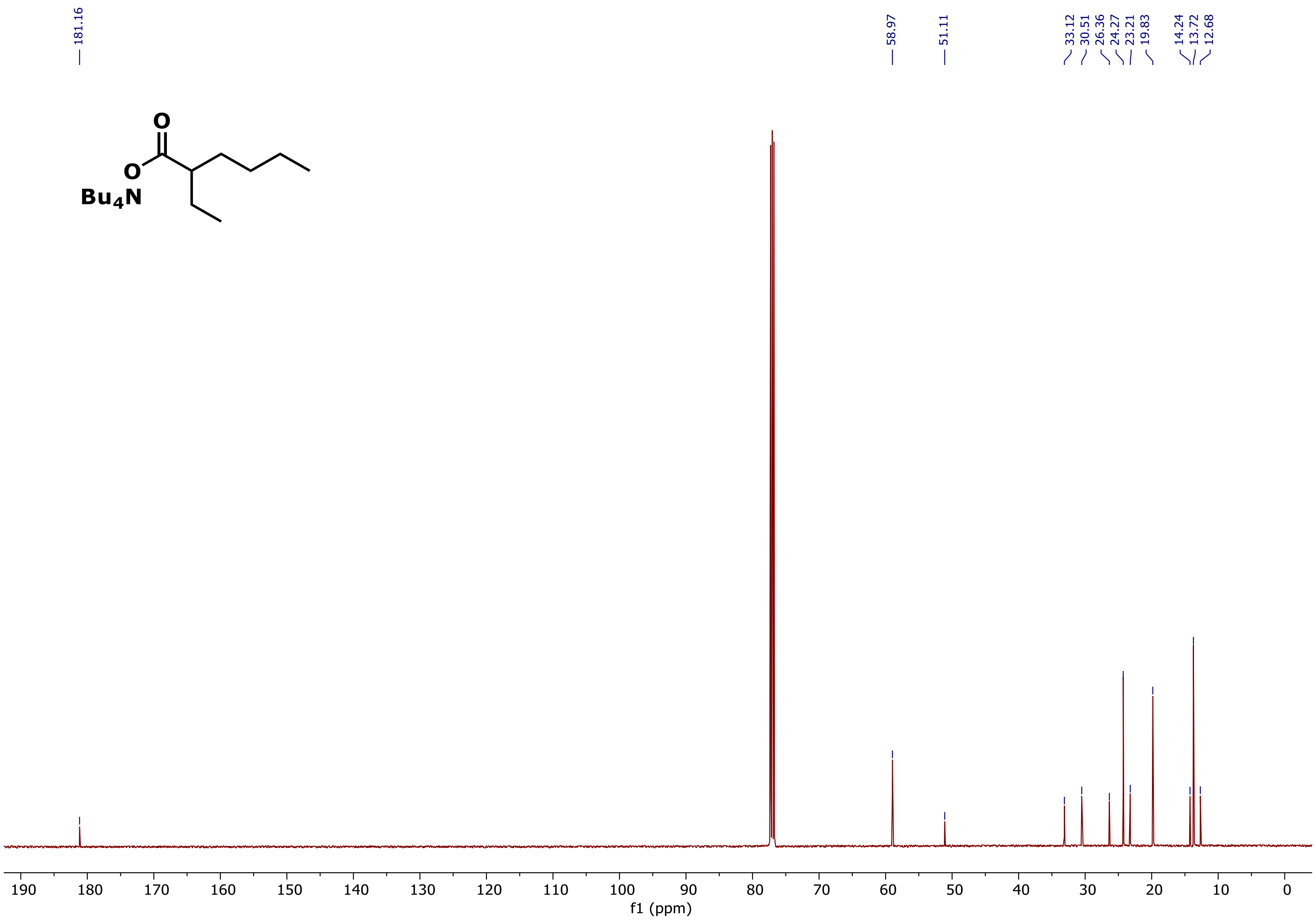

Figure S2. ^{13}C NMR (126 MHz, $CDCl_3$) tetrabutylammonium 2-ethylhexanoate

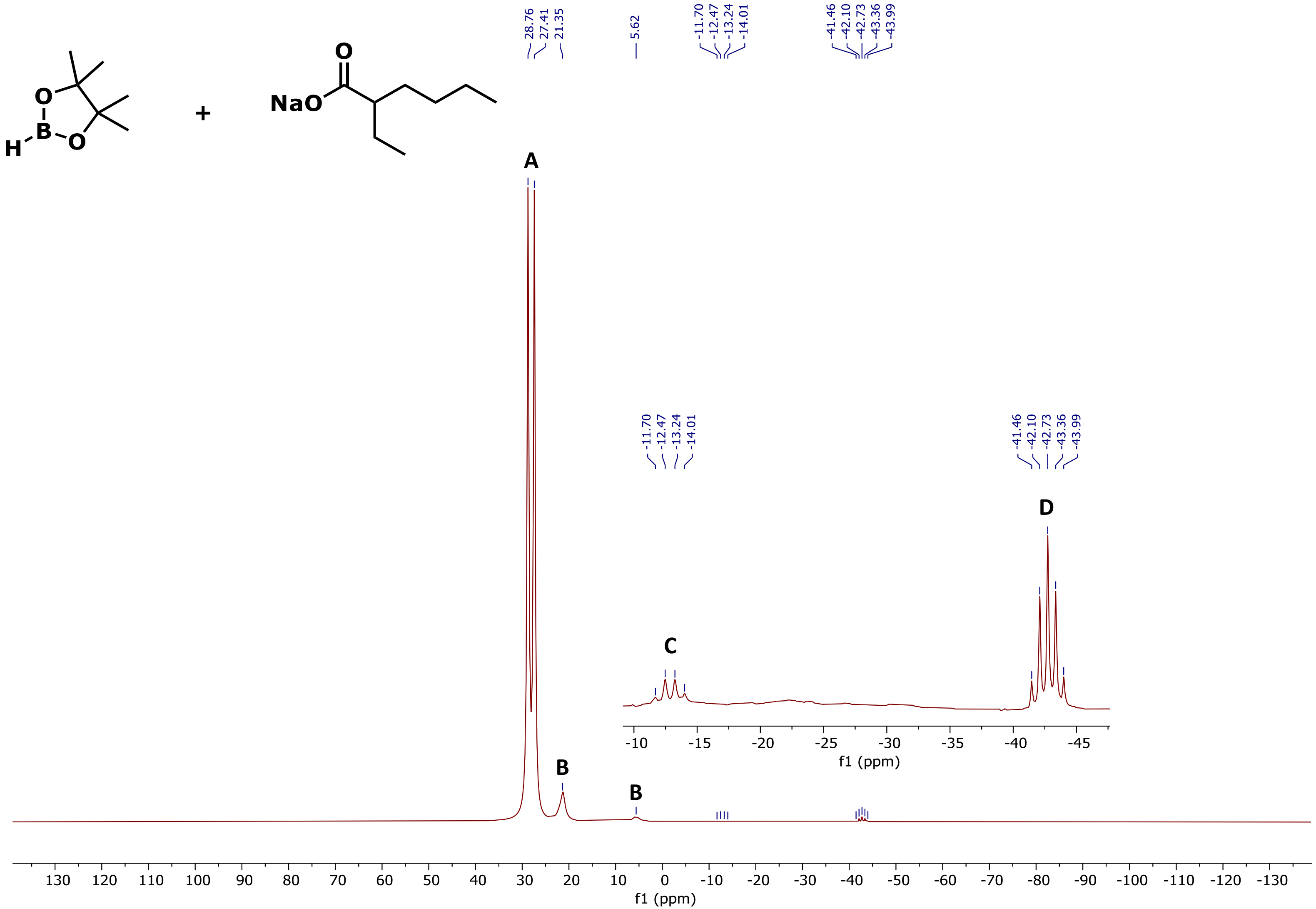

Figure S3. ^{11}B NMR (160 MHz, THF) Reaction of the activator, Na(2-EH), and HBpin after 24 h at 60 °C, ligand redistribution to a mixture of boron-containing species, including HBpin **A**, boron 'ate' complexes **B**, BH_3 **C** and BH_4^- **D**.

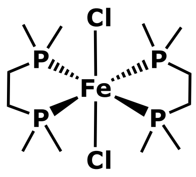

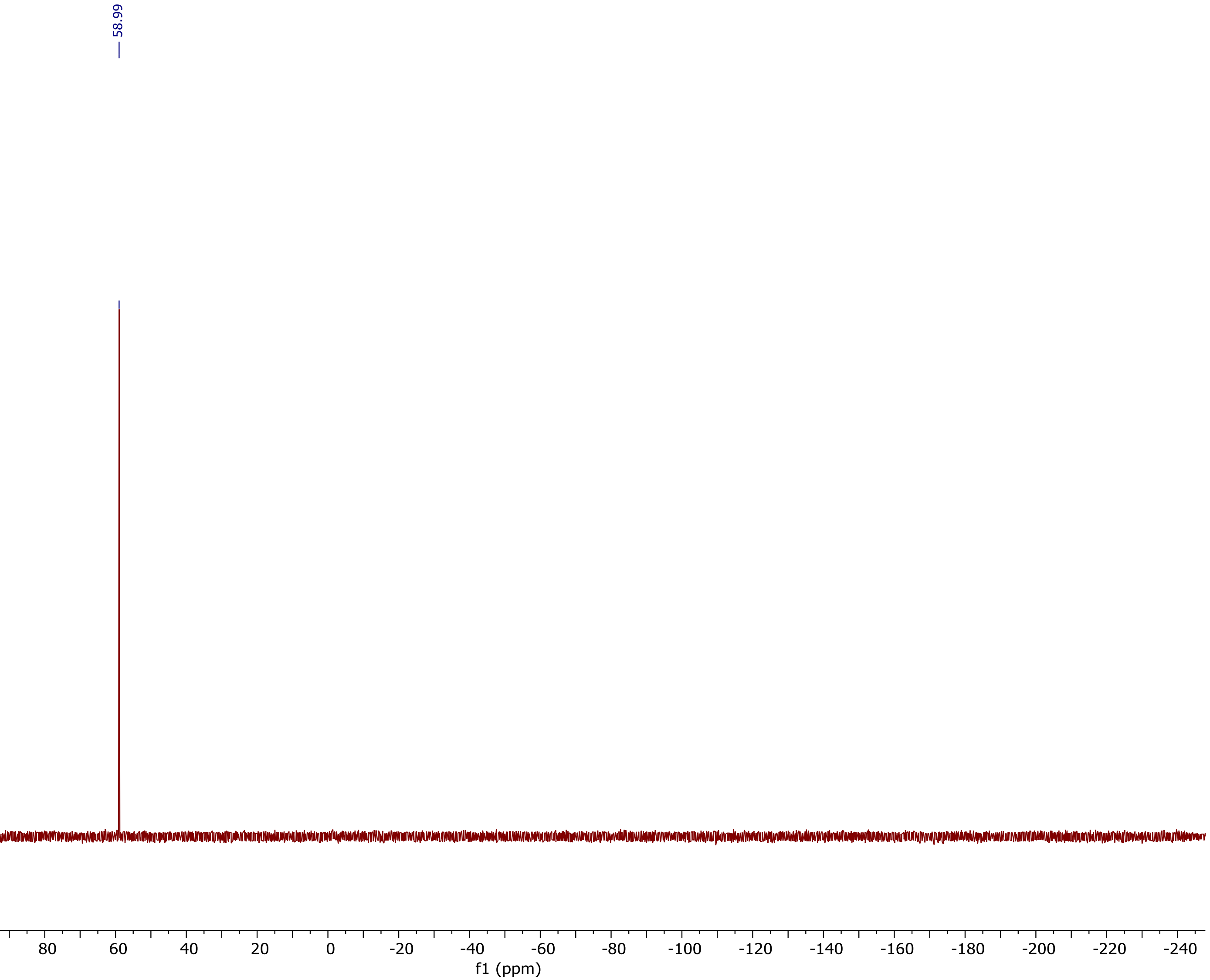

Figure S4. ^{31}P NMR (202 MHz, $CDCl_3$) $dmpe_2FeCl_2$ **1**

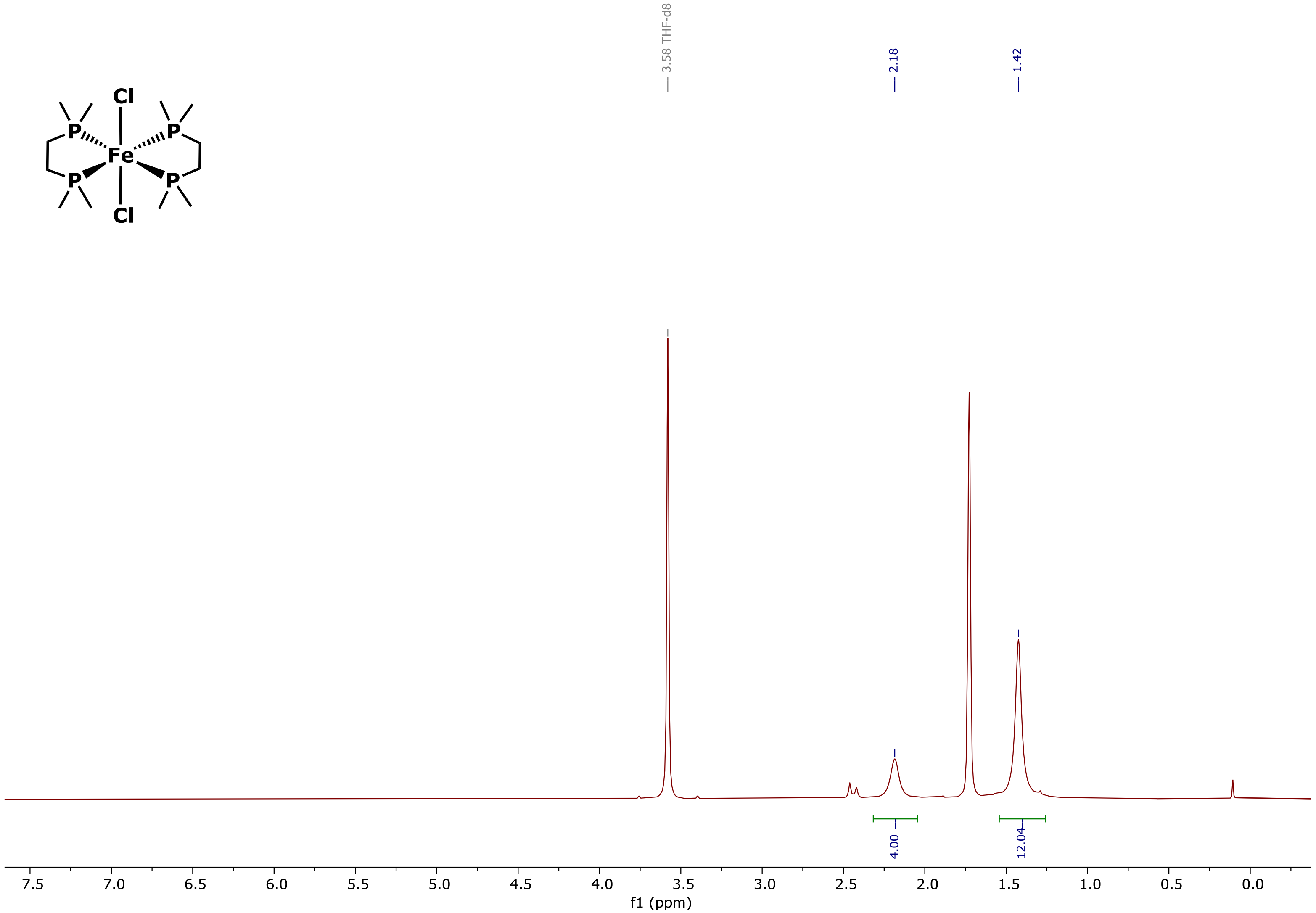

Figure S5. ^{1}H NMR (400 MHz, d_8-THF) $dmpe_2FeCl_2$ **1**

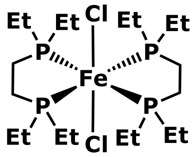

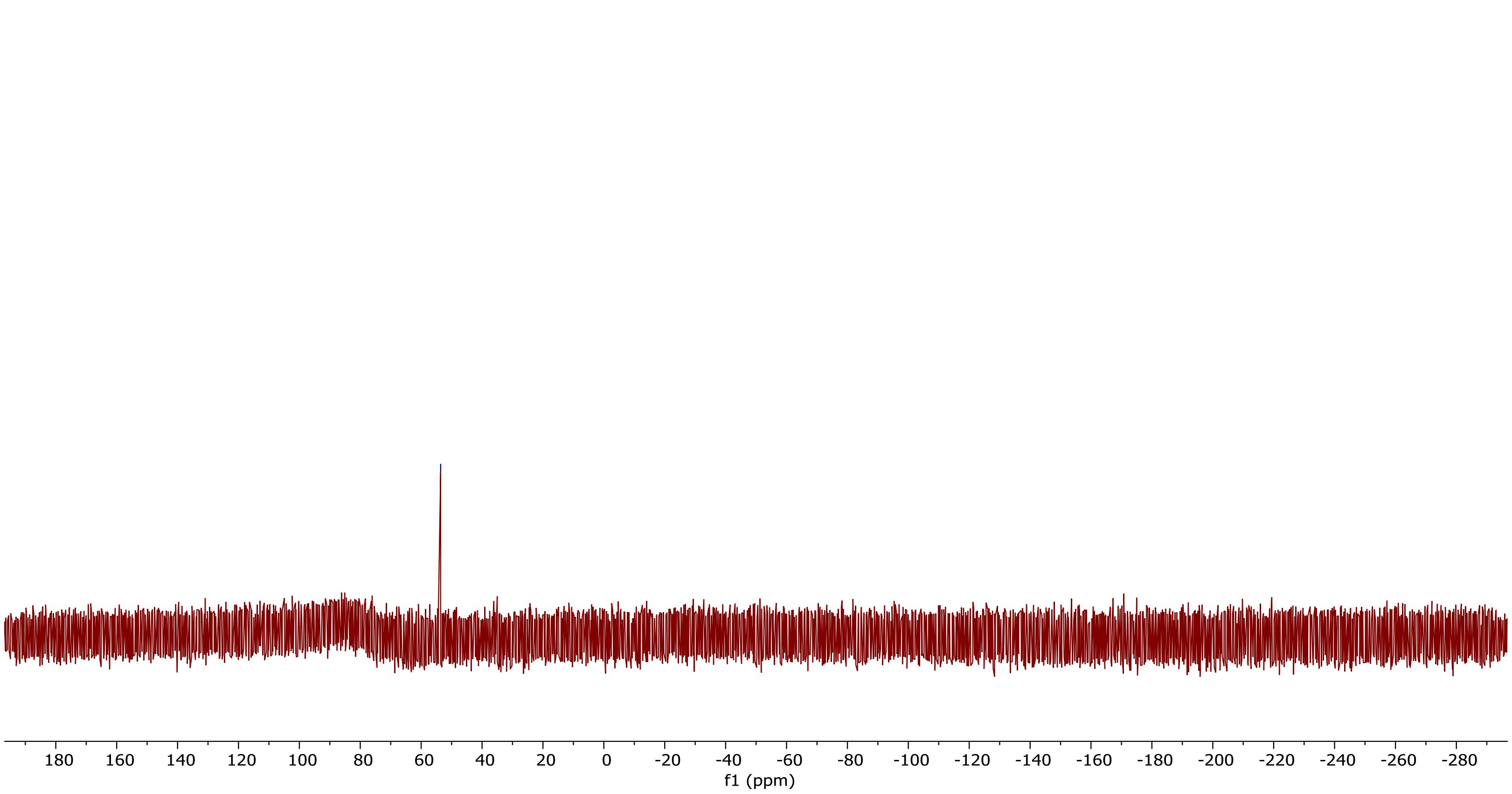

Figure S6. ^{31}P NMR (202 MHz, C_6D_6) $depe_2FeCl_2$ **1b**

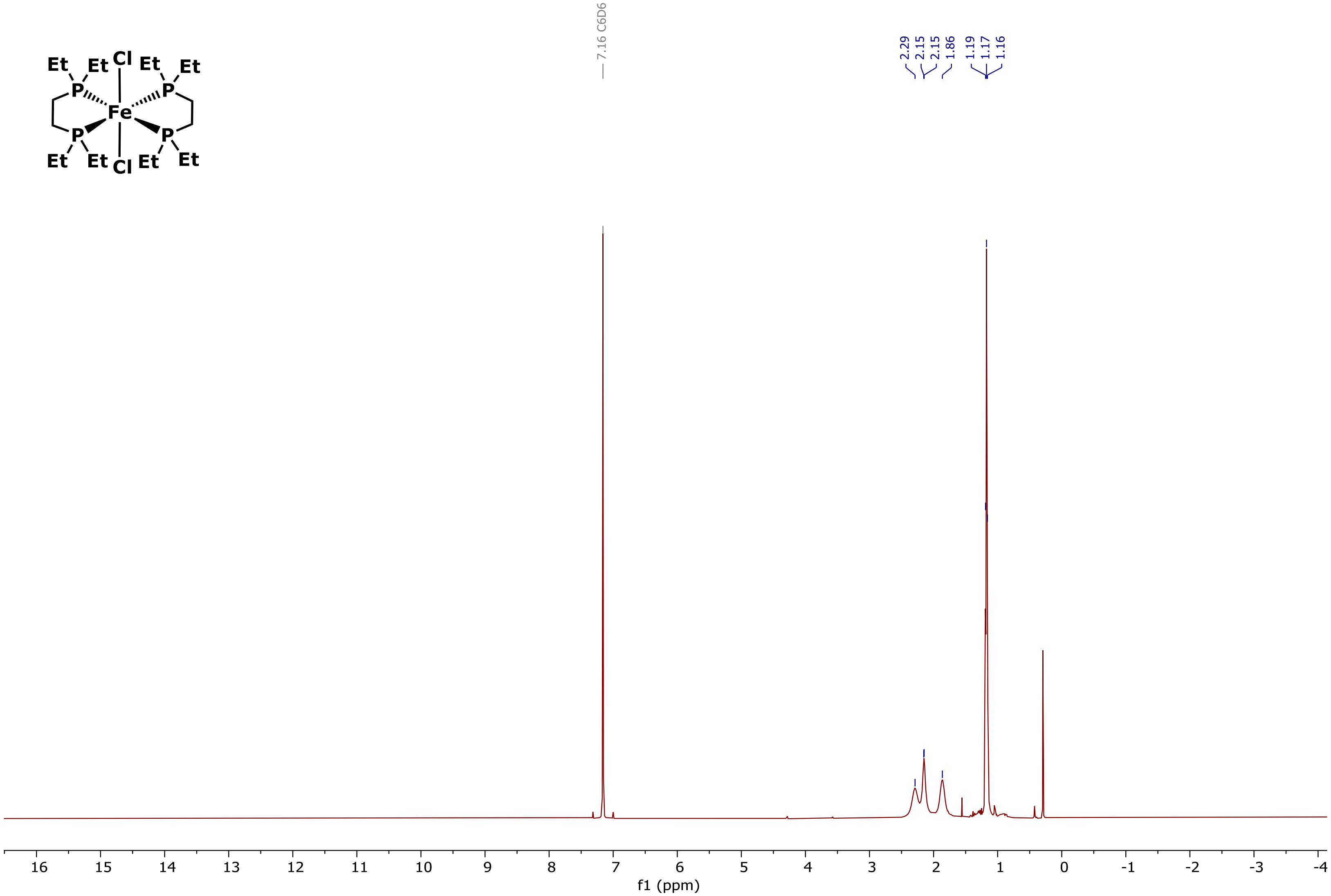

Figure S7. ^{1}H NMR (500 MHz, $CDCl_3$) $depe_2FeCl_2$ **1b**

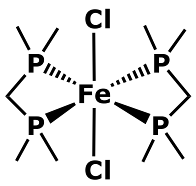

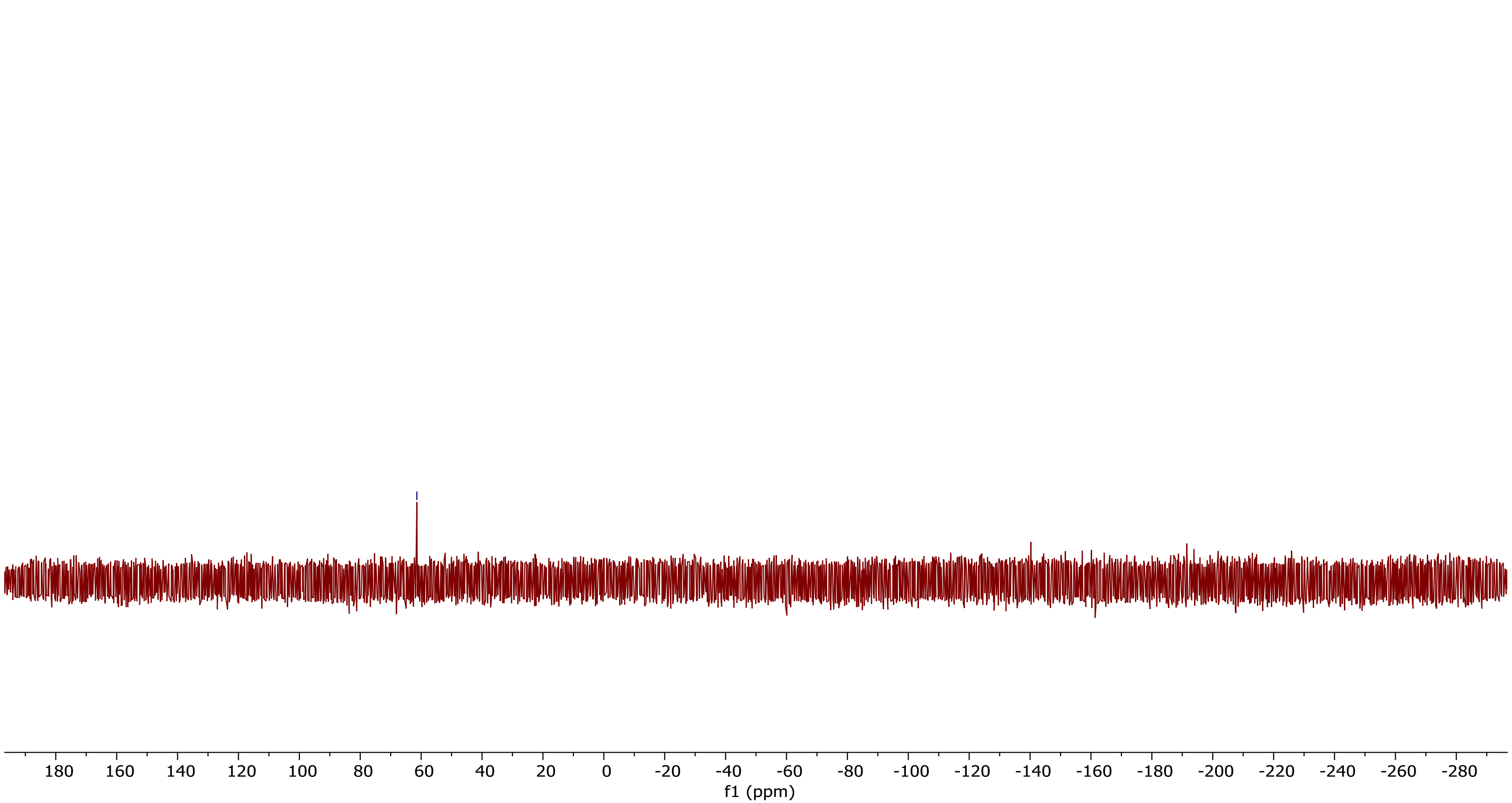

Figure S8. ^{31}P NMR (202 MHz, C_6D_6) $dmpm_2FeCl_2$ **1d**

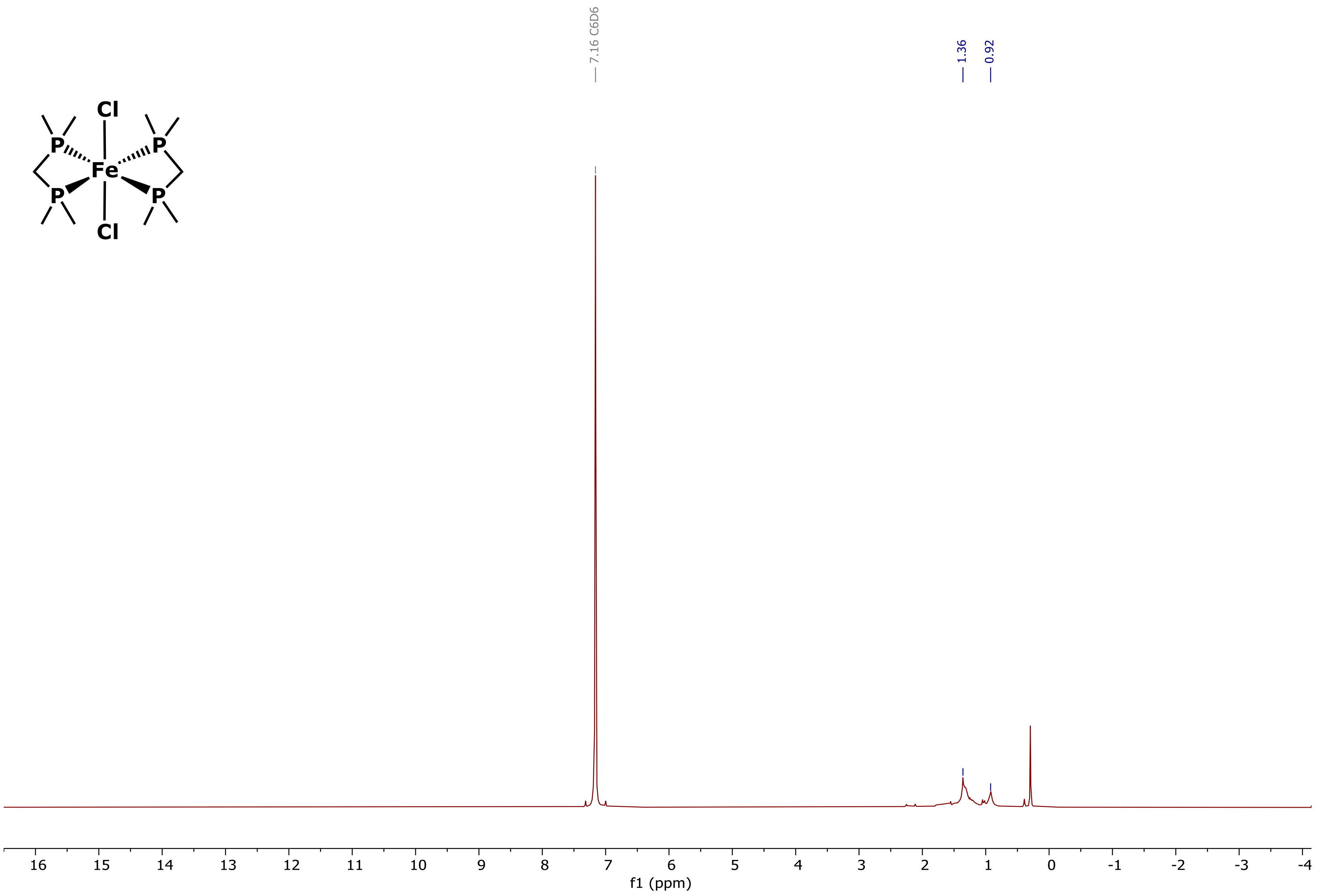

Figure S9. ^{1}H NMR (500 MHz, C_6D_6) $dmpm_2FeCl_2$ **1d**

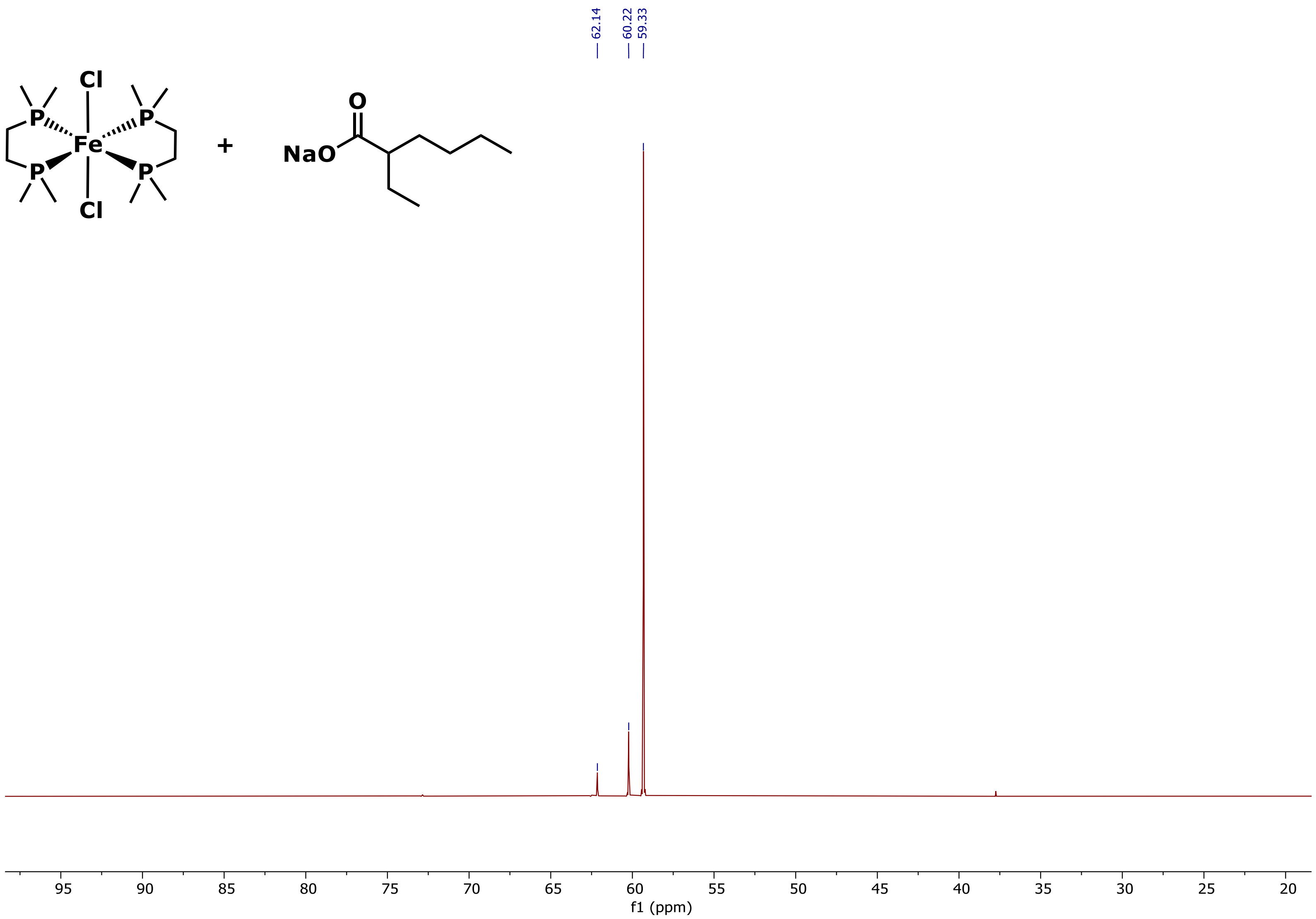

Figure S10. ^{31}P NMR (202 MHz, d_8-THF) $dmpe_2FeCl_2$ **1** + Na(2-EH) (2 equiv.) *r.t.* 30 minutes

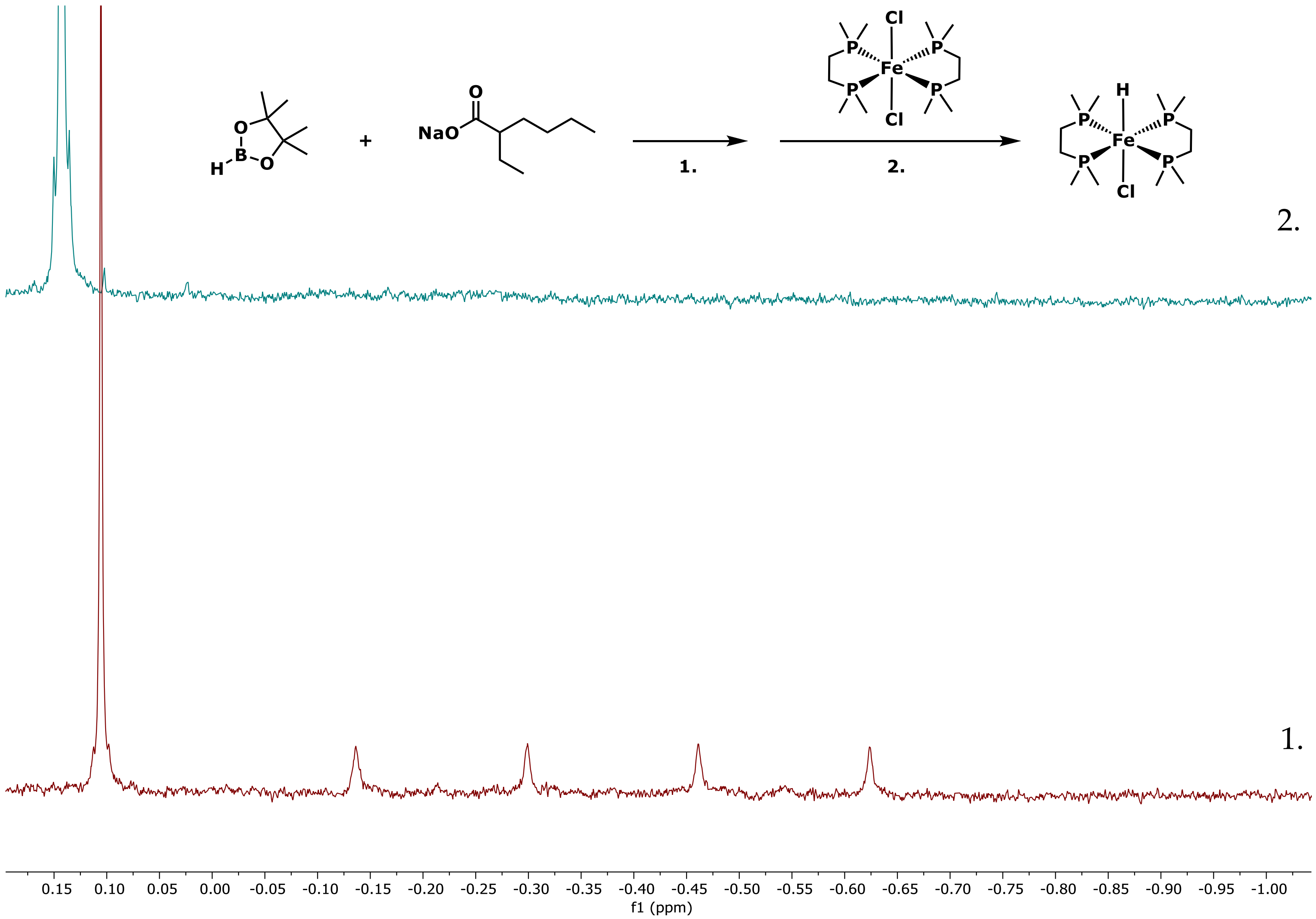

Figure S11. ^{1}H NMR (500 MHz, d_8-THF) 1: Na(2-EH) + HBpin (xs.) *r.t.* 30 minutes. 2: stoichiometric $dmpe_2FeCl_2$ **1** added after 1 day. Disappearance of BH_4 quartet

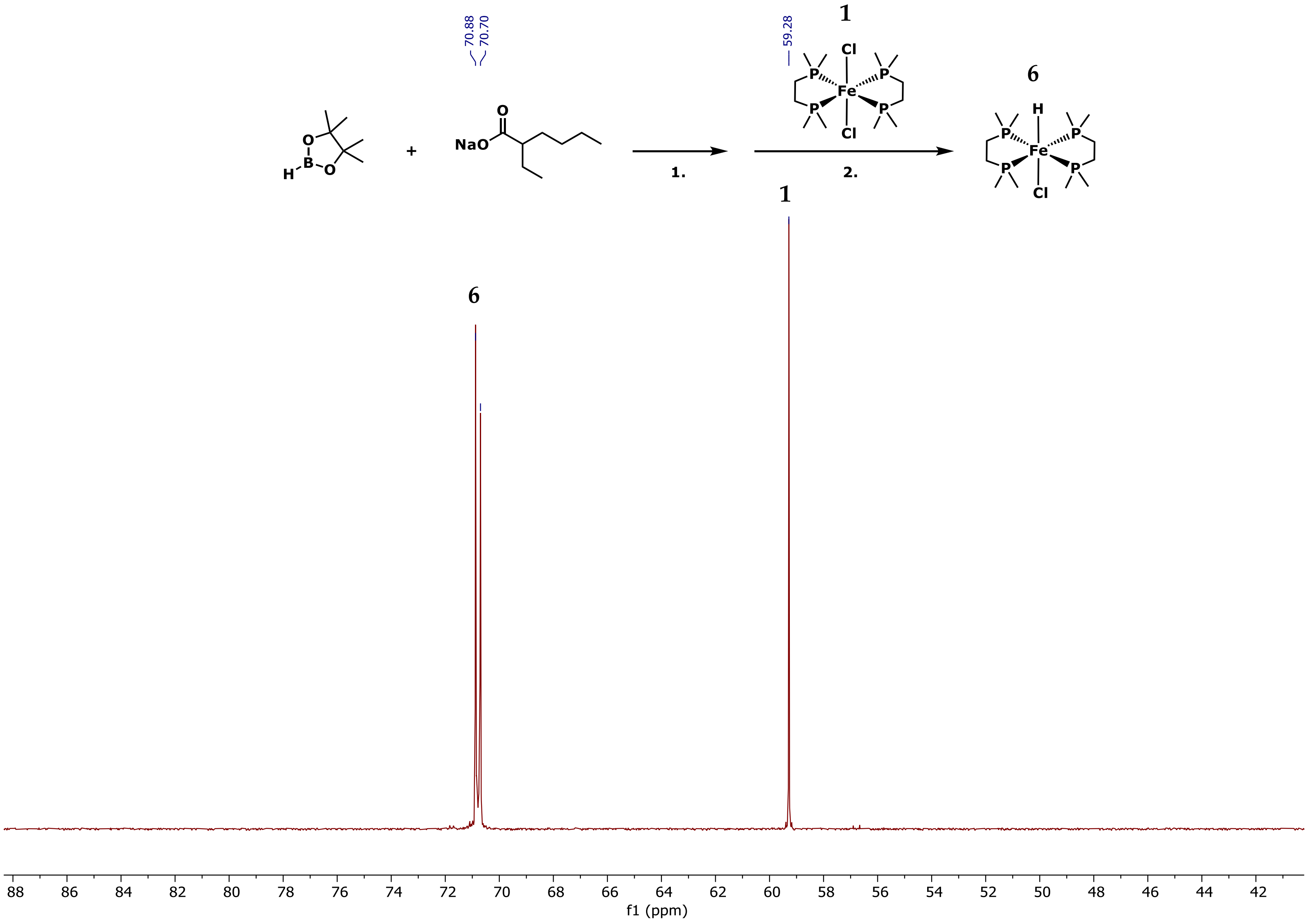

Figure S12. ^{31}P NMR (202 MHz, d_8-THF) 2: stoichiometric $dmpe_2FeCl_2$ **1** added after 1 day. Appearance of $dmpe_2FeHCl$ **6**.

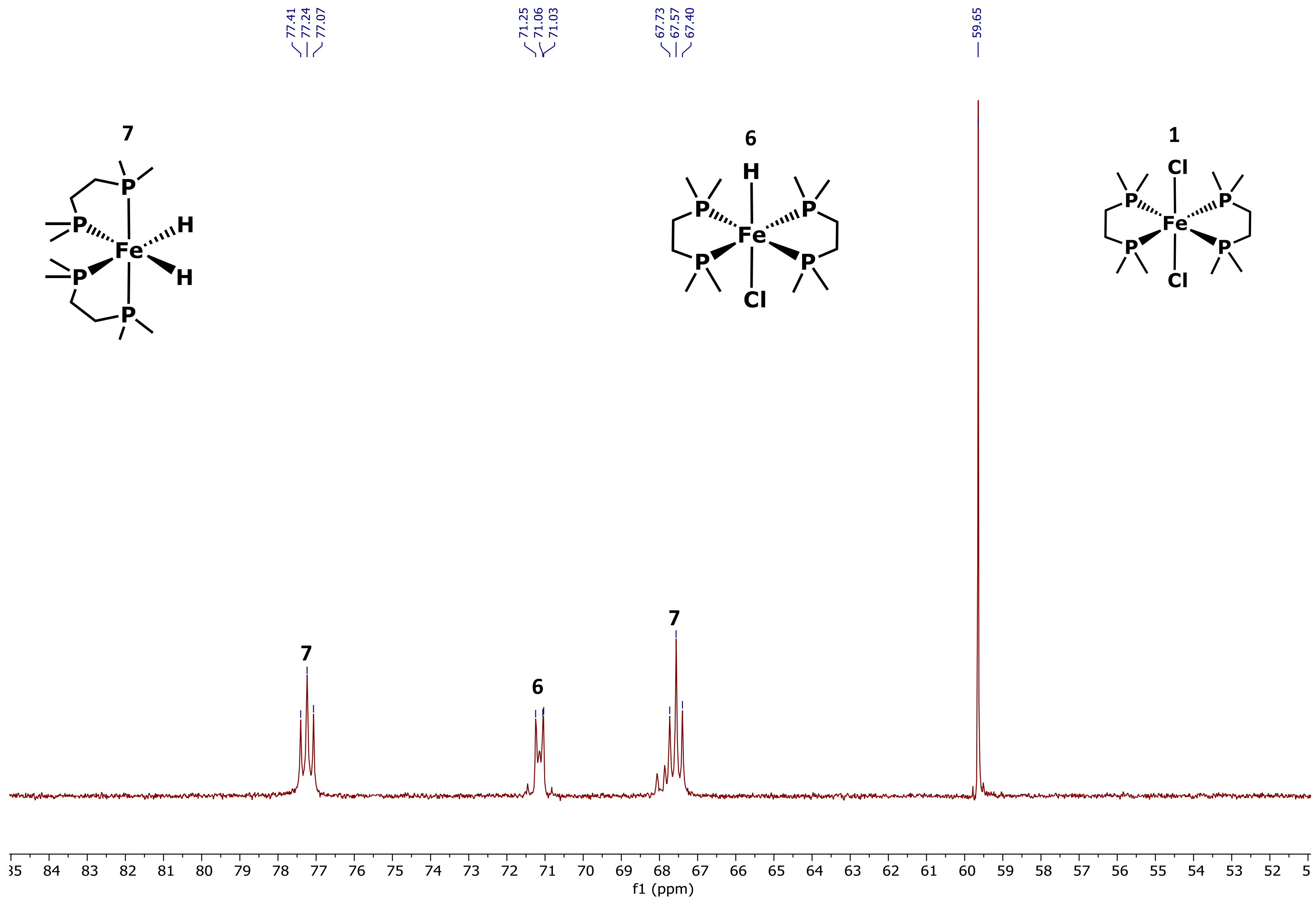

Figure S13. ^{31}P NMR (202 MHz, THF) $dmpe_2FeH_2$ **7** and dmpe₂FeHCl **6**. $dmpe_2FeCl_2$ **1** + HBpin (2 equiv.) + Na(2-EH) (2 equiv.) + THF at 60 °C for 6 h.

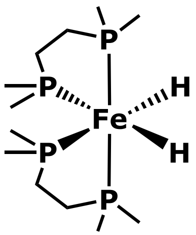

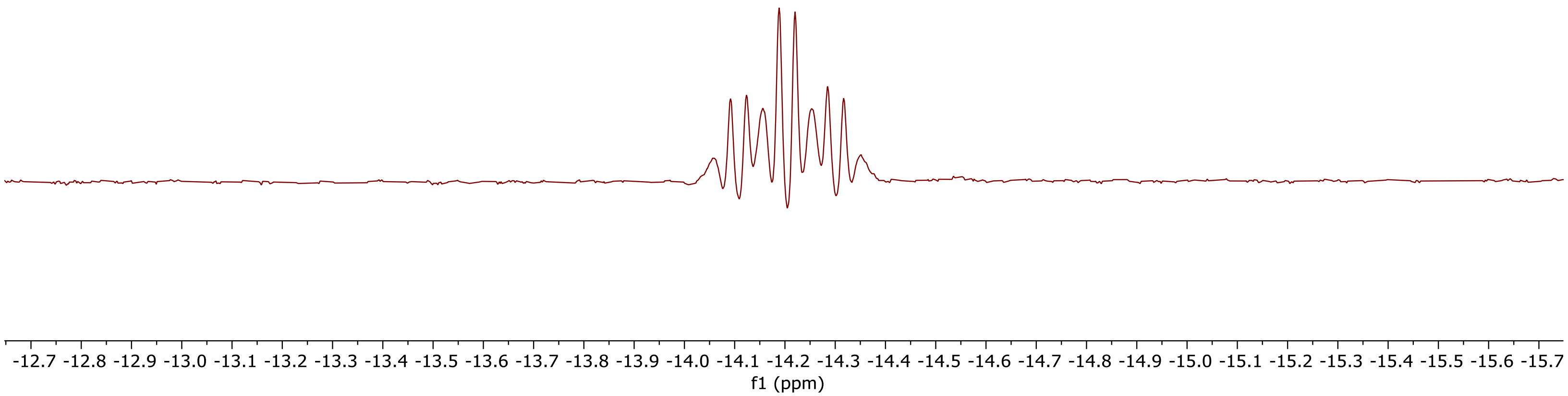

Figure S14. ^{1}H NMR (600 MHz, THF) $dmpe_2FeH_2$ **7.** $dmpe_2FeCl_2$ **1** + HBpin (2 equiv.) + Na(2-EH) (4 equiv.) + THF at 60 °C for 24 h.

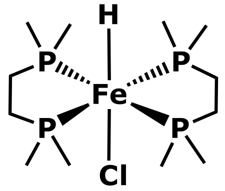

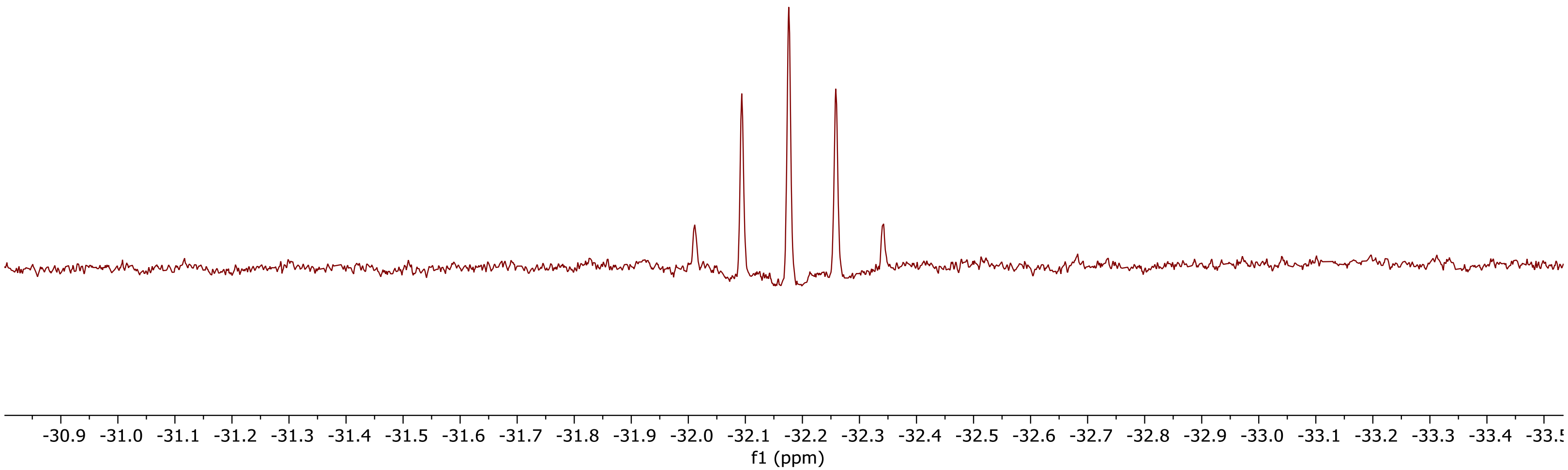

Figure S15. ^{1}H NMR (600 MHz, THF) $dmpe_2FeHCl$ **6.** $dmpe_2FeCl_2$ **1** + HBpin (2 equiv.) + Na(2-EH) (4 equiv.) + THF at 60 °C for 24 h.

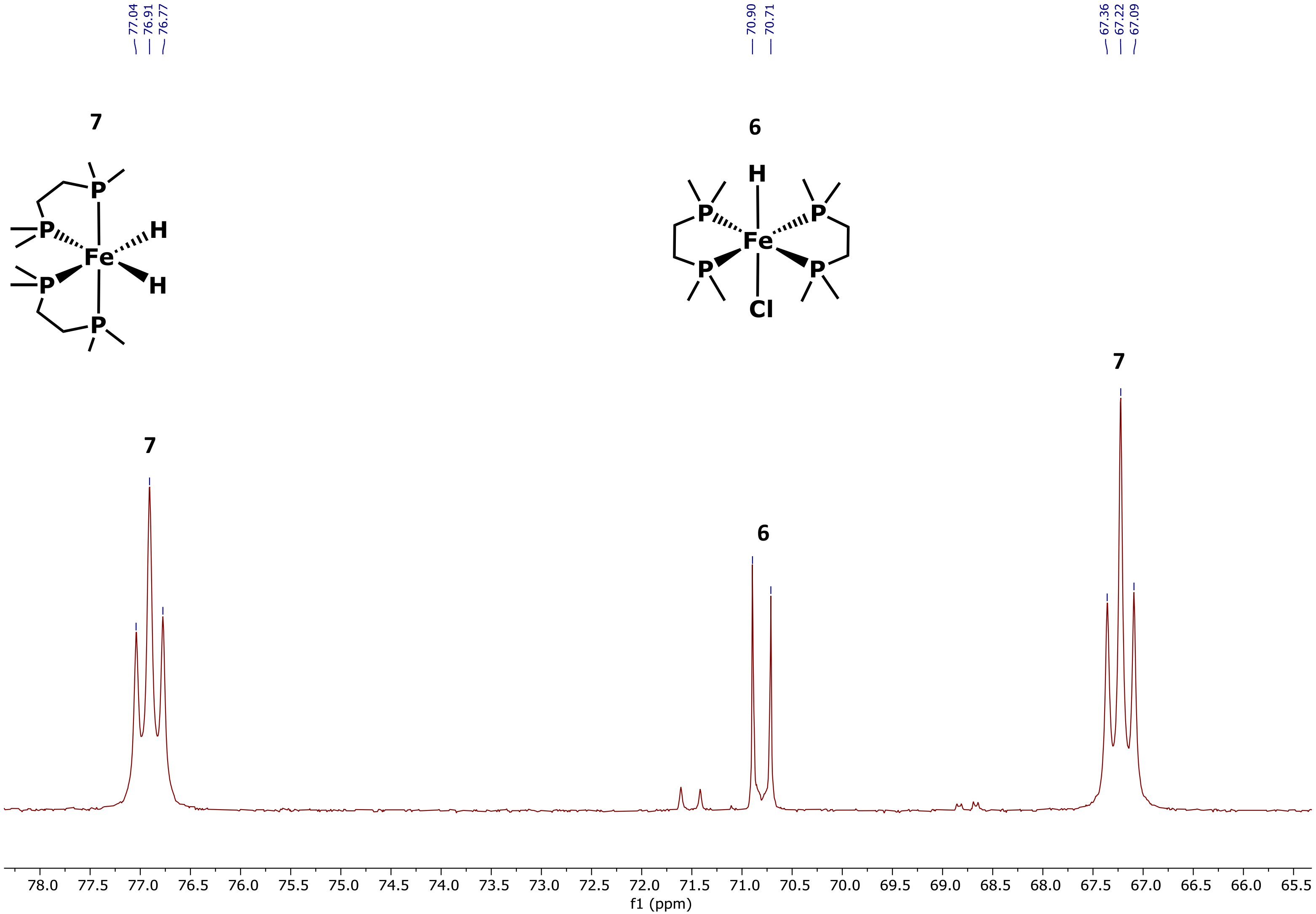

Figure S16. ^{31}P NMR (202 MHz, THF) $dmpe_2FeH_2$ **7** and $dmpe_2FeHCl$ **6**. $dmpe_2FeCl_2$ **1** + HBpin (2 equiv.) + Na(2-EH) (4 equiv.) + THF at 60 °C for 24 h.

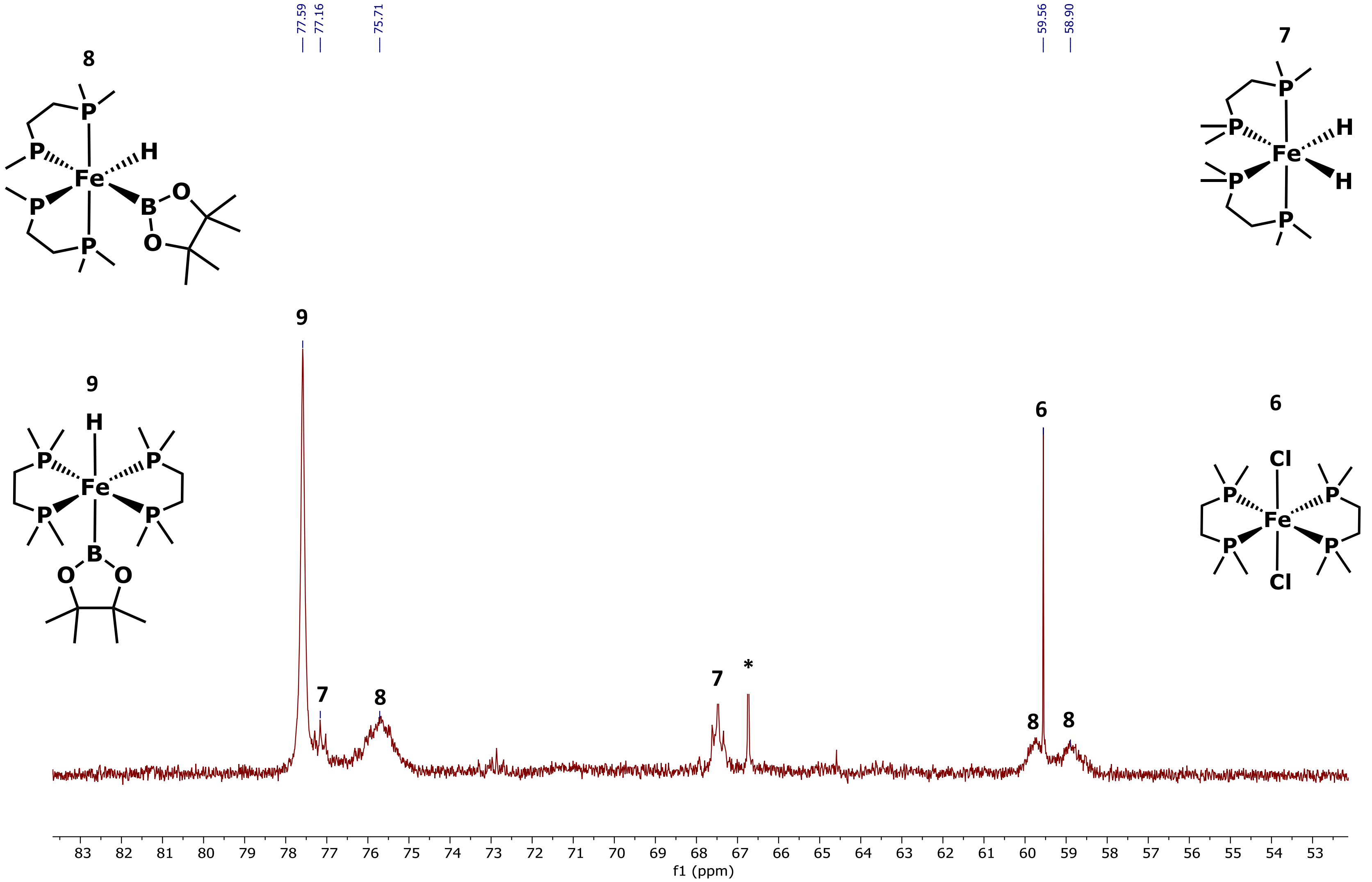

Figure S17. ^{31}P NMR (202 MHz, THF) *cis*-dmpe$_2$FeH(Bpin) **8**, *trans*-dmpe$_2$FeH(Bpin) **9,** dmpe$_2$FeH$_2$ **7**, dmpe$_2$FeCl$_2$ **6**, and unknown *****. dmpe$_2$FeCl$_2$ **1** + HBpin (xs.) + Na(2-EH) (2 equiv.) + THF + blue light for 16 h.

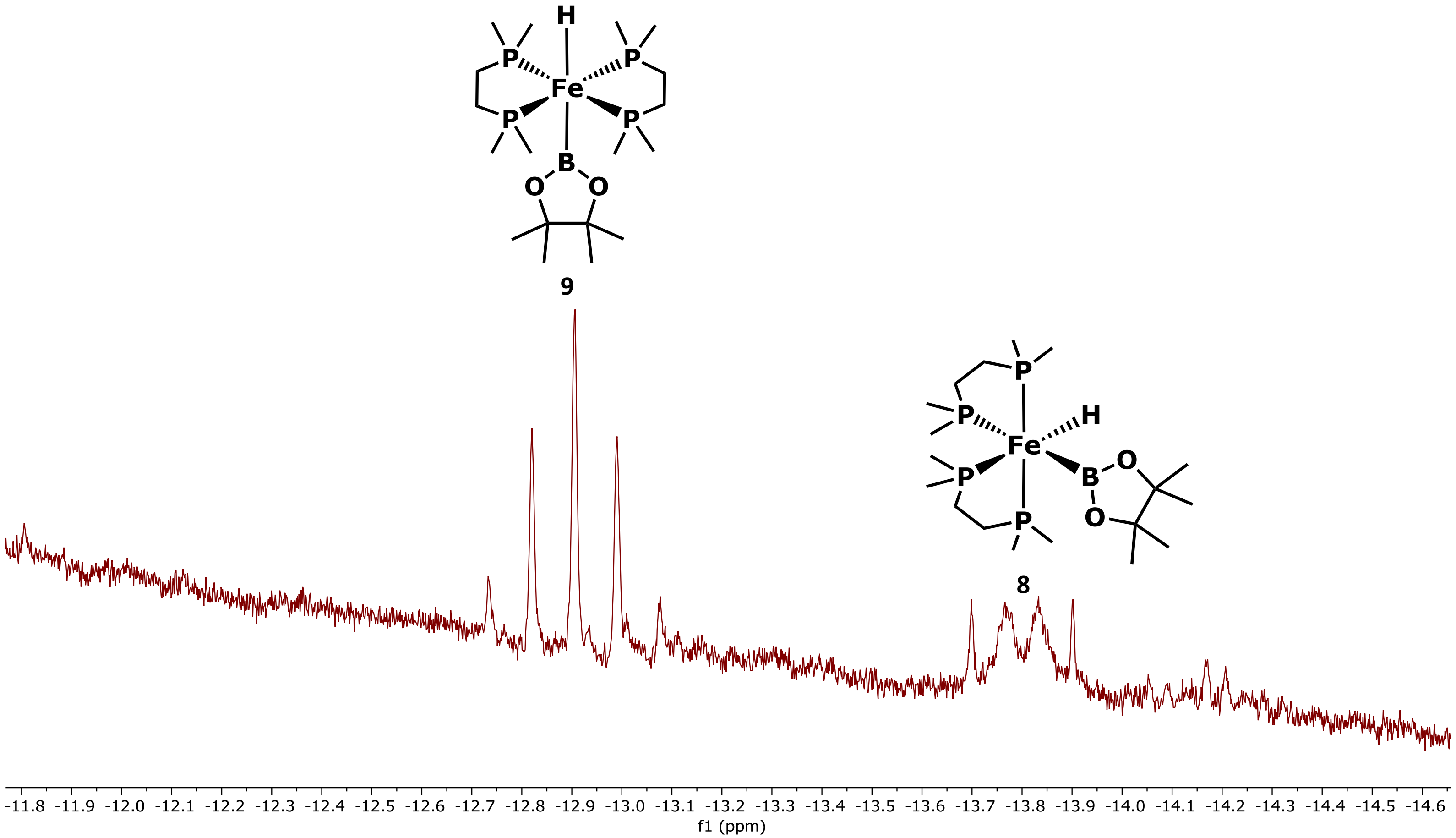

Figure S18. ^{1}H NMR (500 MHz, THF) *cis*-dmpe$_2$FeH(Bpin) **8** and *trans*-dmpe$_2$FeH(Bpin) **9.** dmpe$_2$FeCl$_2$ **1** + HBpin (xs.) + Na(2-EH) (2 equiv.) + THF + blue light for 16 h.

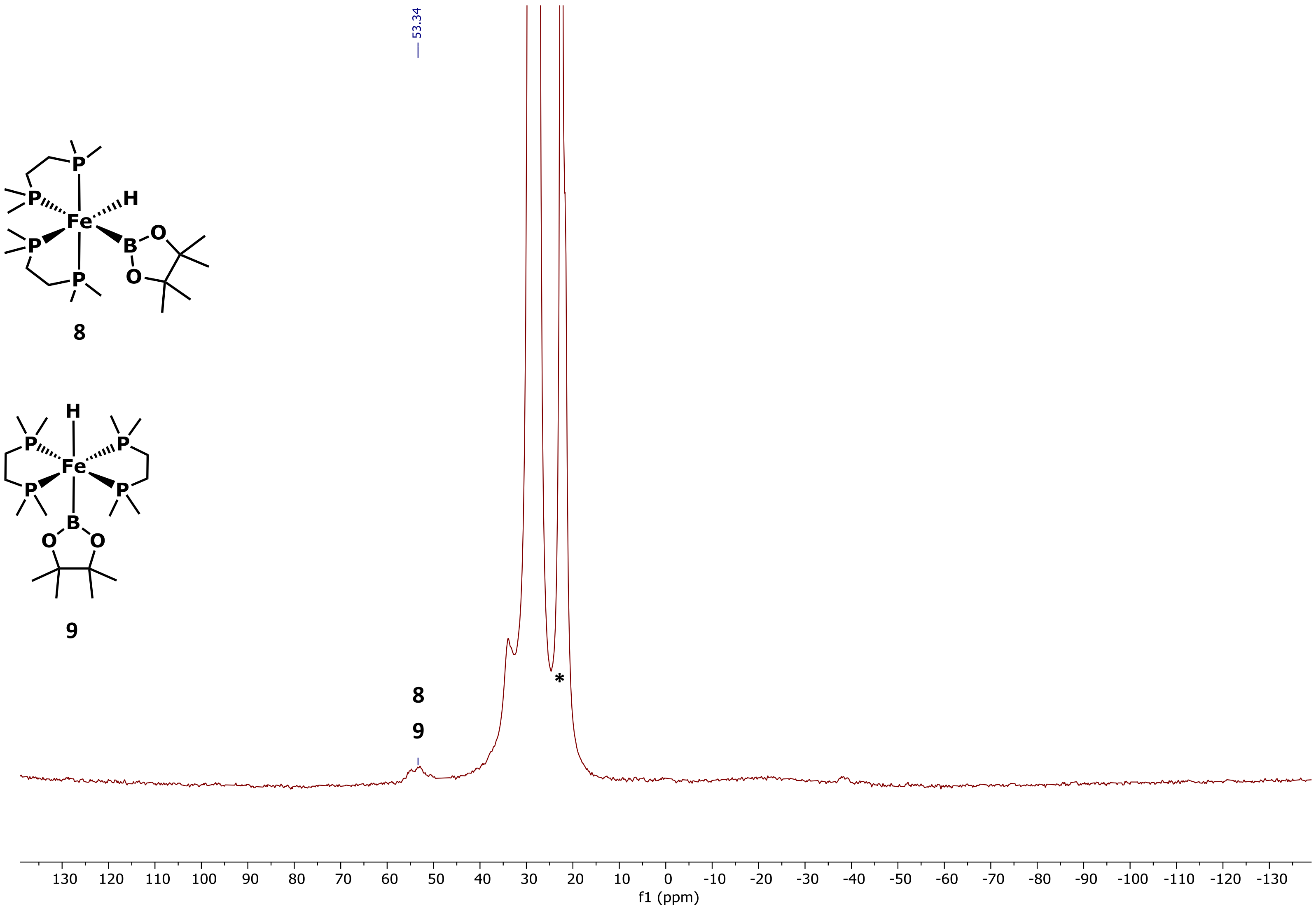

Figure S19. ^{11}B NMR (160 MHz, THF) $dmpe_2FeH(Bpin)$ **8** and **9** and *: $B(OR)_3$ impurity. $dmpe_2FeCl_2$ **1** + HBpin (xs.) + Na(2-EH) (2 equiv.) + THF + blue light for 16 h.

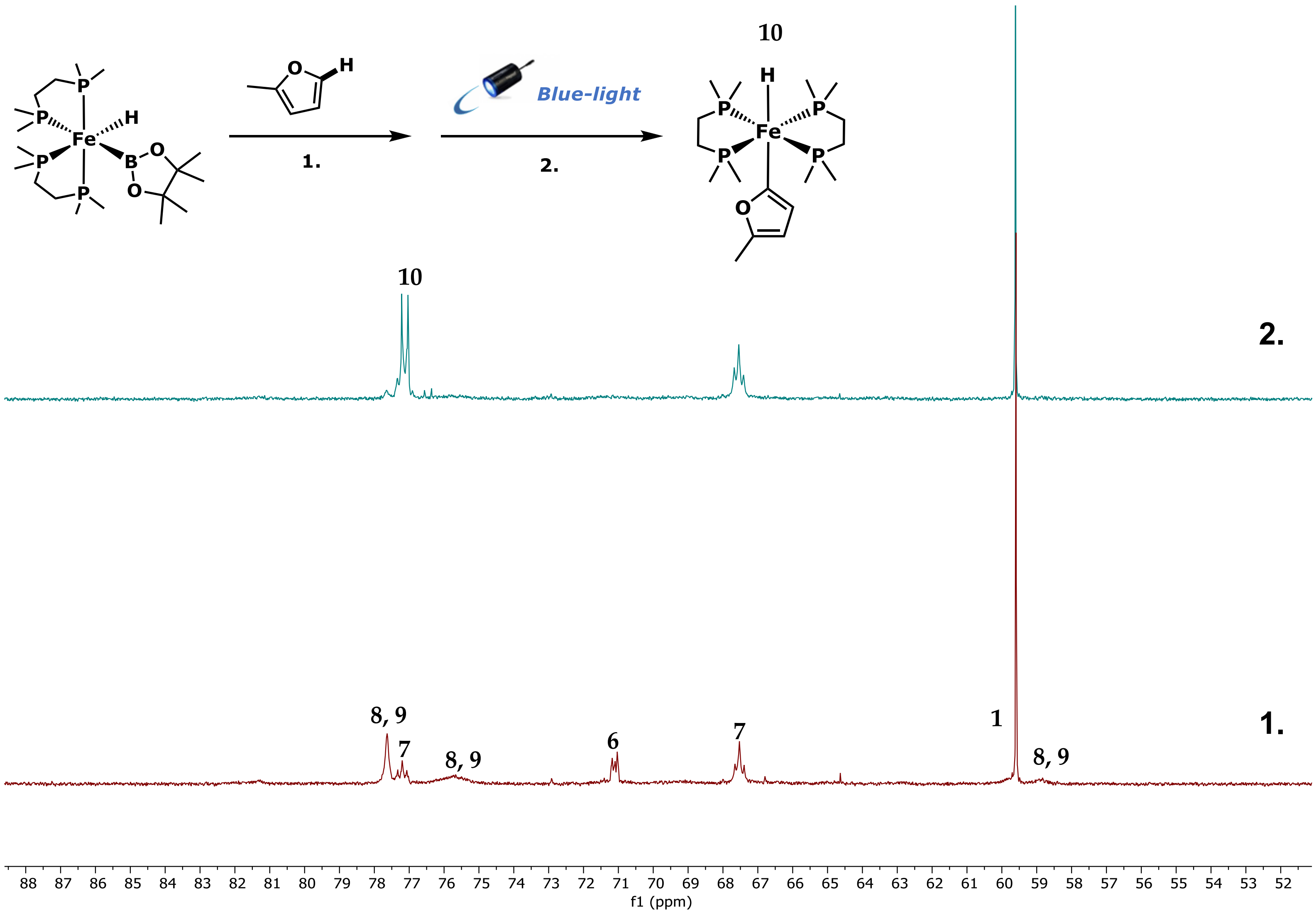

Figure S20. ^{31}P NMR (202 MHz, THF) 1: Solution containing dmpe$_2$FeH(Bpin) **8** and **9**, dmpe$_2$FeH$_2$ **7**, dmpe$_2$FeCl$_2$ **1**, dmpe$_2$FeHCl **6**, and 2-methylfuran (xs.) 20 h at 60 °C. 2: After blue light irradiation for 3 h, development of dmpe$_2$FeH(2-Me-furyl) **10** complex.

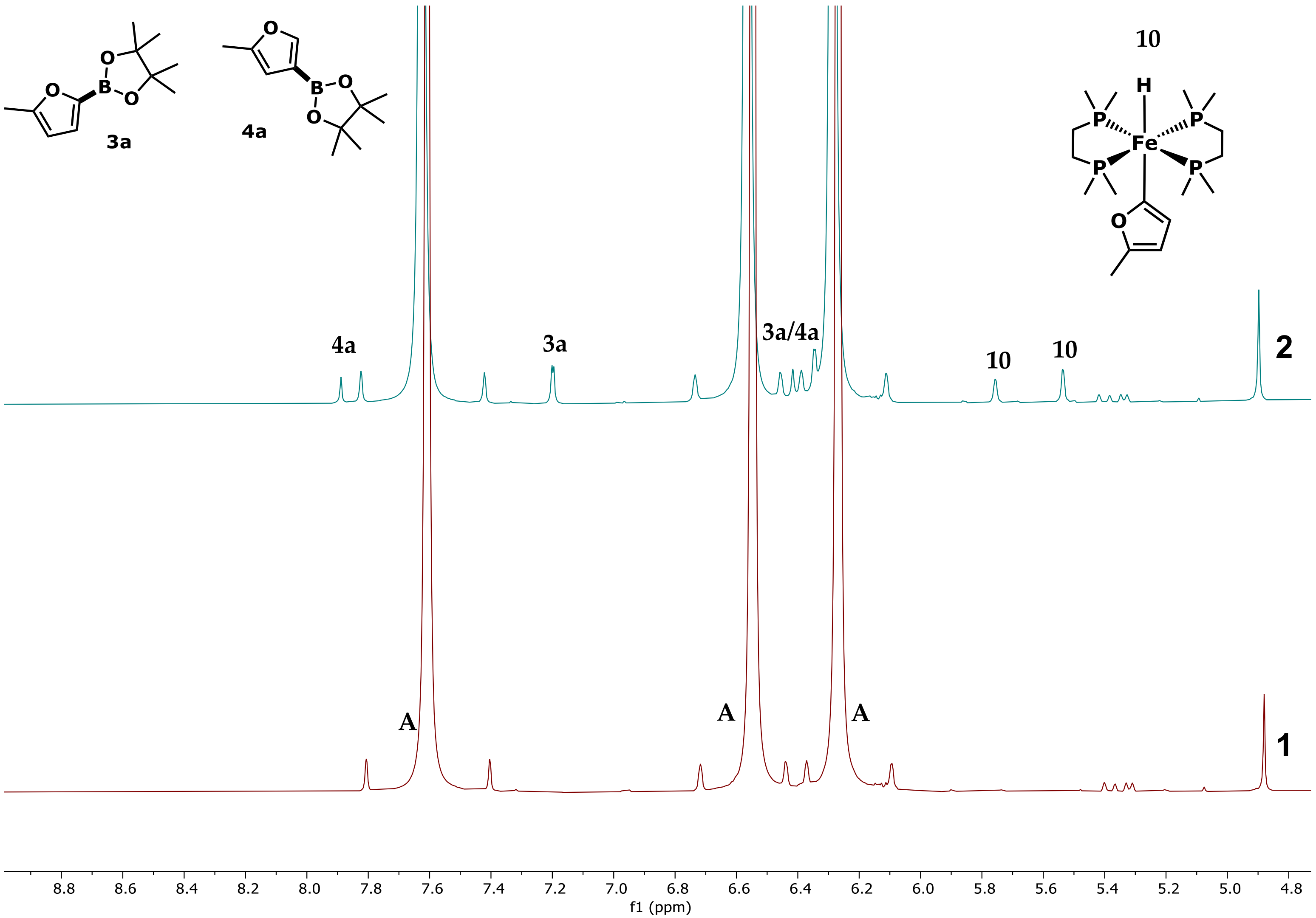

Figure S21. ^{1}H NMR (500 MHz, THF) 1: Solution containing dmpe₂FeH(BPin) **8** and **9**, dmpe₂FeH₂ **7**, dmpe₂FeCl₂ **1**, dmpe₂FeHCl, and 2-methylfuran (xs.) **A**, 20 h at 60 °C. 2: After blue light irradiation for 3 h, development of dmpe₂FeH(2-Me-furyl) complex **10**, and borylated 2-methylfuran **3a/4a**.

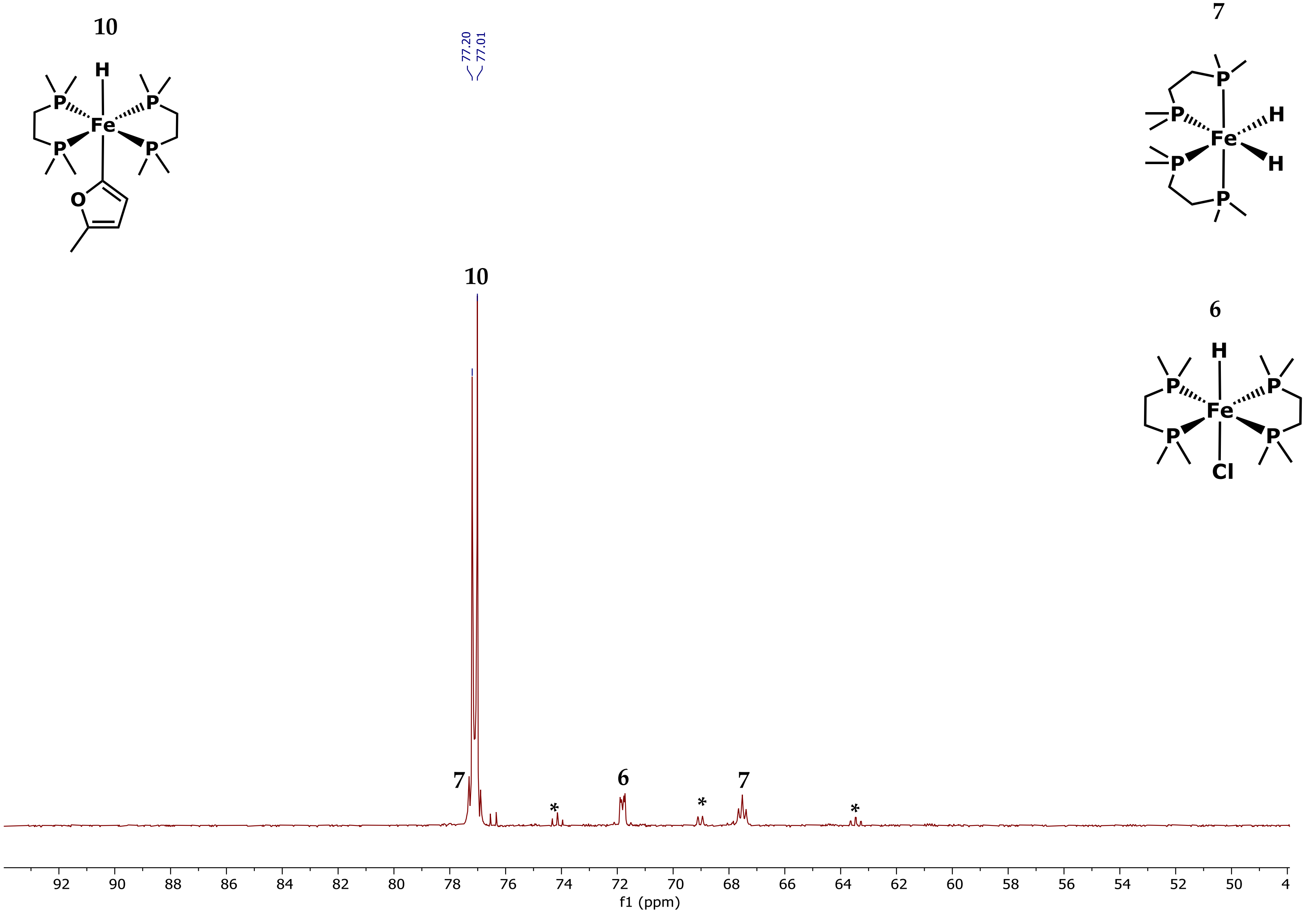

Figure S22. ^{31}P NMR (202 MHz, THF) dmpe$_2$FeH(2-Me-furyl) **10**, dmpe$_2$FeH$_2$ **7**, dmpe$_2$FeHCl **6**, and *: unknown. dmpe$_2$FeCl$_2$ **1** + HBpin (2 equiv.) + Na(2-EH) (2 equiv.) + THF + blue light for 24 h.

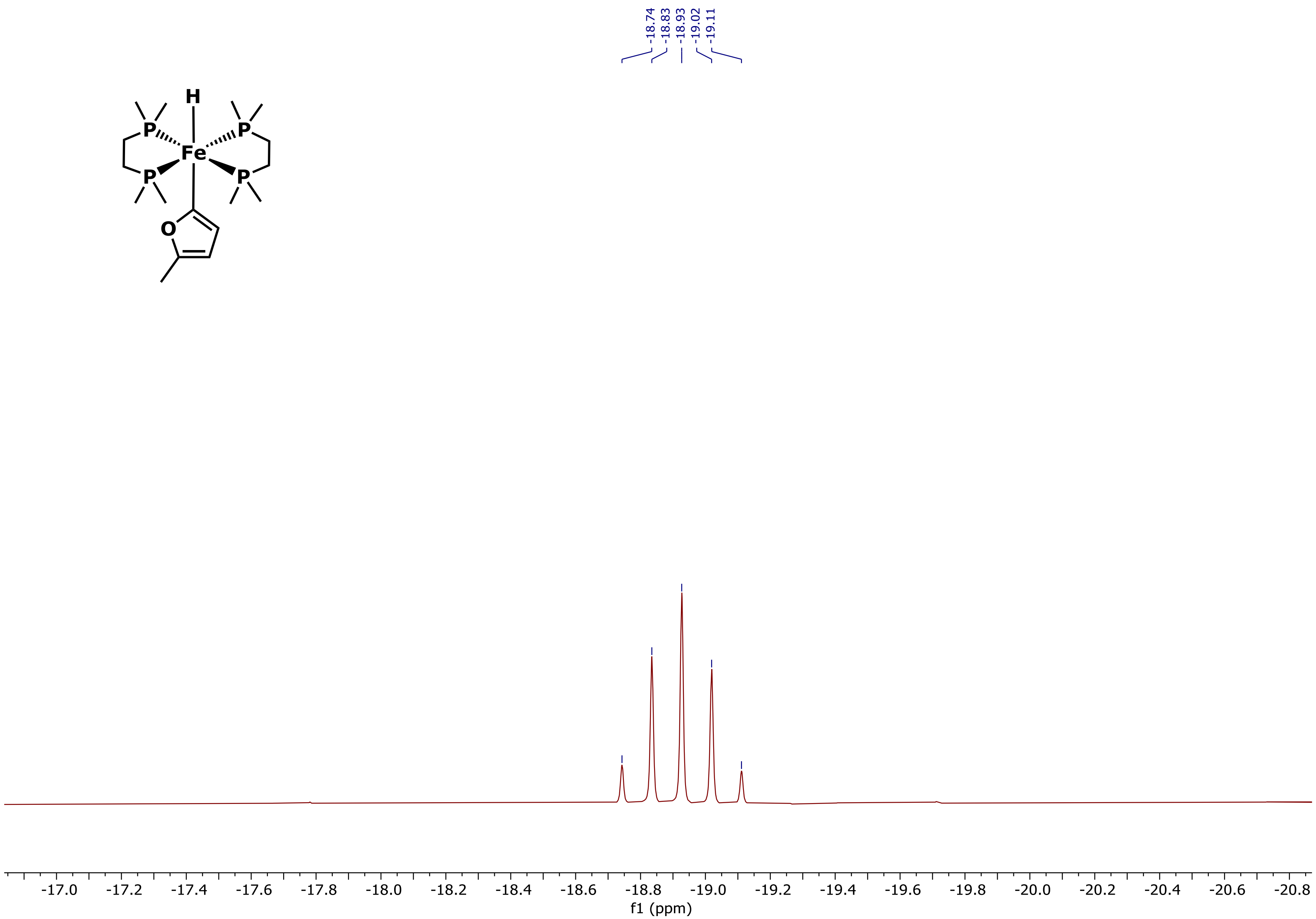

Figure S23. ^{1}H NMR (500 MHz, THF) $dmpe_2FeH$(2-Me-furyl) **10.** $dmpe_2FeCl_2$ **1** + HBpin (2 equiv.) + Na(2-EH) (2 equiv.) + THF + blue light for 24 h.

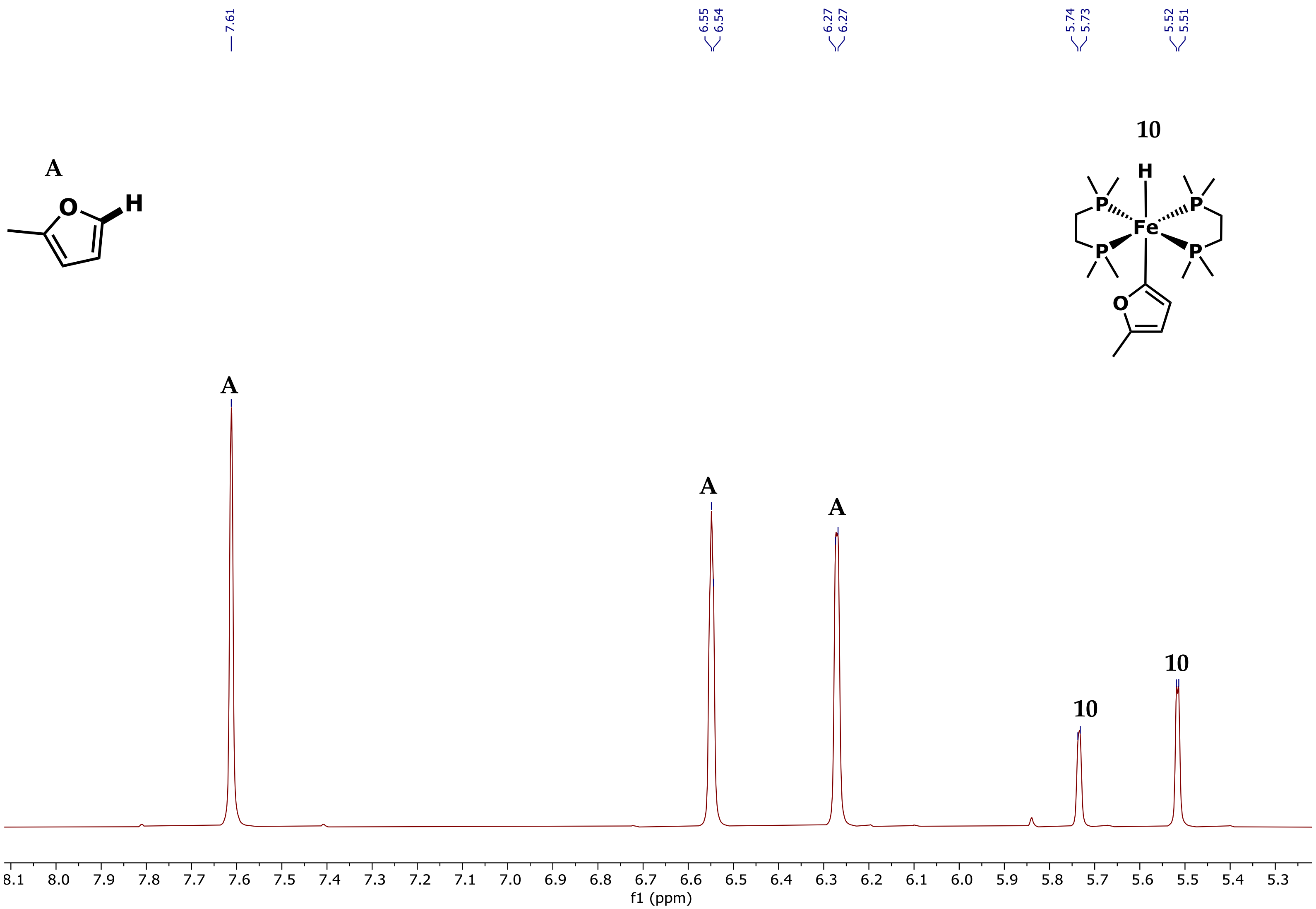

Figure S24. ^{1}H NMR (500 MHz, THF) dmpe$_2$FeH(2-Me-furyl) **10** and 2-methylfuran **A.** dmpe$_2$FeCl$_2$ **1** + HBpin (2 equiv.) + Na(2-EH) (2 equiv.) + THF + blue light for 24 h.

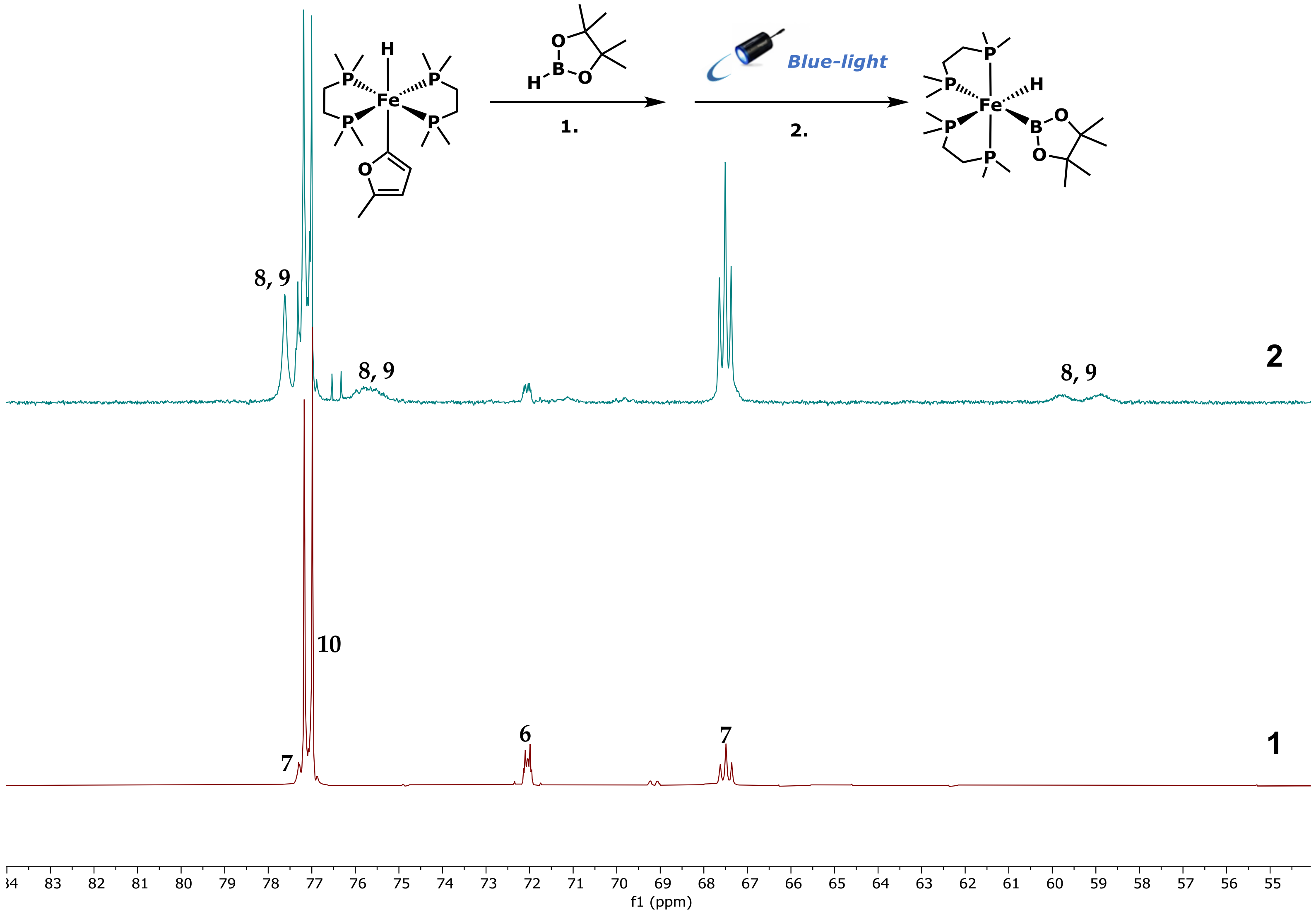

Figure S25. ^{31}P NMR (202 MHz, THF) **1**: Solution containing dmpe$_2$FeH(2-Me-furyl) **10**, dmpe$_2$FeH$_2$ **7**, dmpe$_2$FeCl$_2$ **6**, and HBpin (xs.), 20 h at 60 °C. **2**: After blue light irradiation for 3 h, formation of dmpe$_2$FeH(Bpin) complexes **8** and **9**.

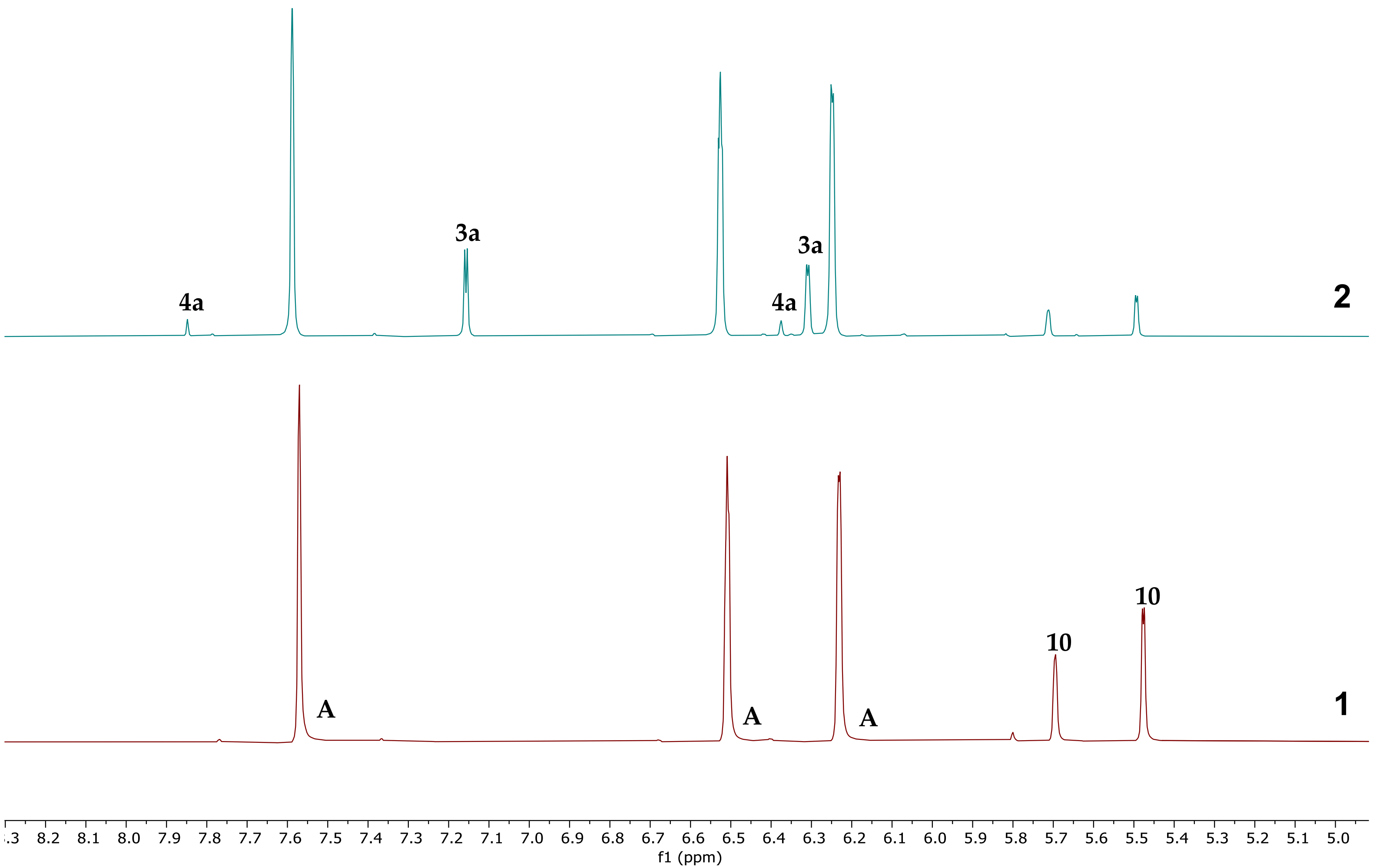

Figure S26. ^{1}H NMR (500 MHz, THF) **1**: Solution containing dmpe$_2$FeH(2-Me-furyl) **10**, dmpe$_2$FeH$_2$ **7**, dmpe$_2$FeCl$_2$ **1**, dmpe$_2$FeHCl **6**, 2-methylfuran **A** (xs.), and HBpin (xs.)**,** 20 h at 60 °C. **2**: After blue light irradiation for 3 h, development of borylated 2-methylfuran **3a/4a**.

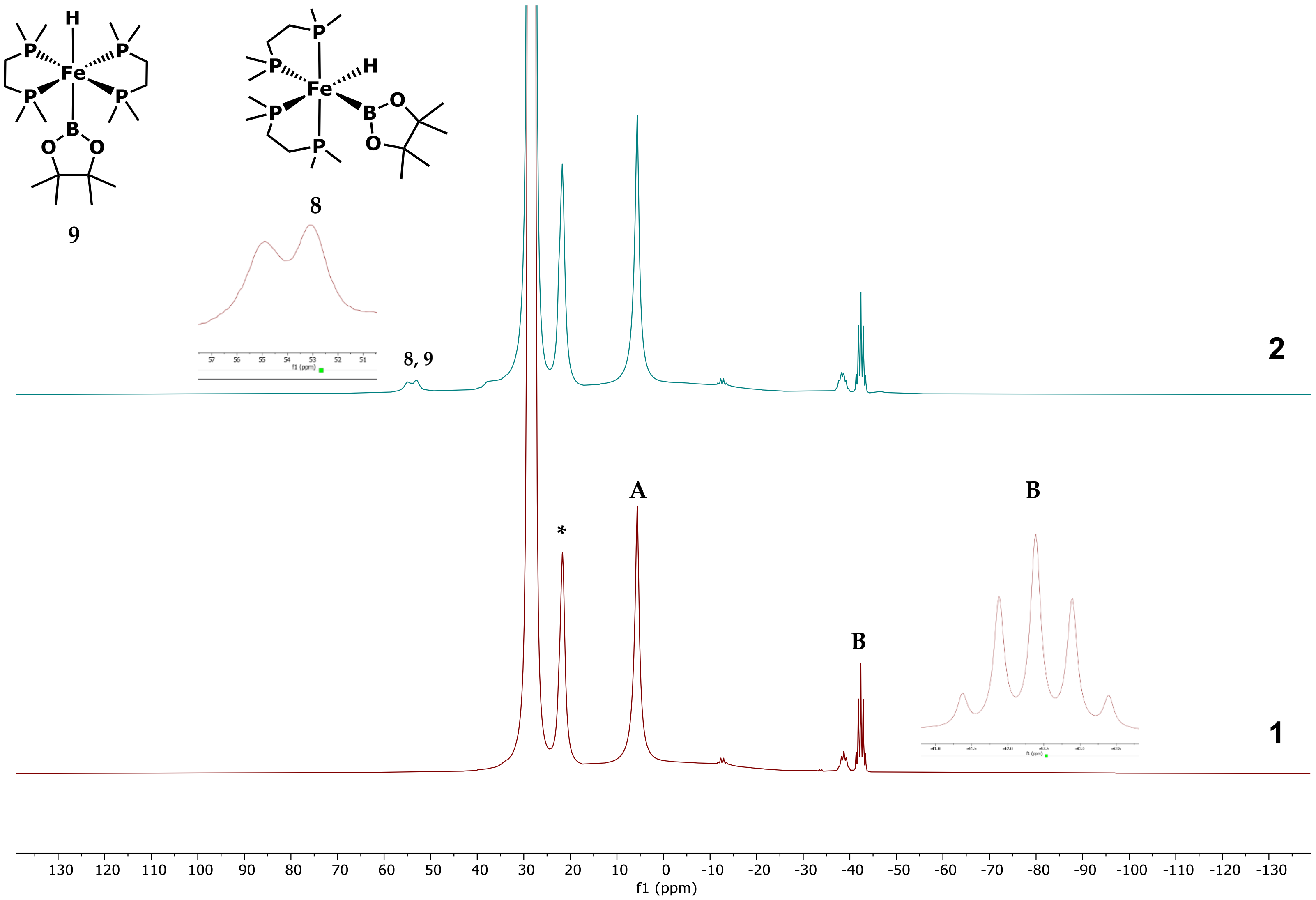

Figure S27. ^{11}B NMR (160 MHz, $CDCl_3$) **1**: Solution containing dmpe$_2$FeH(2-Me-furyl) **10**, dmpe$_2$FeH$_2$ **7**, dmpe$_2$FeCl$_2$ **1**, dmpe$_2$FeHCl **6**, and HBpin (xs.)**,** 20 h at 60 °C. **2**: After blue light irradiation for 3 h, development of dmpe$_2$FeH(Bpin) **8** and **9**, **A**: Boronate species, **B**: $[BH_4]^-$, *****: $B(OR)_3$ impurity.

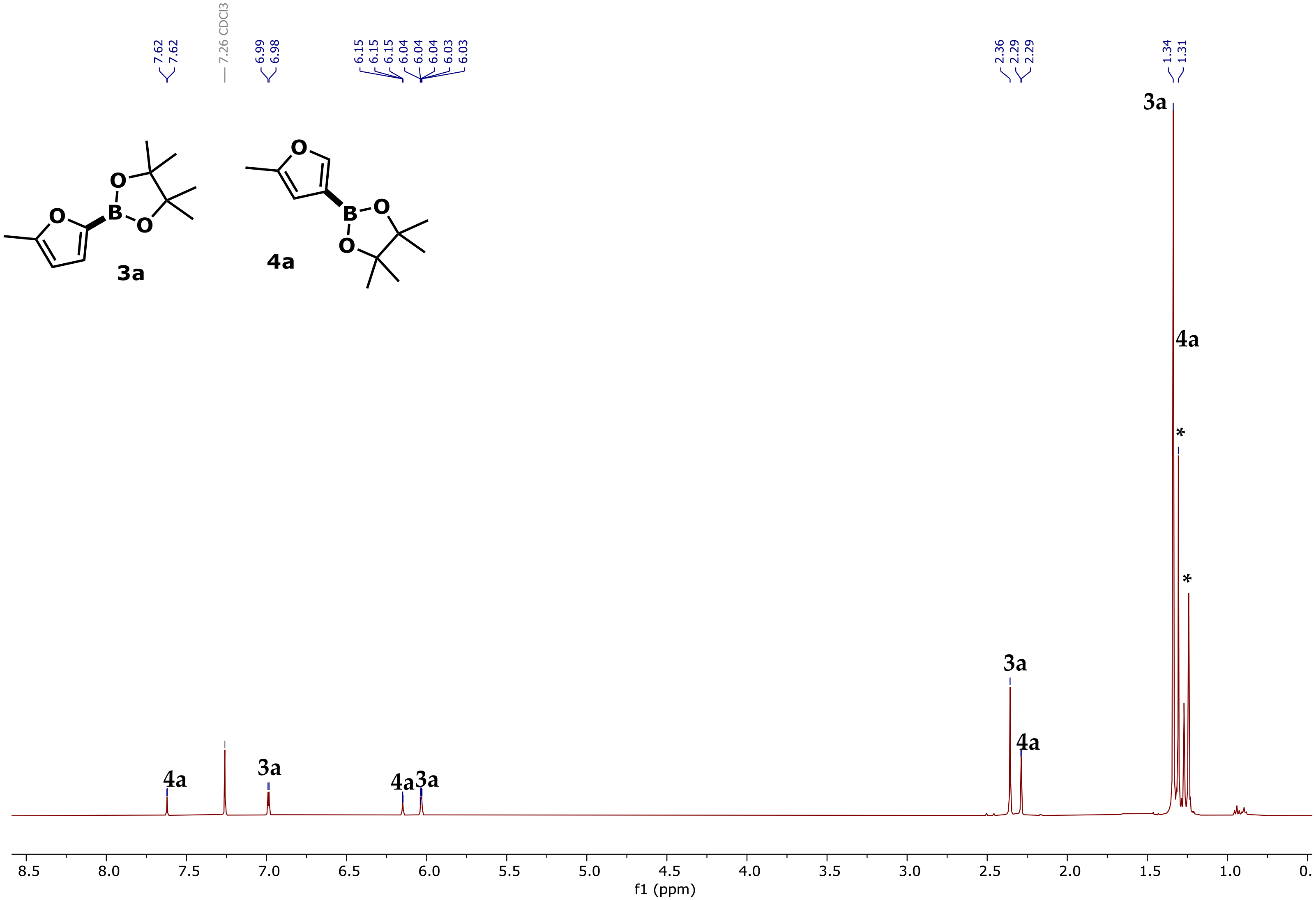

Figure S28. ^{1}H NMR (500 MHz, $CDCl_3$) 4,4,5,5-tetramethyl-2-(5-methylfuran-2-yl)-1,3,2-dioxaborolane **3a,** 4,4,5,5-tetramethyl-2-(5-methylfuran-3-yl)-1,3,2-dioxaborolane **4a,** and *: $B(OR)_3$ impurity.

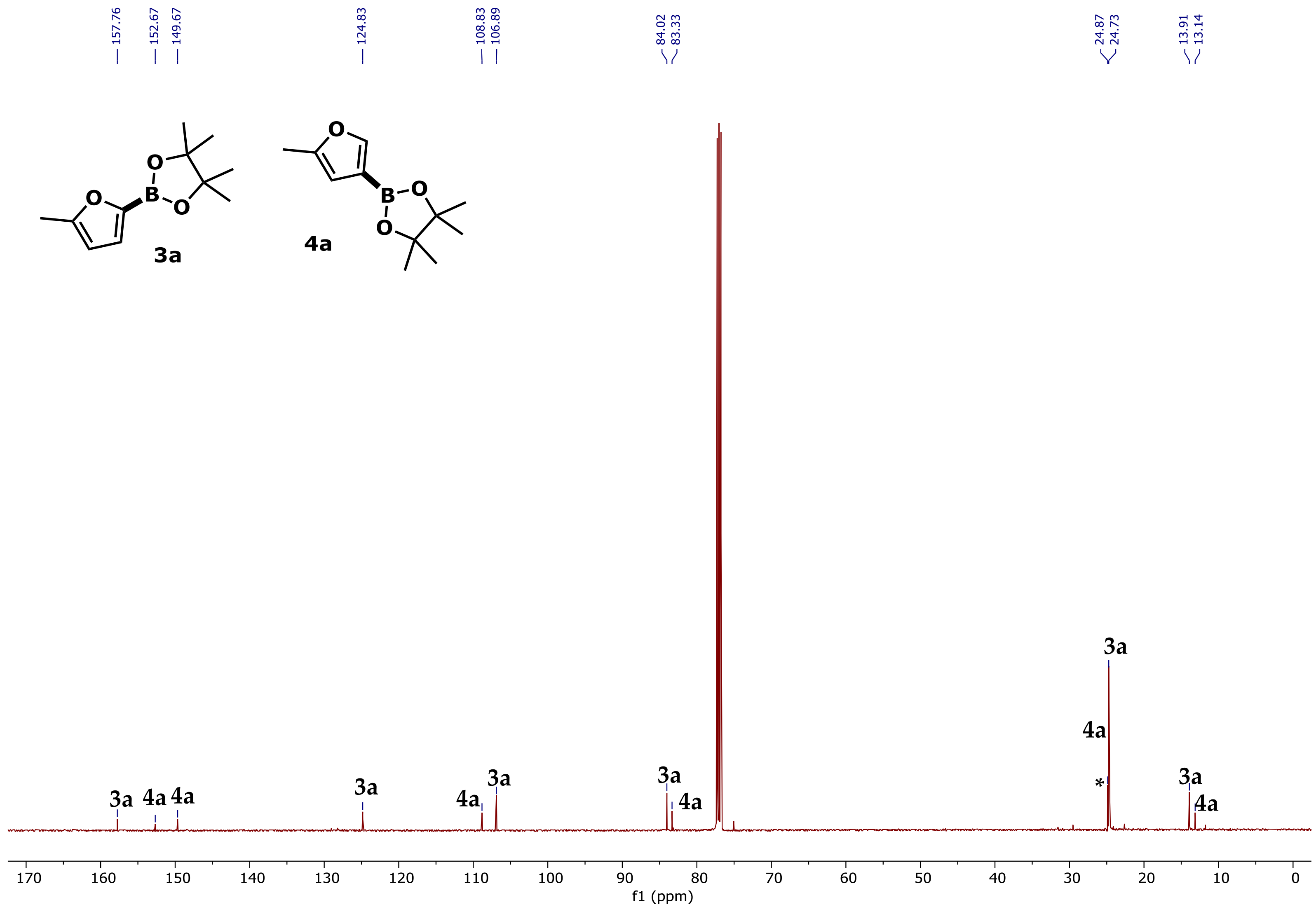

Figure S29. ^{13}C NMR (126 MHz, $CDCl_3$) 4,4,5,5-tetramethyl-2-(5-methylfuran-2-yl)-1,3,2-dioxaborolane **3a,** 4,4,5,5-tetramethyl-2-(5-methylfuran-3-yl)-1,3,2-dioxaborolane **4a,** and *****: $B(OR)_3$ impurity.

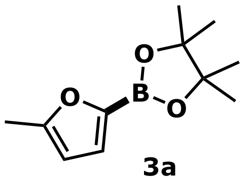

3a

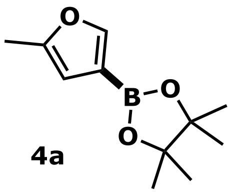

4a

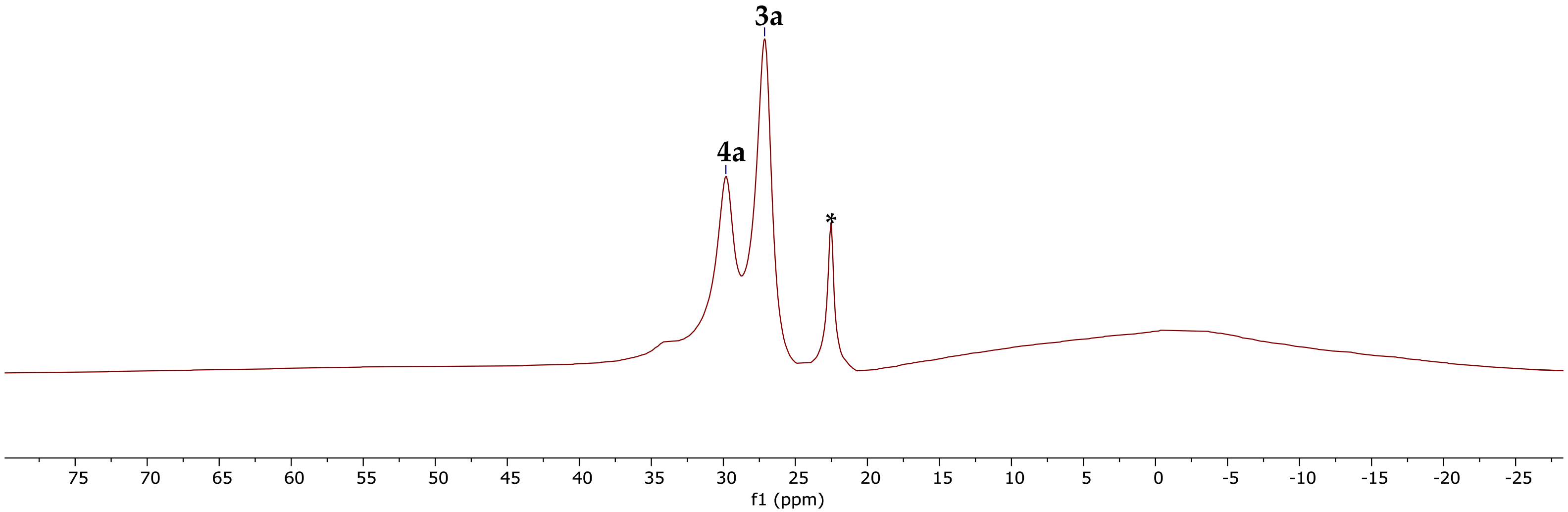

Figure S30. ^{11}B NMR (160 MHz, $CDCl_3$) 4,4,5,5-tetramethyl-2-(5-methylfuran-2-yl)-1,3,2-dioxaborolane **3a**, 4,4,5,5-tetramethyl-2-(5-methylfuran-3-yl)-1,3,2-dioxaborolane **4a**, and *: $B(OR)_3$ impurity.

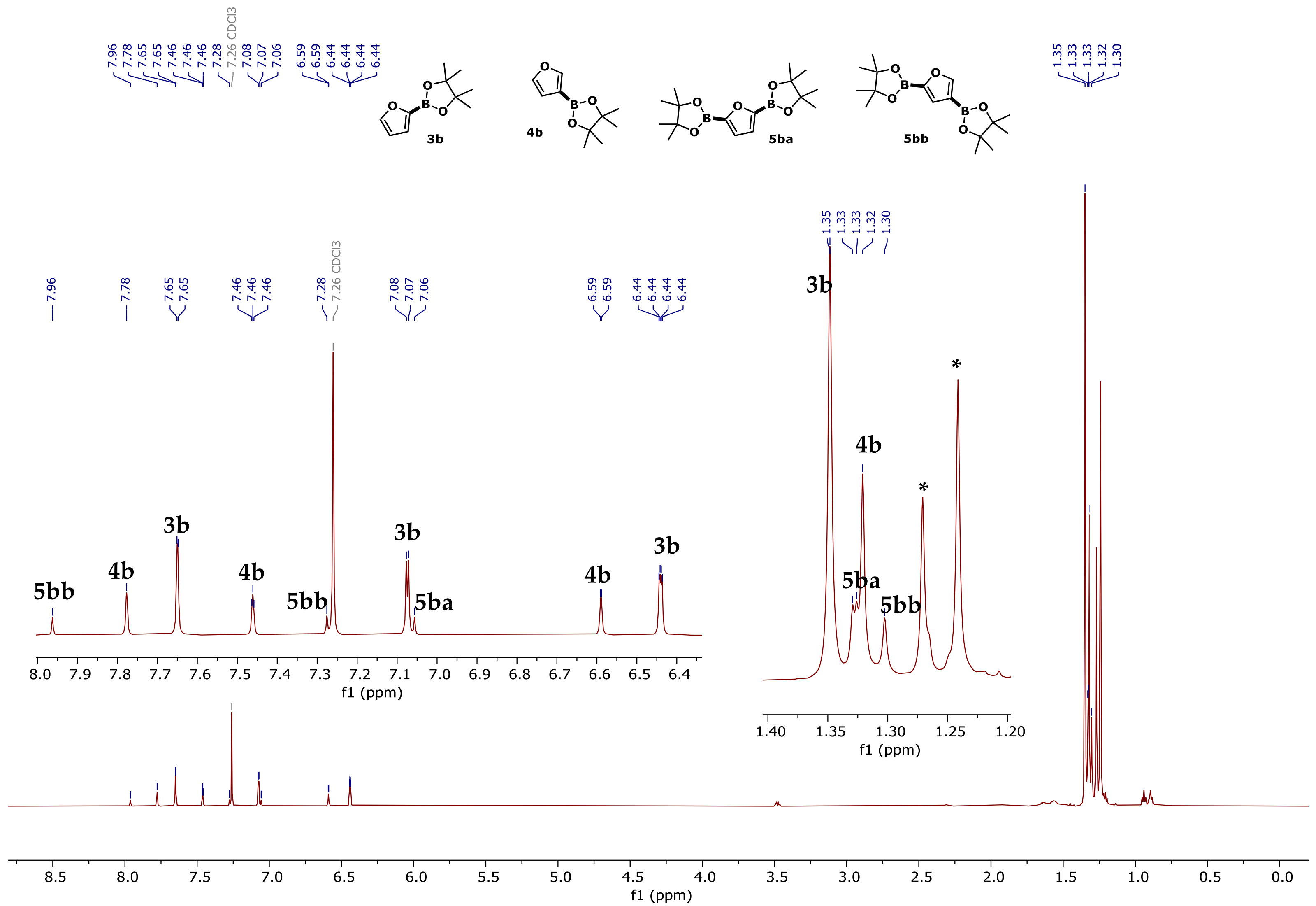

Figure S31. ^{1}H NMR (600 MHz, $CDCl_3$) 4,4,5,5-tetramethyl-2-(furanyl-2-yl)-1,3,2-dioxaborolane **3b**, 4,4,5,5-tetramethyl-2-(furanyl-3-yl)-1,3,2-dioxaborolane **4b**, 2,5-bis(4,4,5,5-tetramethyl-1,3,2-dioxaborolan-2-yl)furan **5ba**, 2,4-bis(4,4,5,5-tetramethyl-1,3,2-dioxaborolan-2-yl)furan **5bb**, and *: B(OR)$_3$ impurity.

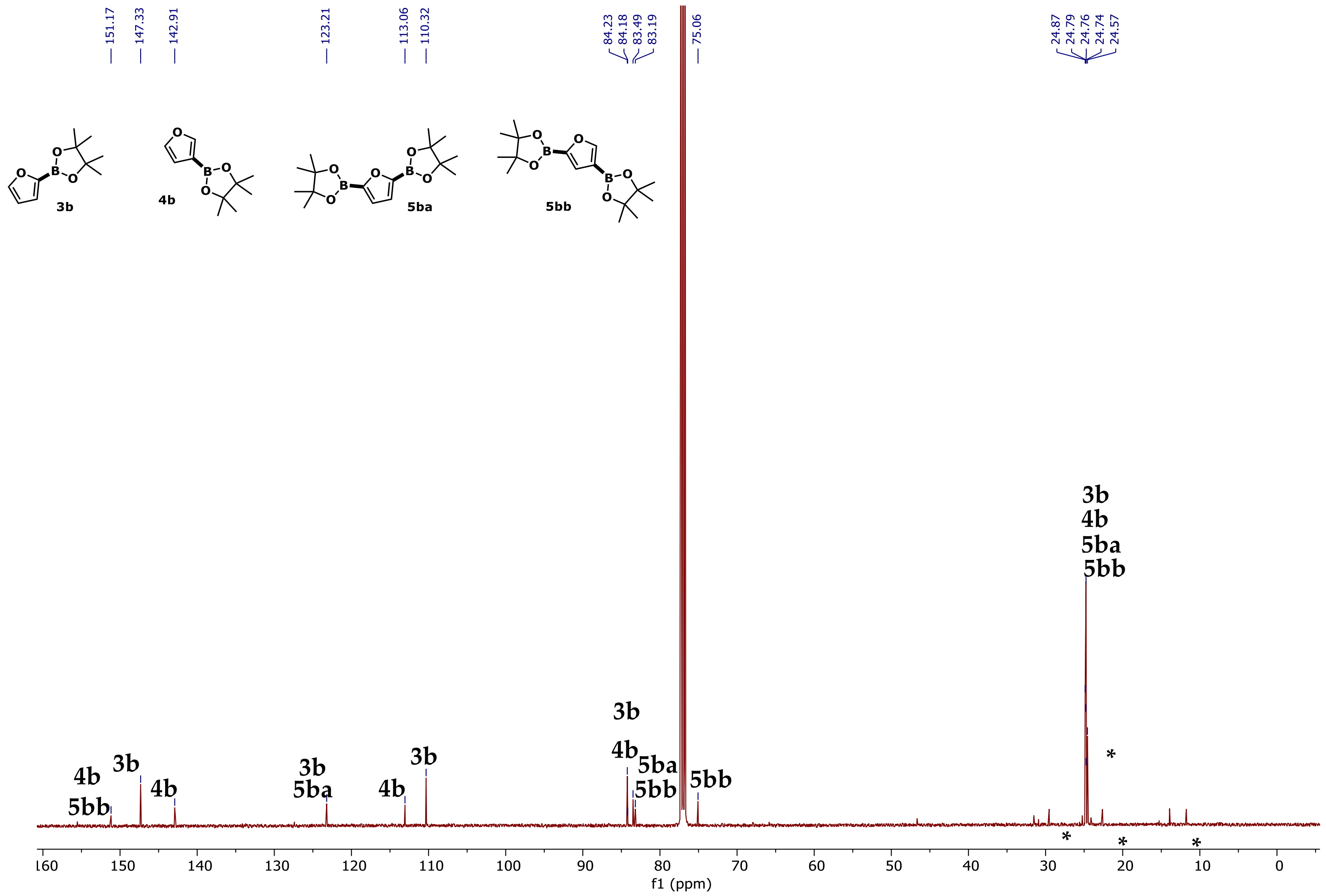

Figure S32. ^{13}C NMR (126 MHz, $CDCl_3$) 4,4,5,5-tetramethyl-2-(furanyl-2-yl)-1,3,2-dioxaborolane **3b**, 4,4,5,5-tetramethyl-2-(furanyl-3-yl)-1,3,2-dioxaborolane **4b**, 2,5-bis(4,4,5,5-tetramethyl-1,3,2-dioxaborolan-2-yl)furan **5ba**, 2,4-bis(4,4,5,5-tetramethyl-1,3,2-dioxaborolan-2-yl)furan **5bb**, and *: $B(OR)_3$ impurity.

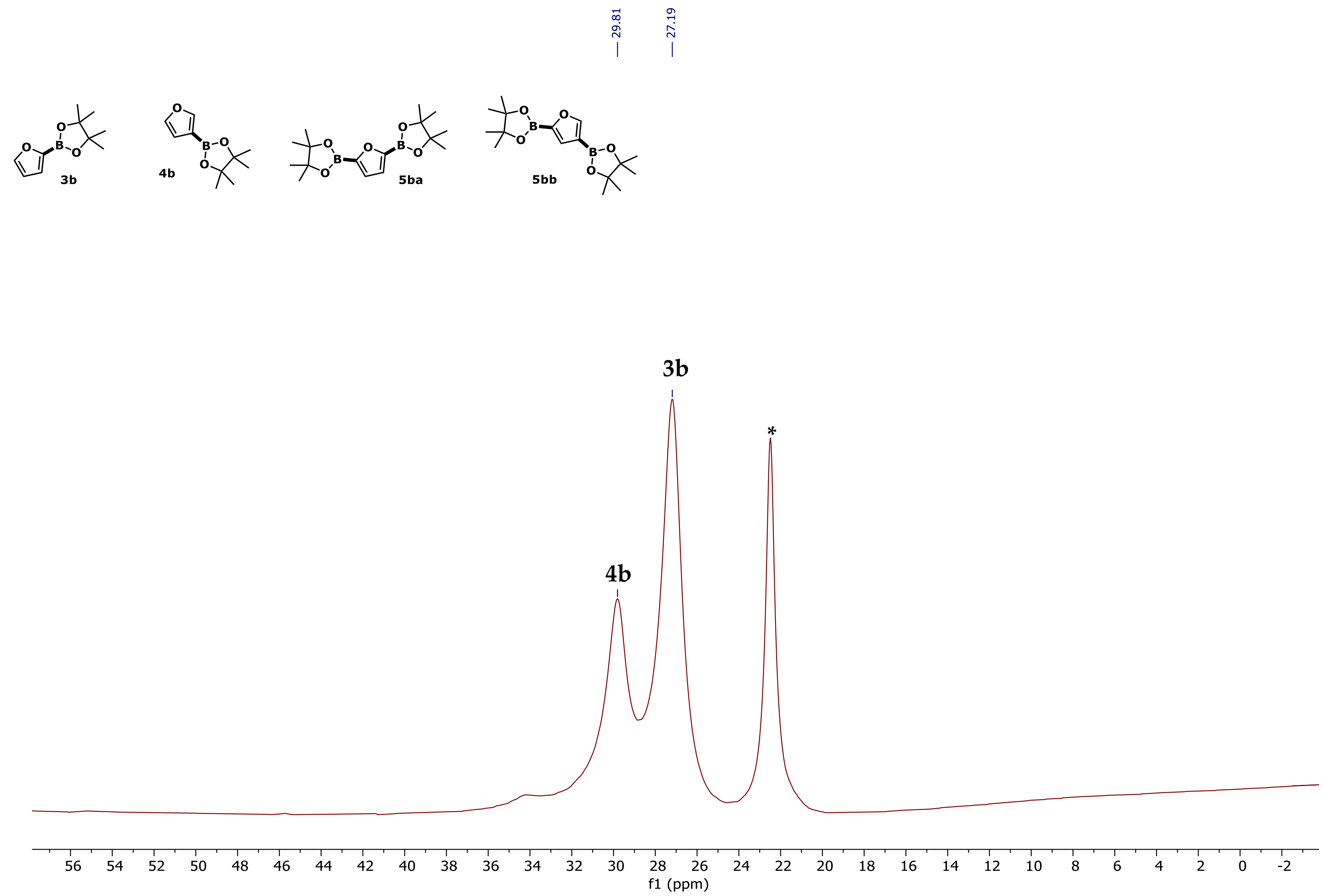

Figure S33. ^{11}B NMR (160 MHz, $CDCl_3$) 4,4,5,5-tetramethyl-2-(furanyl-2-yl)-1,3,2-dioxaborolane **3b**, 4,4,5,5-tetramethyl-2-(furanyl-3-yl)-1,3,2-dioxaborolane **4b**, 2,5-bis(4,4,5,5-tetramethyl-1,3,2-dioxaborolan-2-yl)furan **5ba**, 2,4-bis(4,4,5,5-tetramethyl-1,3,2-dioxaborolan-2-yl)furan **5bb**, and *: $B(OR)_3$ impurity.

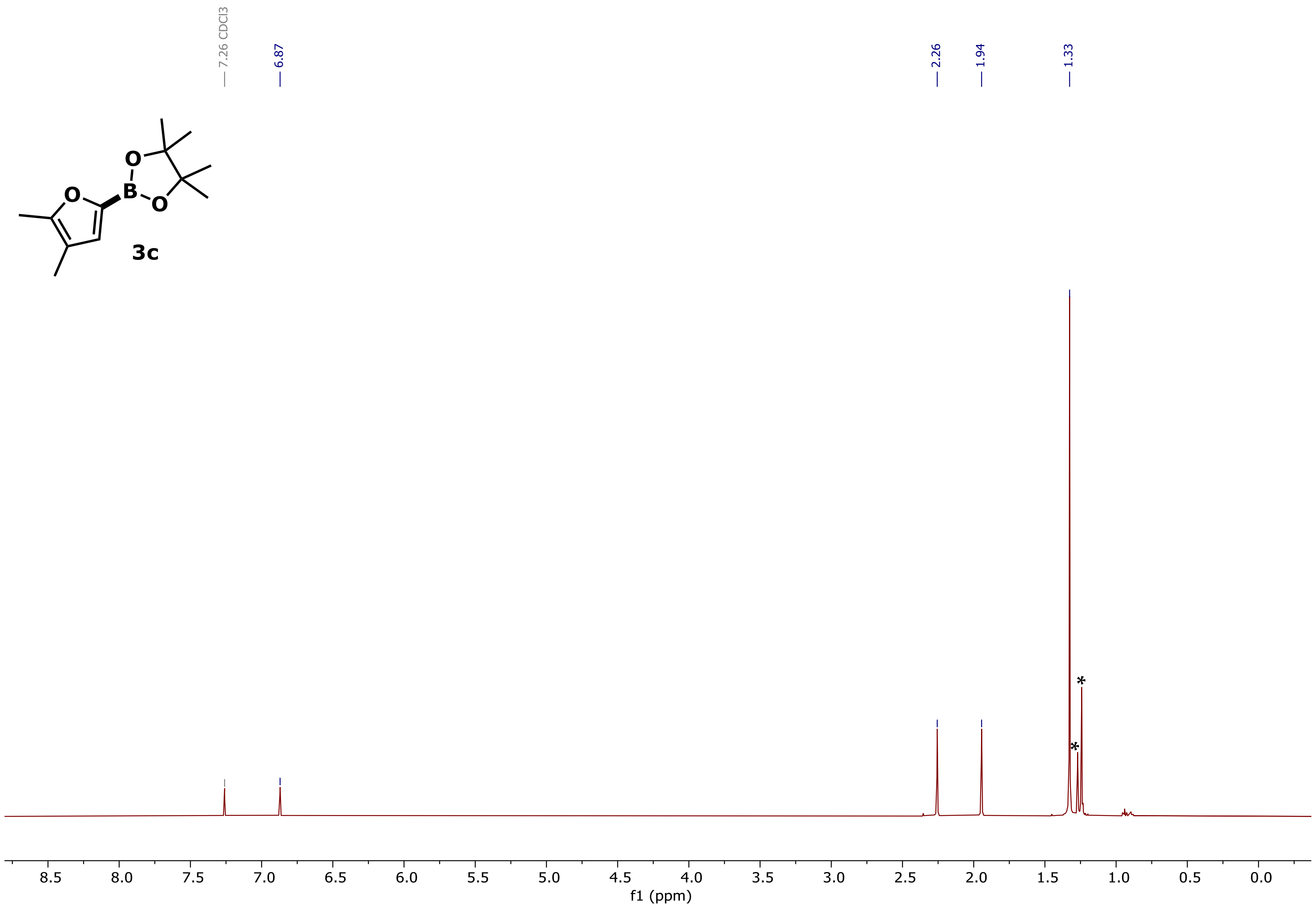

Figure S34. ^{1}H NMR (500 MHz, $CDCl_3$) 4,4,5,5-tetramethyl-2-(4,5-dimethylfuran-2-yl)-1,3,2-dioxaborolane **3c** and *: $B(OR)_3$ impurity.

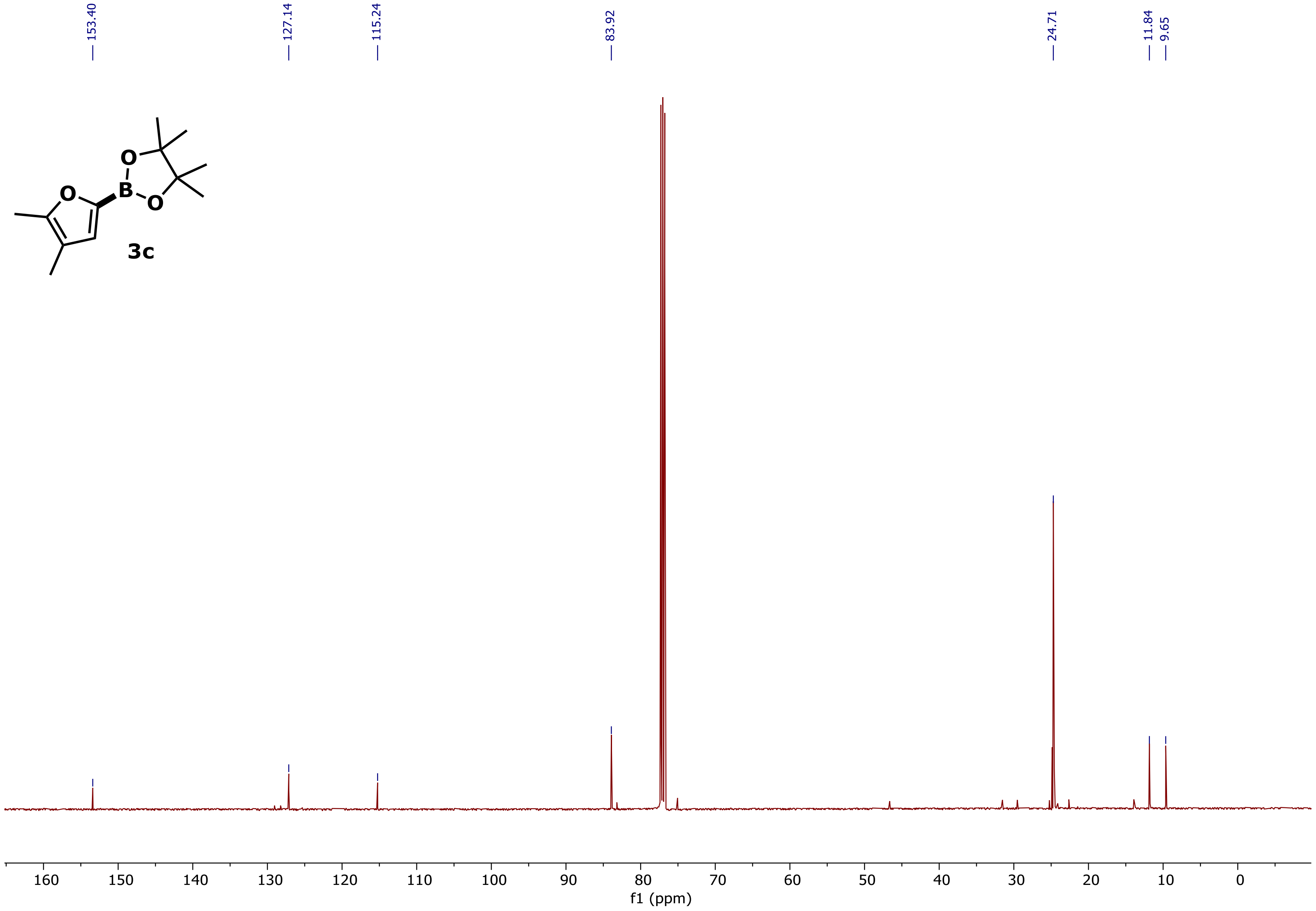

Figure S35. ^{13}C NMR (126 MHz, $CDCl_3$) 4,4,5,5-tetramethyl-2-(4,5-dimethylfuran-2-yl)-1,3,2-dioxaborolane **3c**

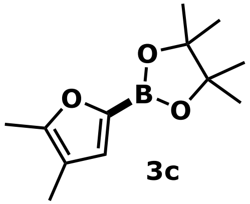

3c

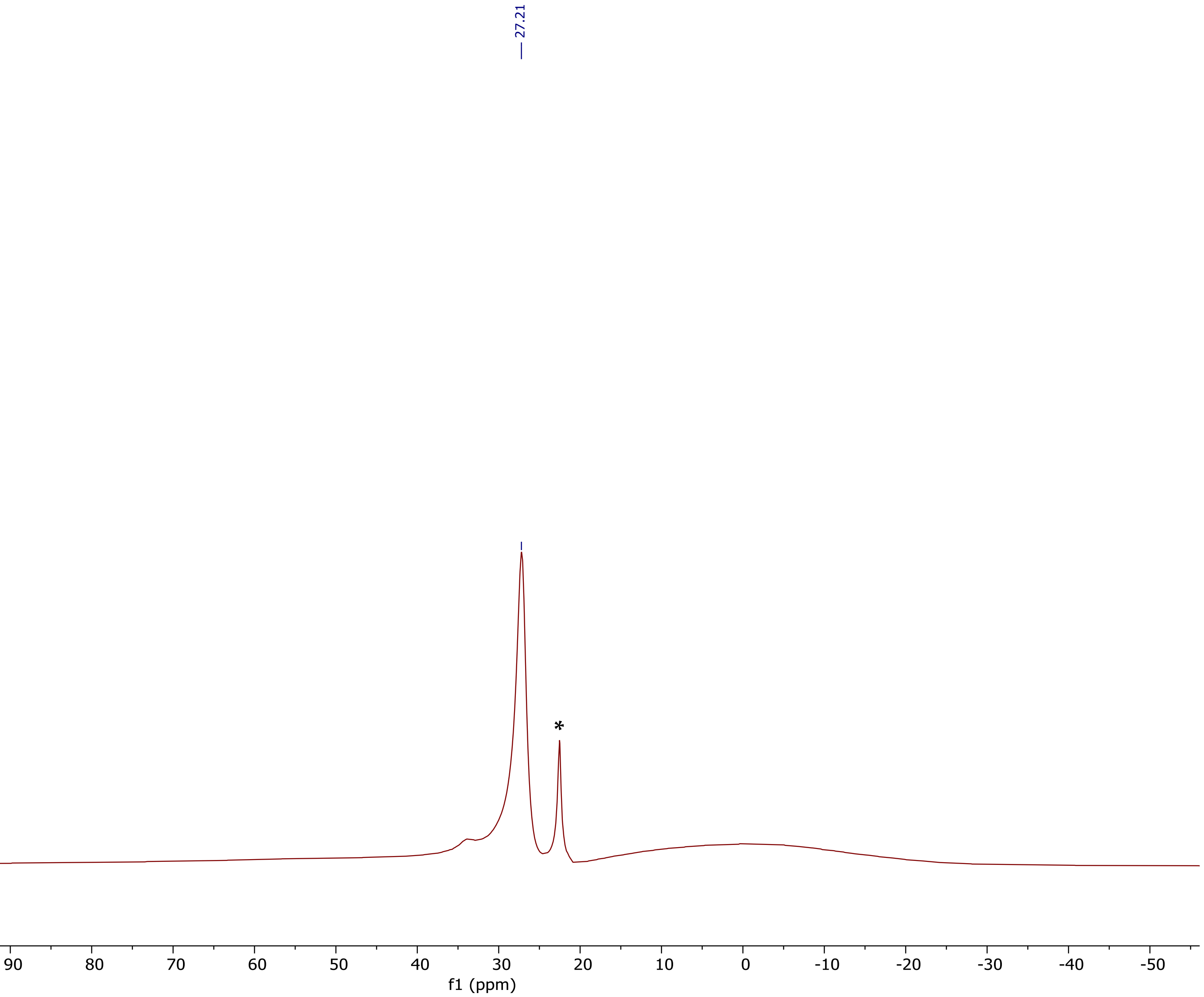

Figure S36. ^{11}B NMR (160 MHz, $CDCl_3$) 4,4,5,5-tetramethyl-2-(4,5-dimethylfuran-2-yl)-1,3,2-dioxaborolane **3c** and *: $B(OR)_3$ impurity.

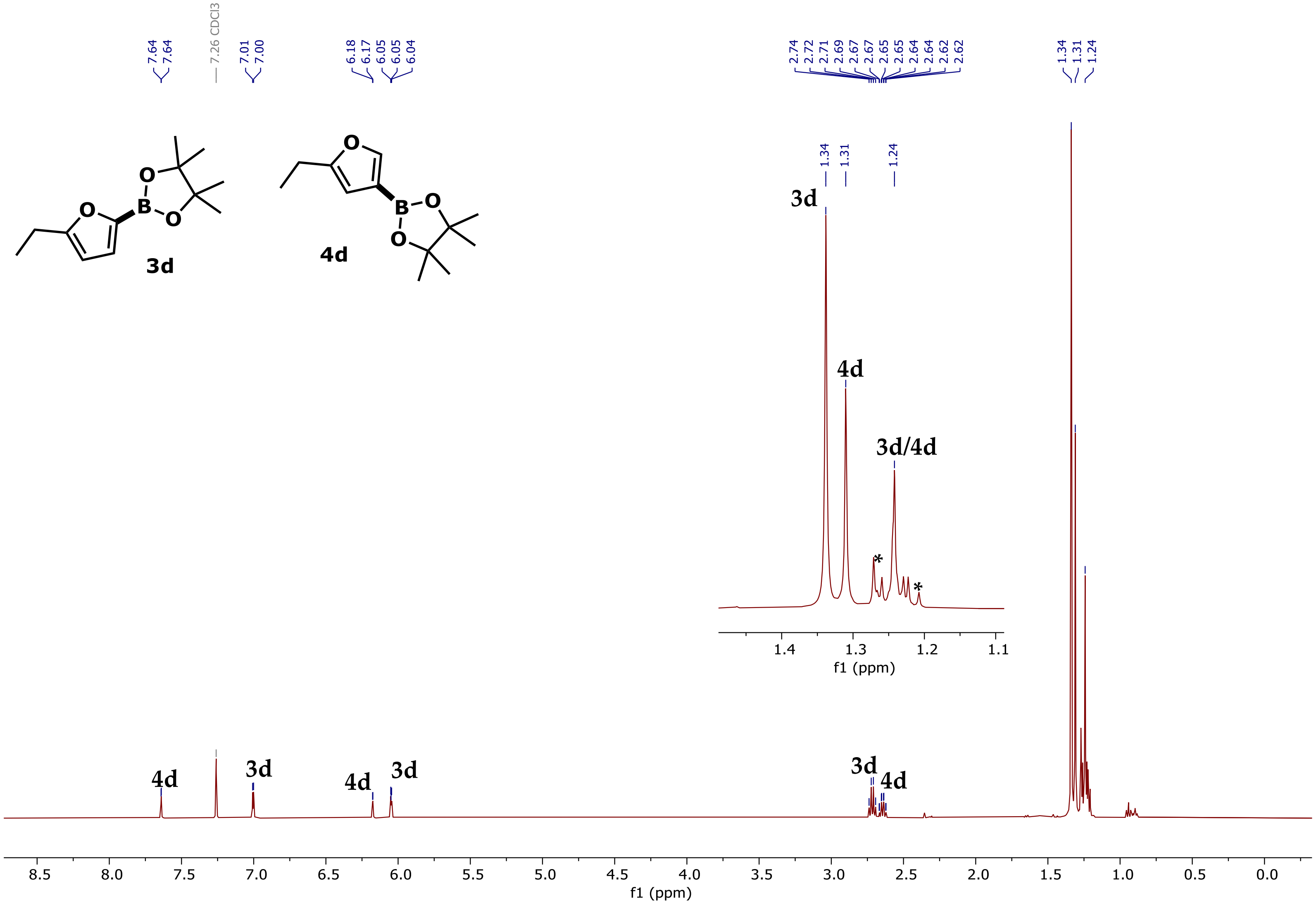

Figure S37. ^{1}H NMR (500 MHz, $CDCl_3$) 4,4,5,5-tetramethyl-2-(5-ethylfuran-2-yl)-1,3,2-dioxaborolane **3d**, 4,4,5,5-tetramethyl-2-(5-methylfuran-3-yl)-1,3,2-dioxaborolane **4d**, and *: $B(OR)_3$ impurity.

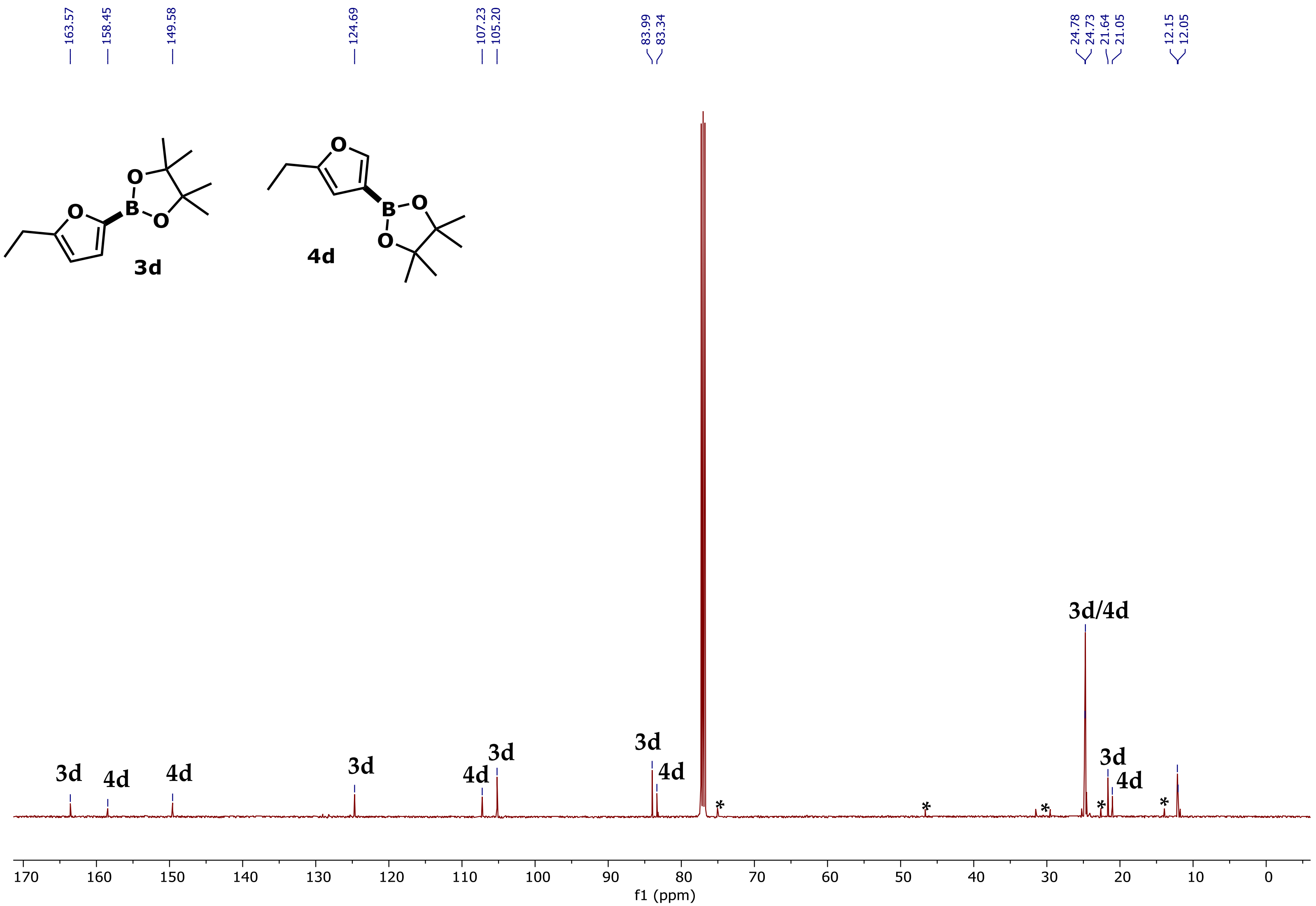

Figure S38. ^{13}C NMR (126 MHz, $CDCl_3$) 4,4,5,5-tetramethyl-2-(5-ethylfuran-2-yl)-1,3,2-dioxaborolane **3d**, 4,4,5,5-tetramethyl-2-(5-methylfuran-3-yl)-1,3,2-dioxaborolane **4d**, and *: $B(OR)_3$ impurity.

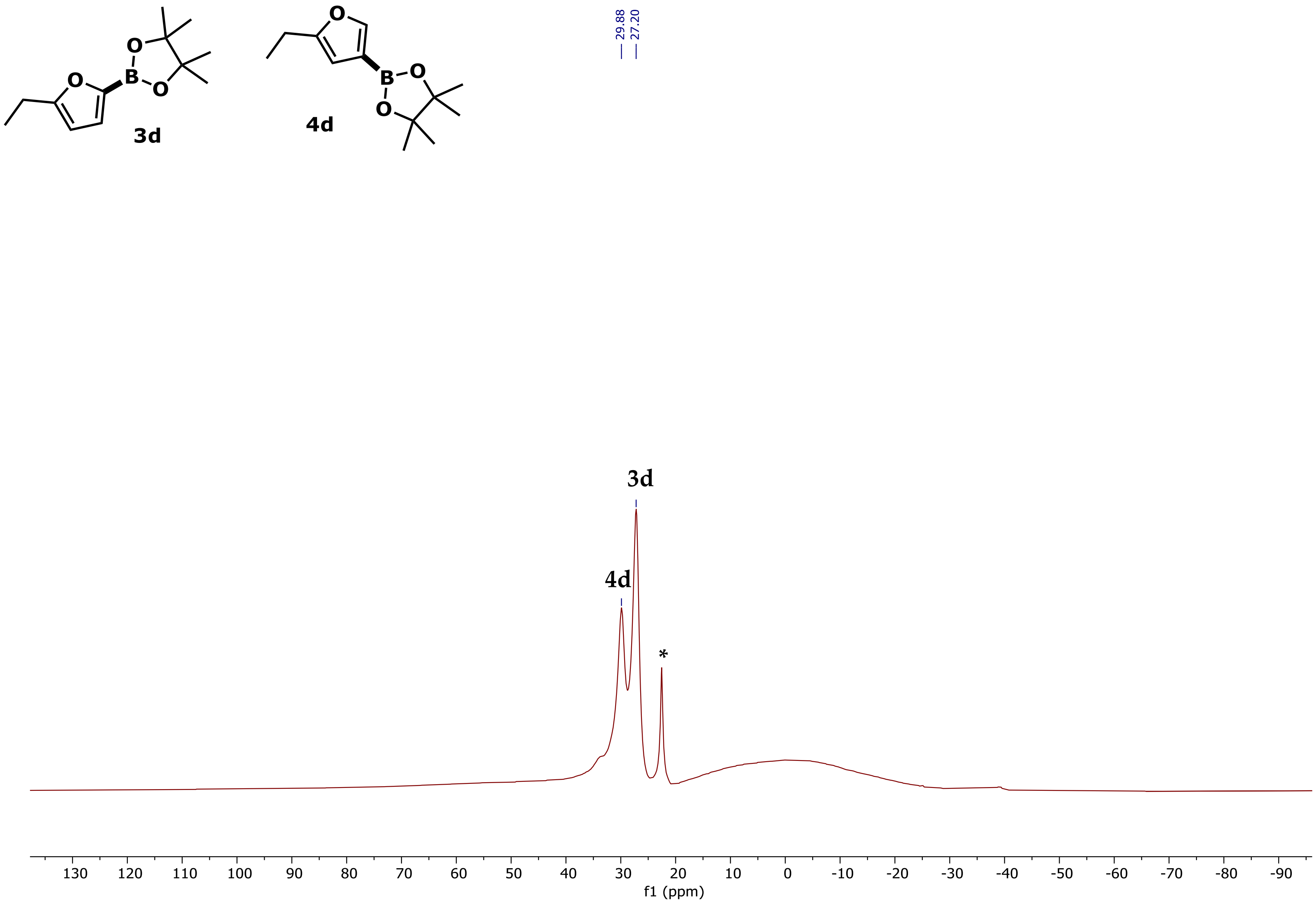

Figure S39. ^{11}B NMR (160 MHz, $CDCl_3$) 4,4,5,5-tetramethyl-2-(5-ethylfuran-2-yl)-1,3,2-dioxaborolane **3d**, 4,4,5,5-tetramethyl-2-(5-methylfuran-3-yl)-1,3,2-dioxaborolane **4d**, and *: $B(OR)_3$ impurity.

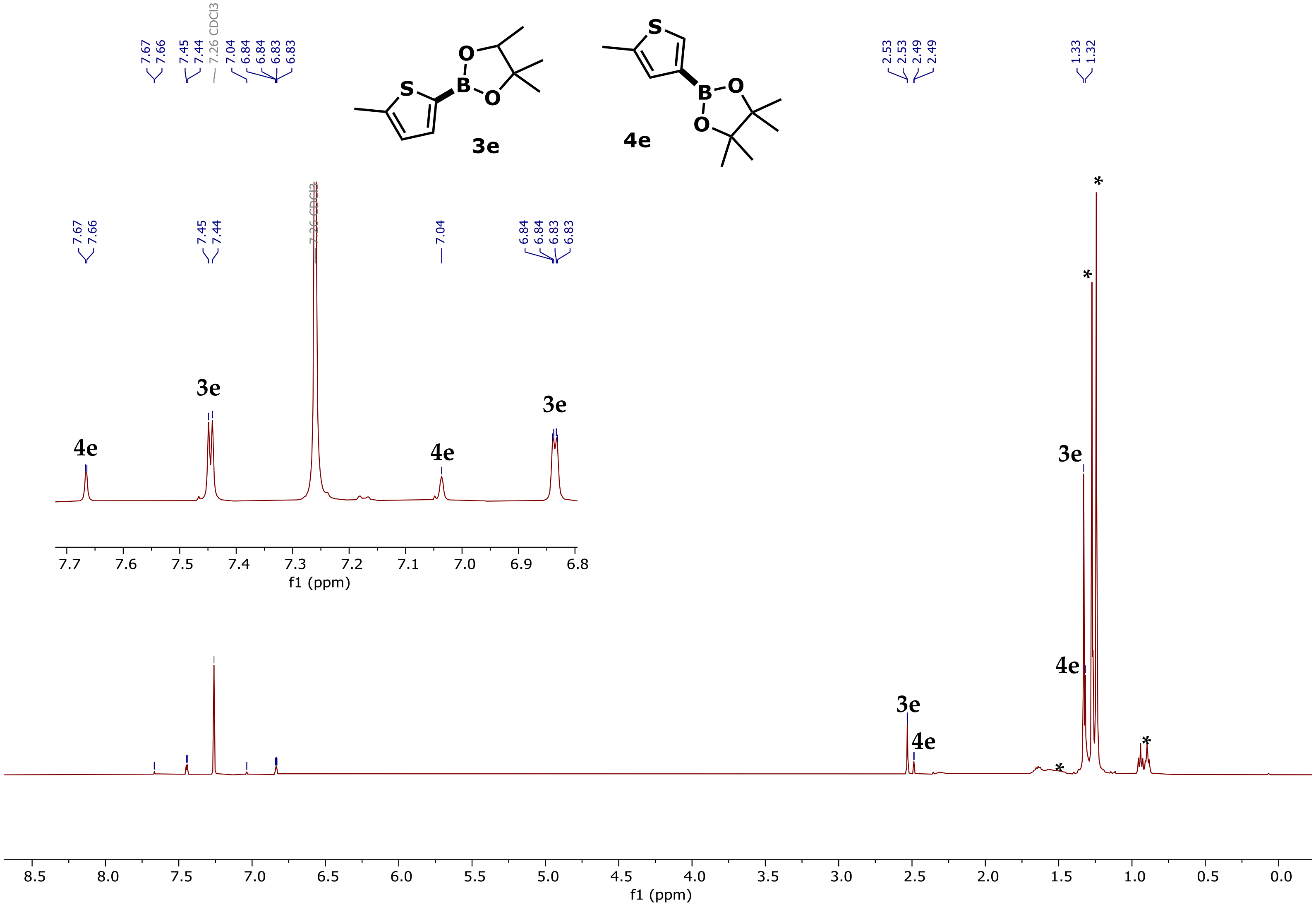

Figure S40. ^{1}H NMR (500 MHz, $CDCl_3$) 4,4,5,5-tetramethyl-2-(5-methylthiophen-2-yl)-1,3,2-dioxaborolane **3e,** 4,4,5,5-tetramethyl-2-(5-methylthiophen-3-yl)-1,3,2-**4e**, and *: impurity.

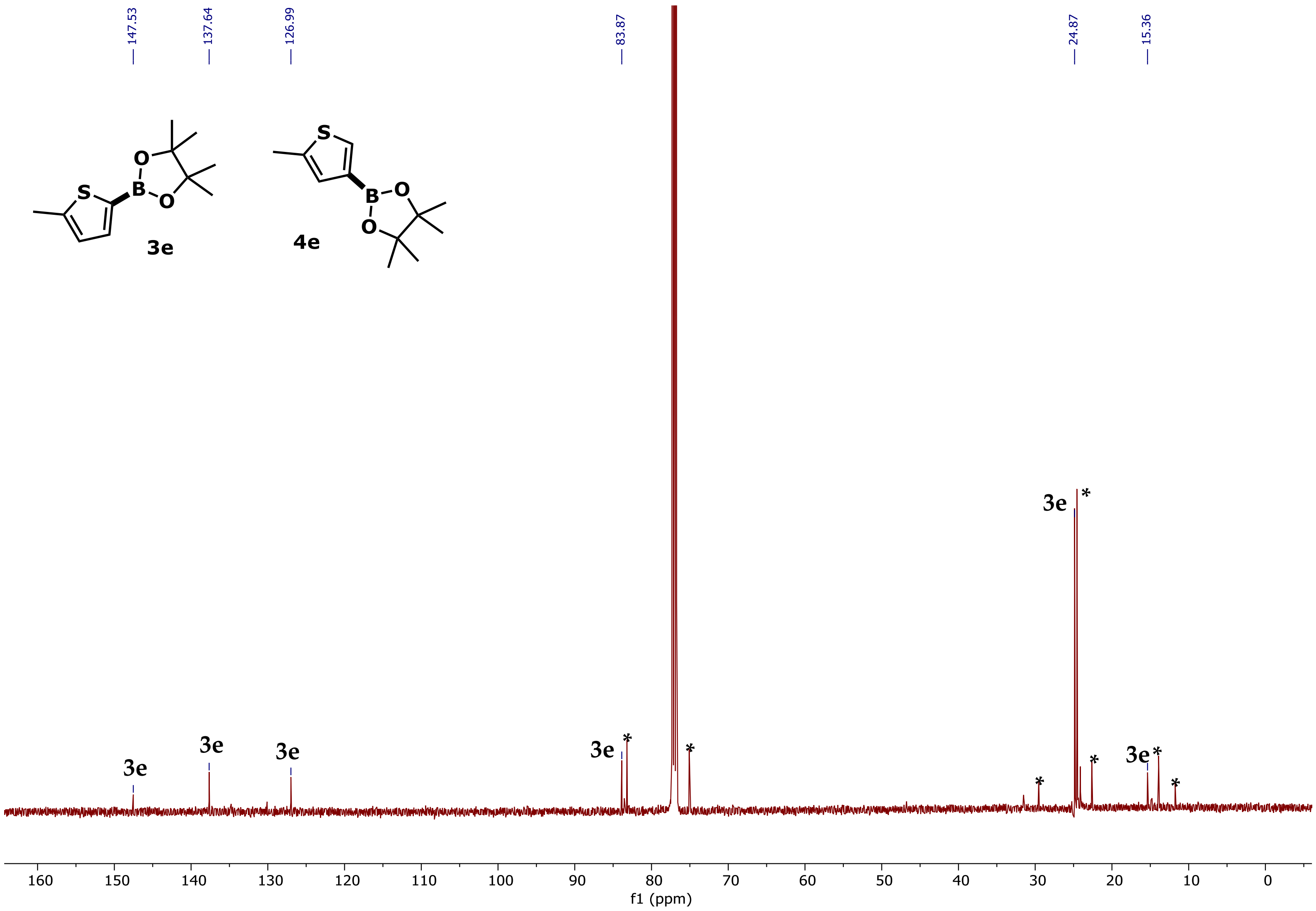

Figure S41. ^{13}C NMR (126 MHz, $CDCl_3$) 4,4,5,5-tetramethyl-2-(5-methylthiophen-2-yl)-1,3,2-dioxaborolane **3e,** 4,4,5,5-tetramethyl-2-(5-methylthiophen-3-yl)-1,3,2-**4e**, and *: impurity.

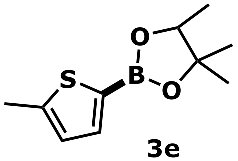

3e

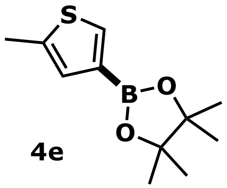

4e

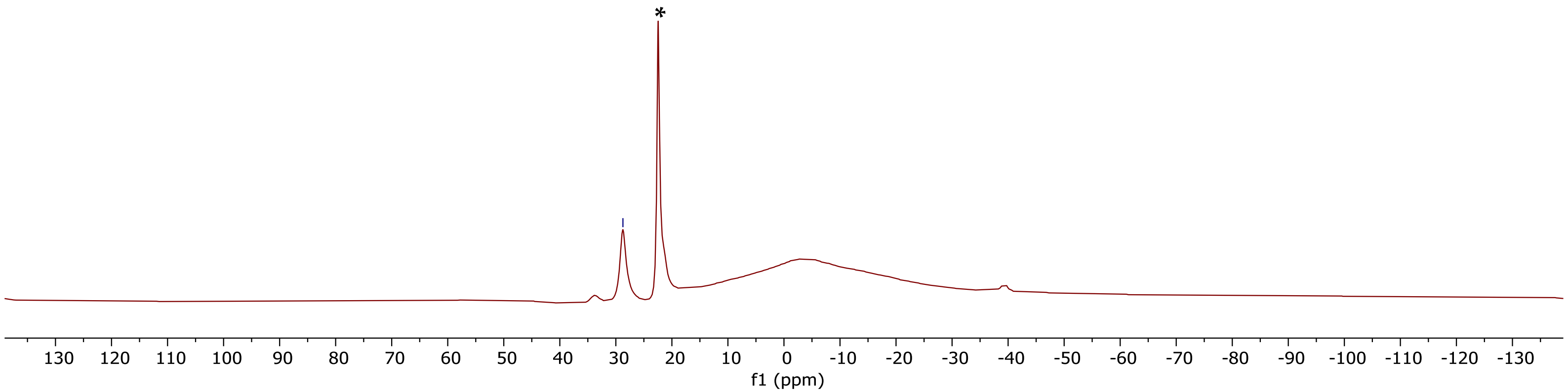

Figure S42. ^{11}B NMR (160 MHz, $CDCl_3$) 4,4,5,5-tetramethyl-2-(5-methylthiophen-2-yl)-1,3,2-dioxaborolane **3e,** 4,4,5,5-tetramethyl-2-(5-methylthiophen-3-yl)-1,3,2- **4e**, and *: impurity.

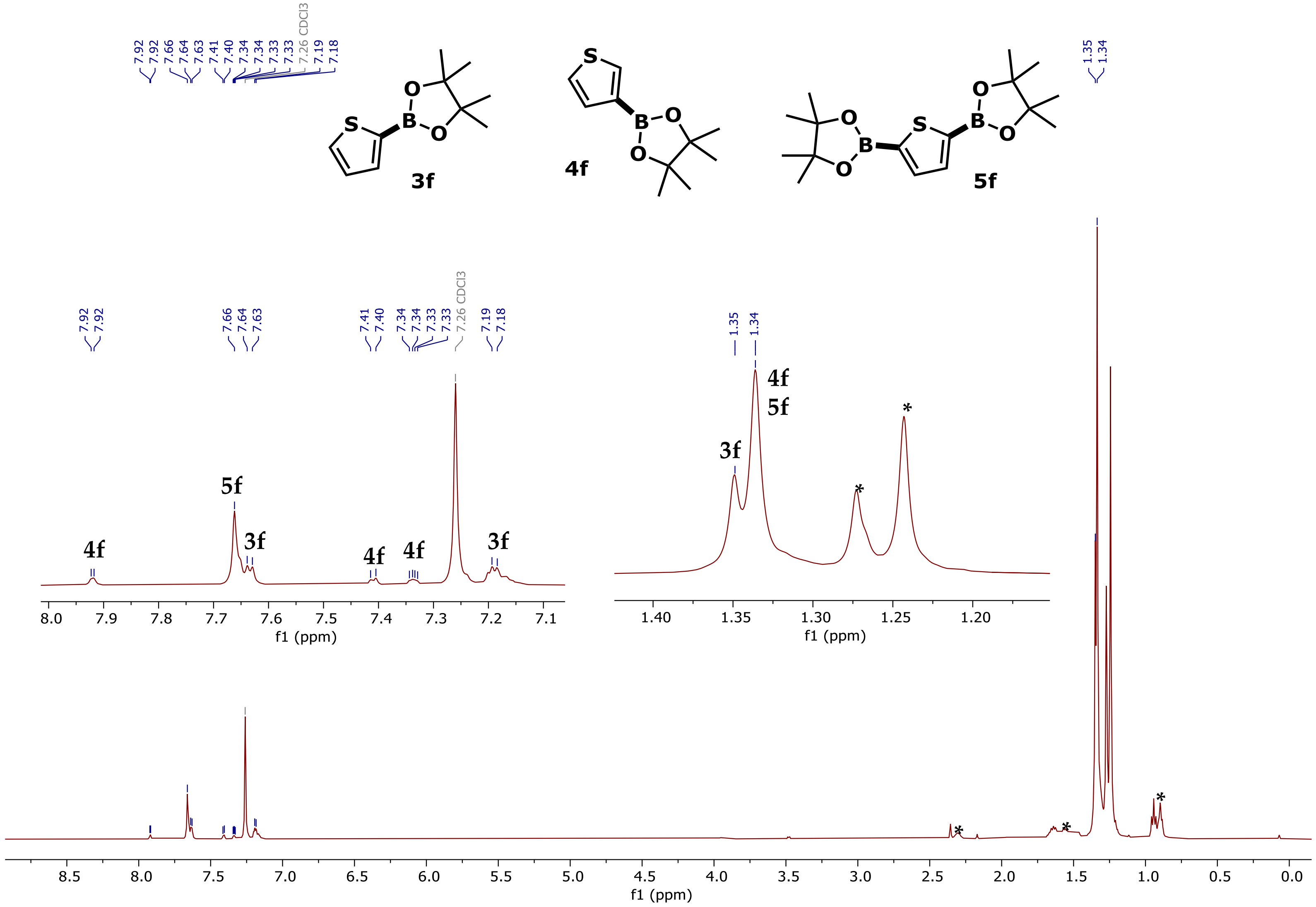

Figure S43. ^{1}H NMR (500 MHz, $CDCl_3$) 4,4,5,5-tetramethyl-2-(thiophene-2-yl)-1,3,2-dioxaborolane **3f**, 4,4,5,5-tetramethyl-2-(thiophene-3-yl)-1,3,2-dioxaborolane **4f**, 2,5-bis(4,4,5,5-tetramethyl-1,3,2-dioxaborolan-2-yl)thiophene **5f,** and *: impurity.

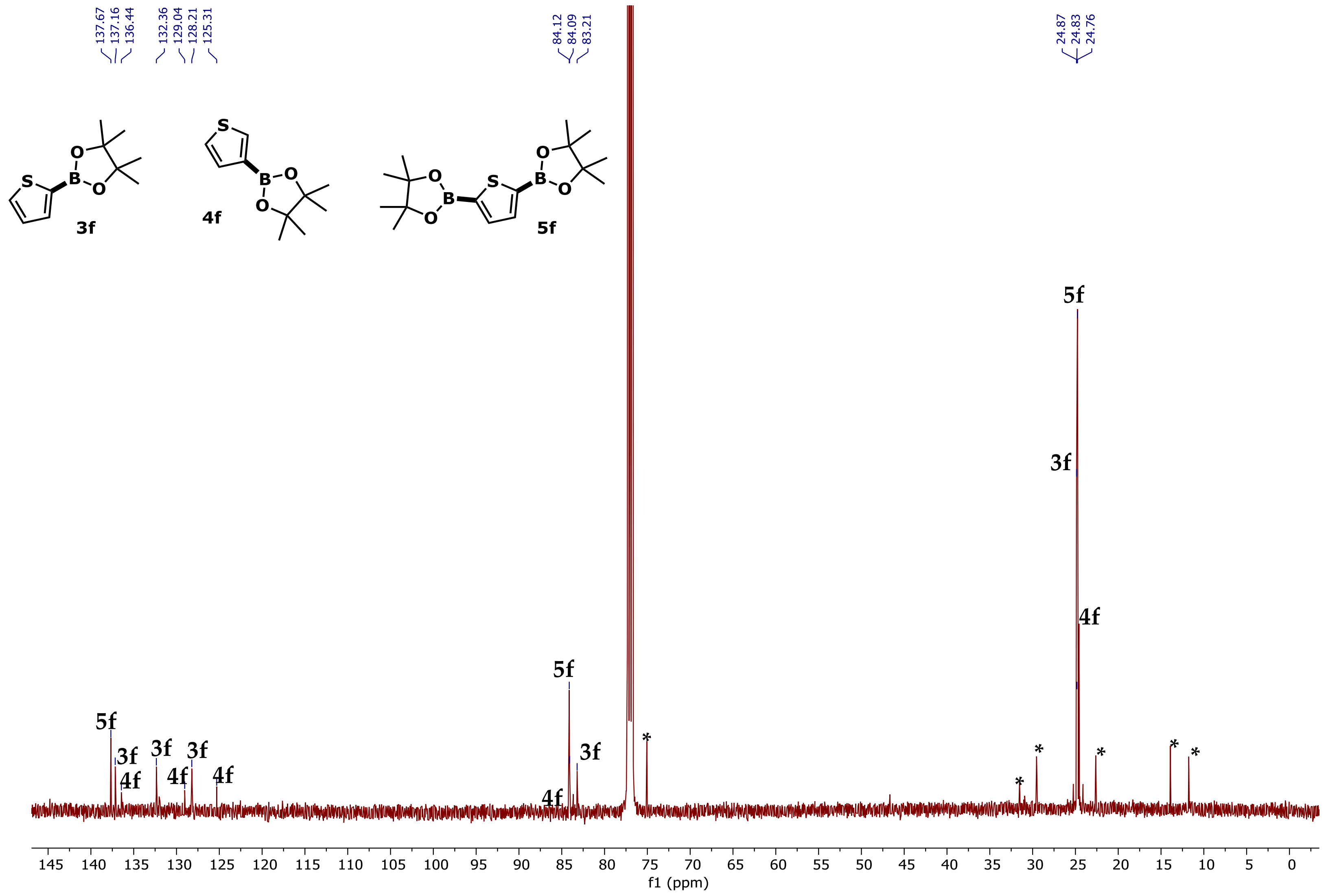

Figure S44. ^{13}C NMR (126 MHz, $CDCl_3$) 4,4,5,5-tetramethyl-2-(thiophene-2-yl)-1,3,2-dioxaborolane **3f**, 4,4,5,5-tetramethyl-2-(thiophene-3-yl)-1,3,2-dioxaborolane **4f**, 2,5-bis(4,4,5,5-tetramethyl-1,3,2-dioxaborolan-2-yl)thiophene **5f,** and *****: impurity.

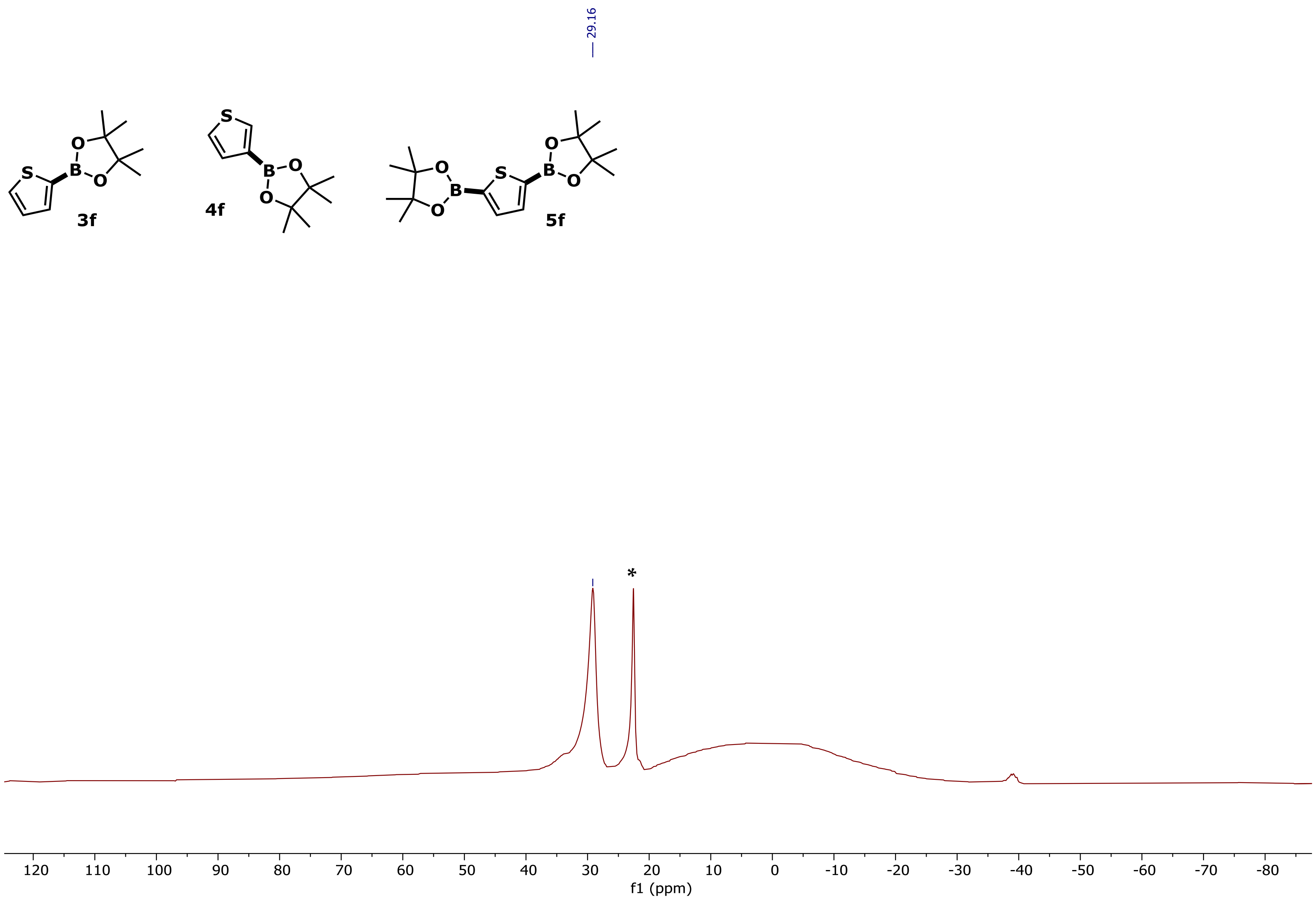

Figure S45. ^{11}B NMR (160 MHz, $CDCl_3$) 4,4,5,5-tetramethyl-2-(thiophene-2-yl)-1,3,2-dioxaborolane **3f**, 4,4,5,5-tetramethyl-2-(thiophene-3-yl)-1,3,2-dioxaborolane **4f**, 2,5-bis(4,4,5,5-tetramethyl-1,3,2-dioxaborolan-2-yl)thiophene **5f, and** *: impurity.

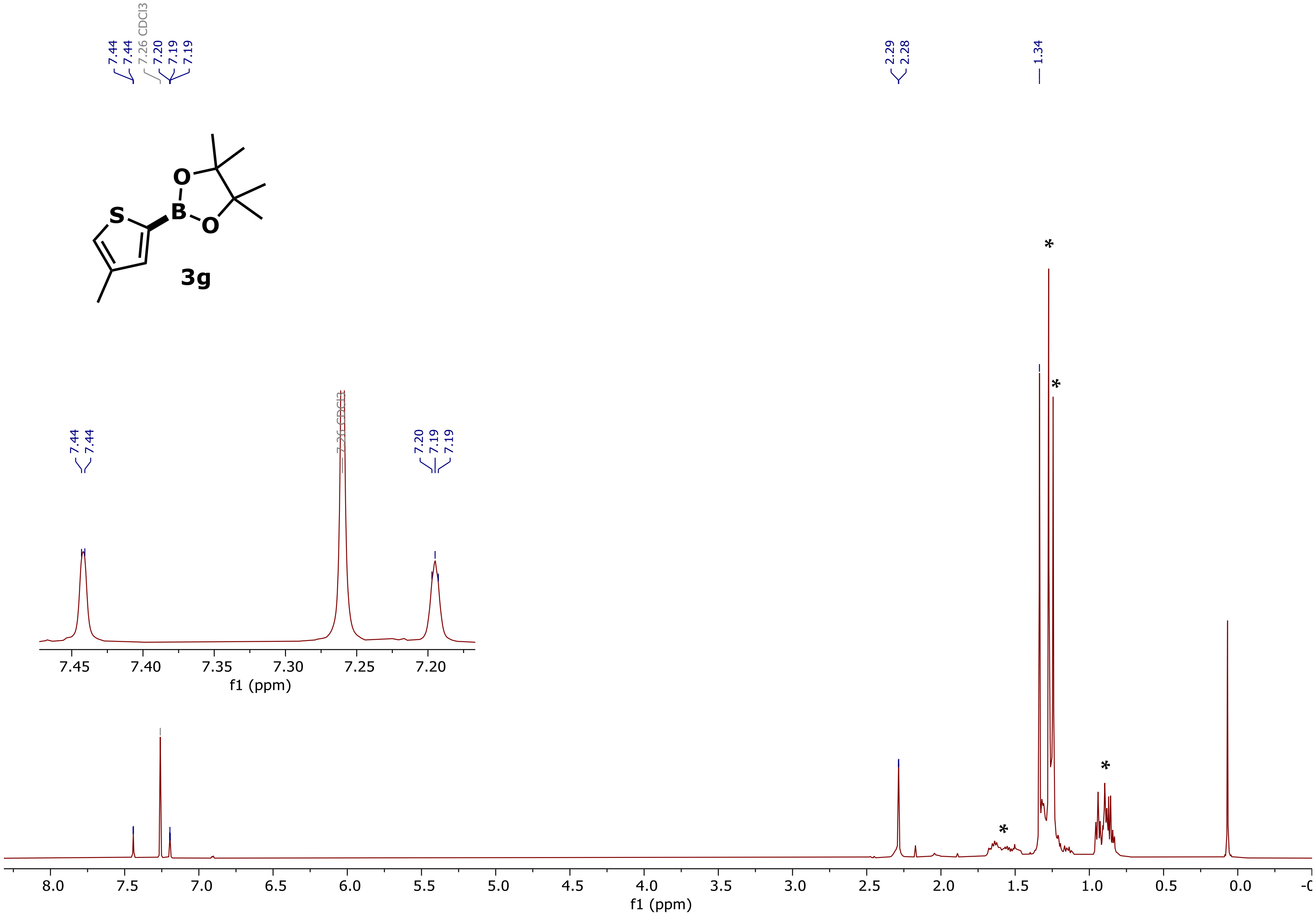

Figure S46. ^{1}H NMR (500 MHz, $CDCl_3$) 4,4,5,5-tetramethyl-2-(4-methylthiophen-2-yl)-1,3,2-dioxaborolane **3g** and *: impurity.

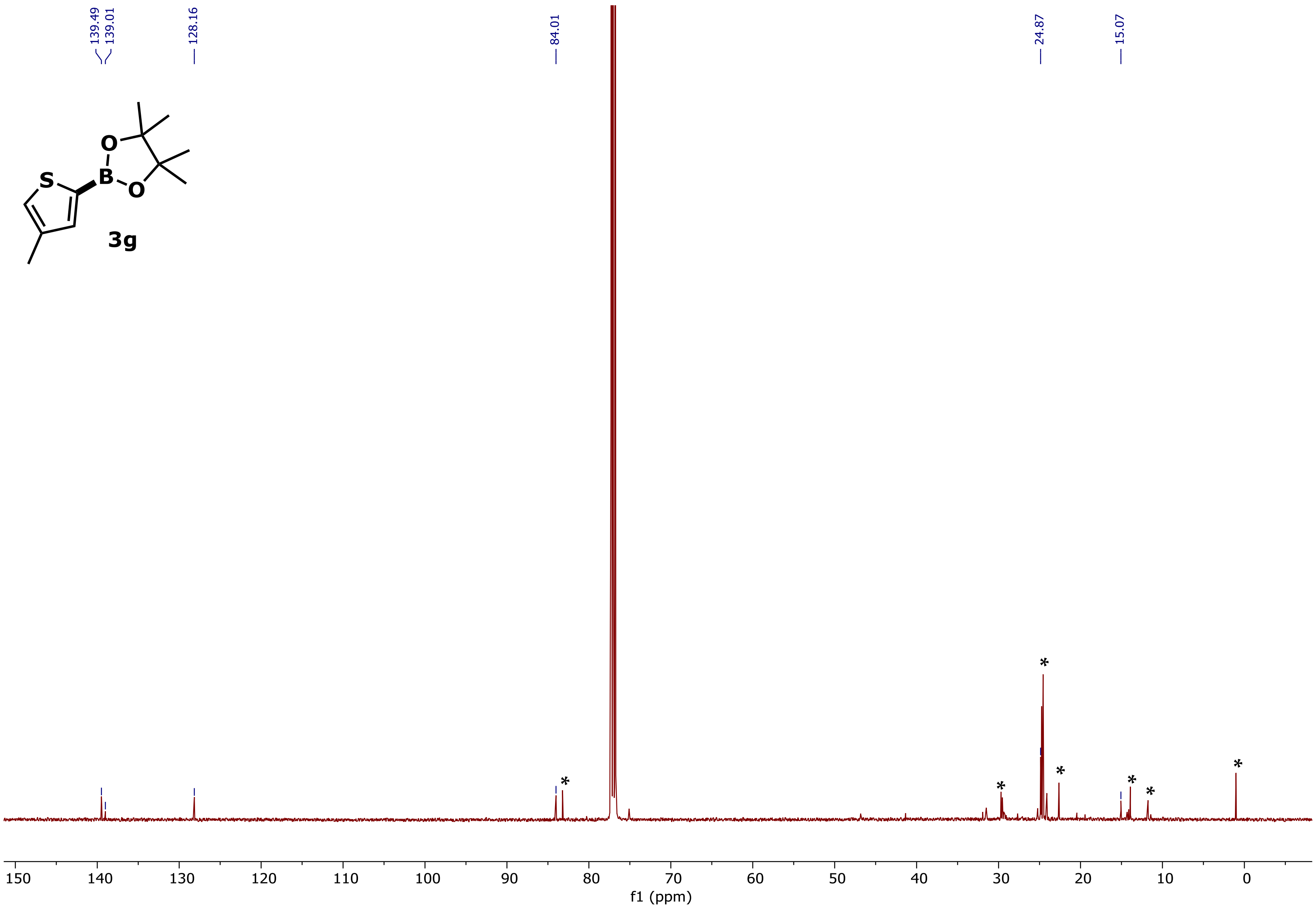

Figure S47. ^{13}C NMR (126 MHz, $CDCl_3$) 4,4,5,5-tetramethyl-2-(4-methylthiophen-2-yl)-1,3,2-dioxaborolane **3g** and *: impurity.

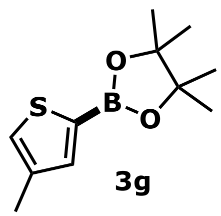

3g

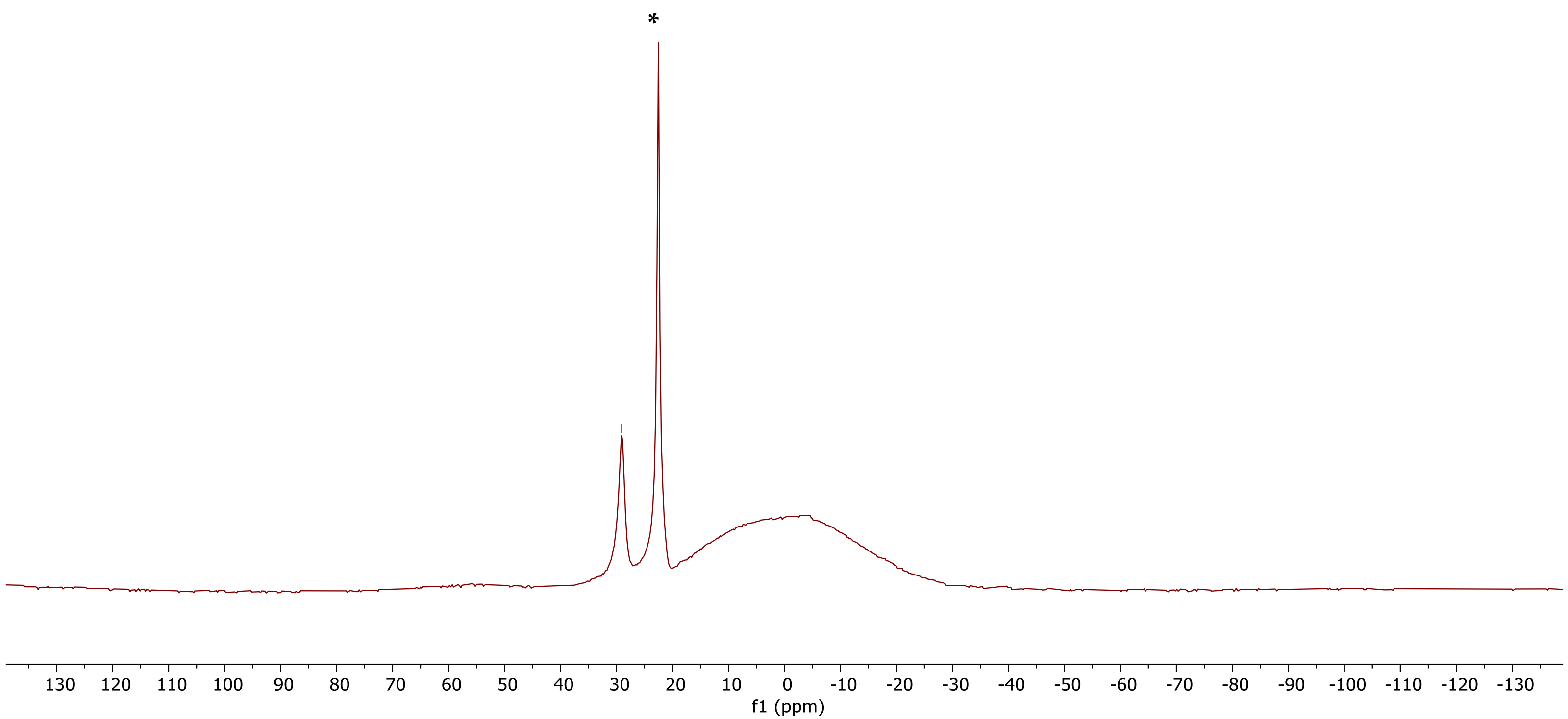

Figure S48. ^{11}B NMR (160 MHz, $CDCl_3$) 4,4,5,5-tetramethyl-2-(4-methylthiophen-2-yl)-1,3,2-dioxaborolane **3g** and *: $B(OR)_3$ impurity.
